# Toward Optimizing and Understanding Reversible Hyperpolarization of Lactate Esters Relayed from *para*-Hydrogen

**DOI:** 10.1021/acs.jpclett.2c01442

**Published:** 2022-07-21

**Authors:** Ben J. Tickner, S. Karl-Mikael Svensson, Juha Vaara, Simon B. Duckett

**Affiliations:** † Centre for Hyperpolarisation in Magnetic Resonance, Department of Chemistry, University of York, Heslington, United Kingdom, YO10 5NY; ‡ NMR Research Unit, University of Oulu, P.O. Box 3000, FI-90014, Oulu, Finland

## Abstract

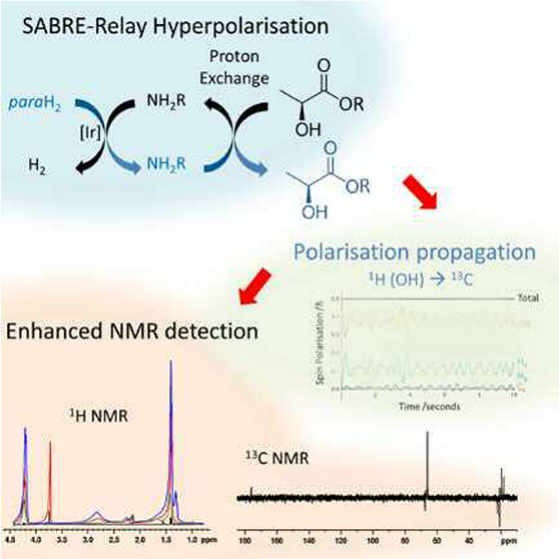

The SABRE-Relay hyperpolarization method is used to enhance the ^1^H and ^13^C NMR signals of lactate esters, which find use in a wide range of medical, pharmaceutical, and food science applications. This is achieved by the indirect relay of magnetization from *para*-hydrogen, a spin isomer of dihydrogen, to OH-containing lactate esters via a SABRE-hyperpolarized NH intermediary. This delivers ^1^H and ^13^C NMR signal enhancements as high as 245- and 985-fold, respectively, which makes the lactate esters far more detectable using NMR. DFT-calculated *J*-couplings and spin dynamics simulations indicate that, while polarization can be transferred from the lactate OH to other ^1^H nuclei via the *J*-coupling network, incoherent mechanisms are needed to polarize the ^13^C nuclei at the 6.5 mT transfer field used. The resulting sensitivity boost is predicted to be of great benefit for the NMR detection and quantification of low concentrations (<mM) of lactate esters and could provide a useful precursor for the production of hyperpolarized lactate, a key metabolite.

Lactate and lactate esters have many uses in medicine, biochemistry, and food science.^1−3^ Detection of these molecules is vital for many applications from food quality control to early diagnosis and treatment of diseases such as cancer.^4−8^ Nuclear magnetic resonance (NMR) is often the method-of-choice for molecular analysis as it allows direct detection of a molecule without sample destruction. However, NMR suffers from a high detection limit, which is usually compensated by signal averaging or using highly concentrated molecules (millimolar to molar) to generate sufficient signals. Many approaches have been developed to improve the sensitivity of NMR: perhaps the most successful is the advent of hyperpolarization.^9^ Such techniques create molecules where the normal Boltzmann spin state population distribution is tipped to create a transient spin state that can be detected more easily.

In this work, we focus on hyperpolarization involving *para*-hydrogen (*p*H_2_), a spin isomer of dihydrogen, as it is a versatile platform for the hyperpolarization of many biologically relevant disease markers.^5,10−13^ Specifically, the signal amplification by reversible exchange (SABRE) method is employed here to enhance the NMR signals of lactate esters. SABRE utilizes the oxidative addition of *p*H_2_ to form a hyperpolarized iridium dihydride complex (Figure 1a). Magnetization can then be transferred to other ligands within the complex in a process that is either spontaneous at low magnetic fields (mT^14^ and μT^15^) or radiofrequency-driven at high field (T).^16^ Spontaneous transfer at high fields (T) is also possible,^16−19^ but ∼6.5 mT fields are typically employed for most efficient polarization transfer to the ^1^H sites.^14,20^ Under such conditions, polarization transfer occurs through the temporary *J*-coupled network formed within the transient organometallic complex^21^ and level anticrossing descriptions are used to rationalize these effects.^22,23^ Subsequently, dissociation of ligands from the polarization transfer catalyst gives rise to hyperpolarized molecules free in solution. Therefore, SABRE catalytically creates hyperpolarized target molecules continually and allows them to regenerate easily after they relax back to the non-hyperpolarized form. These considerations provide advantages when compared to other hyperpolarization techniques that are one-shot in nature. Furthermore, SABRE does not require target molecules to contain *p*H_2_ acceptors or specialist equipment as its *p*H_2_ feedstock is cheap and easy to produce.^24^

**Figure 1:**
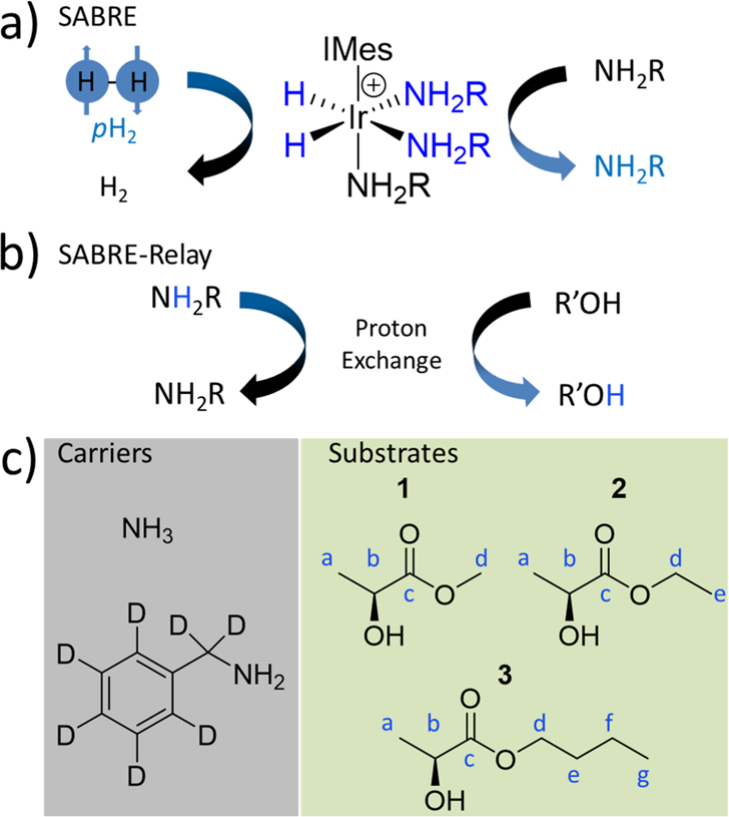
Summary of the SABRE and SABRE-Relay hyperpolarization methods and the substrates used in this work. (a) SABRE hyperpolarizes molecules without chemical alteration if the target molecule (here, NH_2_R) and *p*H_2_ are in reversible exchange at a polarization transfer catalyst. (b) The variant, SABRE-Relay exploits relayed proton exchange from a hyperpolarized source to enhance NMR signals in a target that is unable to coordinate to a SABRE catalyst. (c) The hyperpolarization carriers and hyperpolarization targets used in this work.

Currently, SABRE hyperpolarization is typically applied to substrates containing N-donor atoms, such as N-heterocycles,^14,20,25−27^ amines,^28^ and nitriles^29,30^ although S-donor and O-donor targets, such as S-heterocycles^31^ and ketoacids,^32,33^ have also been hyperpolarized. Recently, relayed proton exchange effects (such as SABRE-Relay)^34^ have expanded the substrate scope even further. In SABRE-Relay, a hyperpolarization carrier is used to transfer enhanced magnetization to a target molecule by proton exchange (Figure 1b). Amines or ammonia are typically used as hyperpolarization carriers as they have the N-donor motif required to form active [Ir(H)_2_(NHC)(NH_2_R)_3_]Cl catalysts necessary for the hyperpolarization of an amine or ammonia.^28,34,35^ Exchange between carrier NH and OH protons in molecules, such as alcohols,^36^ sugars,^37^ silanols,^38^ and many others^34,39^ allows them to become hyperpolarized even though they do not have the typical iridium-ligating motifs required for direct SABRE. Most examples of molecules hyperpolarized using SABRE-Relay are simple OH-containing structures, with sugars representing perhaps the most complex targets hyperpolarized to date. So far, SABRE-Relay has achieved 2.6% ^1^H,^36^ 2.3% ^29^Si,^38^ 1.1% ^13^C,^36,37^ 0.2% ^19^F,^36^ and 0.04% ^31^P^36^ polarization levels. In this work, we aim to expand SABRE-Relay to hyperpolarize molecules of greater biological relevance such as the OH-containing lactate esters methyl lactate (**1**), ethyl lactate (**2**), and butyl lactate (**3**) (Figure 1c). Key factors that determine the efficiency of SABRE-Relay and how polarization spreads from the exchanging OH group to ^1^H and ^13^C sites within the target substrates are explored. We predict that this sensitivity boost will be of great use for the rapid NMR detection of lactate esters and could have implications for mixture analysis and disease diagnosis in the future.

This work begins with the formation of [Ir(H)_2_(IMes)(NH_3_)_3_]Cl and [Ir(H)_2_(IMes)(NH_2_Bn-*d*_7_)_3_]Cl catalysts (where IMes is 1,3-bis(2,4,6-trimethyl-phenyl)imidazole-2-ylidene) by reaction of [IrCl(COD)(IMes)] (5 mM) (where COD is *cis*,*cis*-1,5-cyclooctadiene) with NH_3_ (8–12 equiv) or BnNH_2_-*d*_7_ (5 equiv), respectively, alongside H_2_ (3 bar) in dichloromethane-*d*_2_ (0.6 mL) at room temperature overnight. NH_3_ and BnNH_2_-*d*_7_ were selected as hyperpolarization carriers as they have been reported to yield the highest SABRE-Relay hyperpolarization levels in simple alcohols based on studies on a range of 24 different NH_2_R carriers.^36^ The formation of these polarization transfer catalysts is confirmed by the observation of characteristic ^1^H NMR resonances.^28,34^ At this point, **1**, **2**, or **3** (5 equiv) were added to the resulting solutions, under an N_2_ atmosphere, before *p*H_2_ (3 bar) was introduced. The NMR tube was then shaken manually for 10 s in the stray field of a 9.4 T spectrometer where the field was ∼6.5 mT. The metal: carrier and carrier: substrate ratios were fixed at 1:5–10 and 1:1, respectively, as these conditions have been reported to give the optimum relayed hyperpolarization levels in related substrates.^36,37^ After shaking, the samples are rapidly inserted into the 9.4 T spectrometer for the collection of a single-scan hyperpolarized ^1^H or ^13^C NMR spectrum.

When such SABRE-Relay experiments are performed, enhanced ^1^H NMR signals for **1**, **2**, or **3** can be observed (Figure 2a and b). These enhanced signals are broadened relative to their thermally polarized counterparts and this is likely related to sample movement and nonoptimal shimming using this manual sample shaking and insertion method. The use of automated systems could reflect a route to reduce line broadness.^40,41^ The highest ^1^H NMR signal enhancements were seen for the CH group or lactate CH_3_ group, with polarization transfer across the ester group into the methyl, ethyl, or butyl groups in **1**, **2**, or **3**, respectively, being less significant (Table 1). Prior relaxation of the associated nonequilibrium population differences will play an important role in determining these measured NMR signal enhancements and therefore the relaxation times of the ^1^H resonances of **1**–**3** in these mixtures were determined (Table 1). It might be expected that the aliphatic sites with the highest NMR signal enhancements are associated with long *T*_1_ values. This is true for **1** in which the CH site with the longest *T*_1_ time (29.3 s) has the highest ^1^H NMR signal enhancement (55-fold). However, these trends are not always consistent as the lactate CH_3_ group within **1** contains the shortest *T*_1_ within the molecule (5.9 s) and yet exhibits a modest 30-fold ^1^H NMR signal enhancement. In other words, the low ^1^H NMR signal enhancements of sites in the ester chains of **1**–**3** are not necessarily just associated with rapid proton relaxation (Table 1). When these experiments were repeated using the BnNH_2_-*d*_7_ carrier, the trends in ^1^H NMR signal enhancement proved similar, with the polarization gain remaining predominantly localized on the lactate CH and CH_3_ groups. For the ^1^H NMR signal enhancements of these substrates, BnNH_2_-*d*_7_ proves to be more efficient than NH_3_ as it gives rise to higher enhancements for every site of **1** and **2** when compared to those with NH_3_, whereas the ^1^H NMR signal enhancements of **3** are comparable for both carriers. Previous investigations have suggested that when NH_3_ and BnNH_2_-*d*_7_ are compared as SABRE targets, greater NH polarization results for BnNH_2_-*d*_7_.^28,36^ We, therefore, link the amount of substrate OH polarization to the finite polarization of the carrier NH available for relay into the lactate esters after exchange. It generally appears that the average ^1^H NMR signal enhancements per site decrease as the length of the ester chain increases in accordance with the fact that the finite OH polarization is shared among more protons. Greater distances between the exchanging OH group and remote aliphatic sites in the ester side arm are also likely to play a role in reducing the efficiency of polarization transfer to distant sites.^12,42,43^

**Figure 2:**
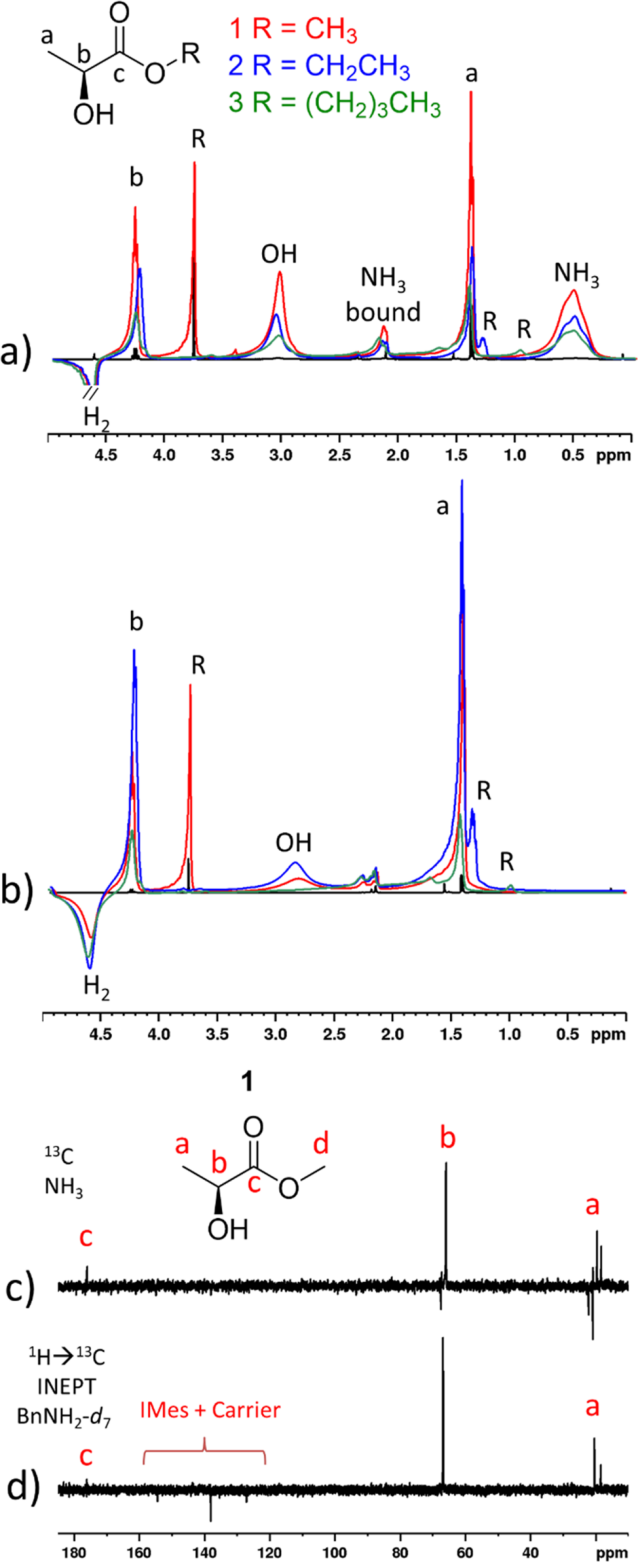
Hyperpolarized NMR spectra. Selected regions of single-scan (a, b) ^1^H, (c) ^13^C, and (d) ^1^H decoupled ^13^C INEPT (short-range) NMR spectra recorded at 9.4 T and 298 K after samples containing (a, c) NH_3_ (8–12 equiv) or (b, d) BnNH_2_-*d*_7_ (5 equiv) with [IrCl(COD)(IMes)] (5 mM) and the indicated lactate ester in 0.6 mL of dichloromethane-*d*_2_ are shaken with 3-bar pH_2_ for 10 s at 6.5 mT. In panels a and b, the corresponding single-scan thermally polarized spectrum for **1** is shown in black for comparison. More detailed NMR spectra are provided in the Supporting Information, section S2. Thermally polarized ^13^C and INEPT NMR spectra are provided in the Supporting Information, section S3, for comparison.

**Table 1:** Summary of SABRE-Relay NMR Signal Enhancements^a^

					^13^C NMR signal enhancement (fold)					
		^1^H NMR signal enhancement (fold)		^1^H *T*_1_ (s)	^13^C NMR		^1^H and ^13^C INEPT short-range		^1^H and ^13^C INEPT long-range	
substrate	site	carrier NH_3_	carrier BnNH_2_-*d*_7_	carrier NH_3_	carrier NH_3_	carrier BnNH_2_-*d*_7_	carrier NH_3_	carrier BnNH_2_-*d*_7_	carrier NH_3_	carrier BnNH_2_-*d*_7_
**1**	a	30 ± 5	110 ± 10	5.9	625 ± 5	310 ± 40	175 ± 10	55 ± 5	30 ± 5	15 ± 5
	b	55 ± 5	115 ± 10	29.3	595 ± 30	335 ± 25	400 ± 55	150 ± 5	390 ± 50	70 ± 5
	c	N/A	N/A	N/A	85 ± 10	35 ± 5	0	305 ± 20	70 ± 15	30 ± 5
	d	15 ± 5	40 ± 5	10.9	0	0	0	0	0	0
	OH	80 ± 5	310 ± 10	5.8	N/A	N/A	N/A	N/A	N/A	N/A
	average	35 ± 5	85 ± 5	N/A	375 ± 10	195 ± 20	320 ± 40	115 ± 5	145 ± 20	35 ± 5
	NH (free)	70 ± 5	N/A	6.2	N/A	N/A	N/A	N/A	N/A	N/A
**2**	a	15 ± 5	135 ± 5	5.0	640 ± 5	270 ± 5	55 ± 5	55 ± 5	15 ± 5	20 ± 5
	b	15 ± 5^b^	90 ± 5^b^	12.0^b^	420 ± 45	345 ± 20	115 ± 15	185 ± 10	130 ± 40	120 ± 10
	c	N/A	N/A	N/A	85 ± 10	50 ± 5	0	335 ± 50	100 ± 30	120 ± 5
	d	15 ± 5^b^	90 ± 5^b^	12.0^b^	0	0	0	0	0	0
	e	2 ± 1	20 ± 5	8.1	0	0	0	0	0	0
	OH	40 ± 10	165 ± 5	4.8	N/A	N/A	N/A	N/A	N/A	N/A
	average	15 ± 5	80 ± 5	N/A	390 ± 20	140 ± 5	125 ± 20	140 ± 5	85 ± 20	70 ± 5
	NH (free)	30 ± 5	N/A	4.4	N/A	N/A	N/A	N/A	N/A	N/A
**3**	a	20 ± 5^b^	20 ± 5^b^	4.0^£^	120 ± 25	95 ± 5	30 ± 5	60 ± 5	0	20 ± 5
	b	40 ± 5^+^	70 ± 5^+^	12.8^+^	165 ± 35	255 ± 15	110 ± 10	5 ± 1	45 ± 15	80 ± 15
	c	N/A	N/A	N/A	35 ± 5	40 ± 5	0	3 ± 1	75 ± 5	35 ± 5
	d	10 ± 5^+^	10 ± 5^+^	5.6^+^	0	0	0	0	0	0
	e	25 ± 5	25 ± 5	5.1	0	0	0	0	0	0
	f	20 ± 5^b^	20 ± 5^b^	5.4^£^	0	0	0	0	0	0
	g	7 ± 1	5 ± 1	5.7	0	0	0	0	0	0
	OH	50 ± 5	N/A	4.7	N/A	N/A	N/A	N/A	N/A	N/A
	average	20 ± 5	20 ± 5	N/A	110 ± 20	130 ± 5	95 ± 10	15 ± 5	40 ± 5	15 ± 5
	NH (free)	75 ± 10	N/A	5.8	N/A	N/A	N/A	N/A	N/A	N/A

a ^1^H and ^13^C NMR signal enhancements (per site) for **1**–**3** when each (5 equiv) is shaken with 3-bar *p*H_2_ for 10 s at 6.5 mT in an activated solution of [IrCl(COD)(IMes)] (5 mM) with NH_3_ (8–12 equiv) or BnNH_2_-*d*_7_ (5 equiv) in dichloromethane-*d*_2_ (0.6 mL). The sites are labelled according to Figure 1c. ^1^H *T*_1_ values were measured at 9.4 T using inversion recovery. Note that the average polarization refers to the total hyperpolarized integral intensity of the substrate divided by its thermally polarized counterpart and reflects an average substrate polarization per ^1^H or ^13^C site. NH (free) refers to the polarization (per site) for the carrier NH.

b Values are averaged across the two sites due to signal overlap; ^+,£^Signals overlap.

When similar single-scan hyperpolarized ^13^C NMR measurements are recorded on the samples of **1**–**3** with NH_3_ or BnNH_2_-*d*_7_, carriers, enhanced ^13^C NMR signals can also be detected despite the fact that the lactate esters are in a non-^13^C-labeled form (Figure 2c and d, Table 1). Significant ^13^C NMR signal enhancements are seen for the lactate CH and CH_3_ (>95-fold) groups with smaller values achieved for the ester carbonyl (<85-fold). The site with the highest ^13^C NMR signal gain is now dependent on the substrate and carrier. For **1**, the lactate CH_3_ site was enhanced to greater extent than the CH site when NH_3_ was used as a carrier (625- versus 595-fold, respectively), although when BnNH_2_-*d*_7_ was employed the CH polarization was greater (310- versus 335-fold, respectively). However, for **3**, the CH site received the greatest share of polarization (165- versus 255-fold with NH_3_ and BnNH_2_-*d*_7_ carriers, respectively, compared to CH_3_ gains of 195- and 150-fold, respectively). For **2**, NH_3_ again delivered the highest ^13^C polarization on the lactate CH_3_ site when compared to the CH site (640- versus 420-fold) but BnNH_2_-*d*_7_ enhances the CH signal more than the CH_3_ (345- versus 270-fold). This behavior suggests that the OH/NH exchange rate, which will differ for the two carriers, must influence the degree of polarization transfer from OH into the ^13^C nuclei. Visible OH polarization is clearly higher using BnNH_2_-*d*_7_ when compared to NH_3_, which is reflected in generally better ^1^H performance but worse ^13^C gains. Exchange of the OH group proton will destroy the *J*-coupling network that is associated with propagation of polarization. Therefore, the residence time of the enhanced OH proton is concluded to be extremely important.^44^ This fact highlights the tension between initial carrier polarization, efficiency of the relayed NH/OH exchange, and subsequent propagation of OH polarization to other ^1^H and ^13^C sites within the substrate. At the 65 G polarization transfer fields in these experiments, ^13^C polarization was localized exclusively within the lactate unit and did not spread across the carbonyl group to the ester chains of the substrates. It is worth noting that transfer to distant ^13^C sites may be possible using field cycling regimes at low magnetic fields (i.e., μT): such effects have been demonstrated for related molecules.^5,12,42^ For utilization of these enhanced ^13^C MR signals, it is important that hyperpolarization resides most predominantly on sites with the longest relaxation time as this allows the hyperpolarized state to be detected over much longer time windows which is useful for reaction monitoring^45,46^ or biomedical imaging applications.^47^ Estimates of hyperpolarized ^13^C magnetization lifetimes of **1**–**3** were measured at 9.4 T by leaving the hyperpolarized sample (with carrier and catalyst) in the magnet for a varying time interval before recording a single-scan ^13^C NMR spectrum. This yielded *T*_1_ values of ∼<5, ∼20, and *∼*50 s for the CH_3_, CH, and CO sites, respectively (see Figure S17). These compare well with values reported in the literature for lactate and similar molecules and confirm that the carbonyl carbon, which is more isolated from the other NMR-active spins within the molecule, has the longest *T*_1_.^5,6,48^ Unfortunately, in our SABRE-Relay experiments on **1**–**3** discussed so far the majority of the attained ^13^C polarization resides on the more rapidly relaxing CH_3_ and CH groups and only modest (<100-fold) signal enhancements can be achieved for the slowly relaxing CO site.

To enhance the degree of magnetization transfer to the carbonyl site, ^1^H → ^13^C INEPT (insensitive nuclei enhanced by polarization transfer) pulse sequences were used to transfer the SABRE-Relay derived ^1^H polarization to ^13^C sites in the molecule. These sequences contain a variable time delay that is related to the size of the *J*-coupling connecting the ^1^H–^13^C spin pair between which magnetization is transferred. NMR pulse sequences were used with two sets of arbitrary time delays corresponding to polarization transfer between ^1^H–^13^C spin pairs with 125 Hz (short-range) and 10 Hz (long-range) coupling (see Supporting Information, section S1.2). The effect of these ^1^H → ^13^C INEPT sequences, following the hyperpolarization process of **1**–**3** using SABRE-Relay, was examined by calculating the ^13^C NMR signal enhancements by reference to thermally polarized ^1^H → ^13^C INEPT sequences recorded using the same delay times and acquisition parameters (Table 1). ^13^C NMR signal enhancements for the lactate CH and CH_3_ sites within **1**–**3** were higher using direct ^13^C NMR detection as compared to the ^1^H → ^13^C INEPT detection, regardless of the carrier or INEPT sequence used (Table 1). Optimized INEPT sequences can theoretically boost ^13^C polarization by a factor of 4, which reflects the four times larger gyromagnetic ratio of ^1^H nuclei. The INEPT ^13^C NMR signal enhancements recorded here do not achieve this level of ^13^C sensitivity boost (Table 1). We expect factors, such as OH exchange and relaxation, during the mixing time of these INEPT experiments to decrease their efficiency, effects that become more evident for “short-range” INEPT sequences containing longer time delays. In our experiments, it is challenging to attribute a combined ^13^C NMR signal enhancement factor resulting from combined SABRE-Relay and INEPT methods due to differing acquisition parameters. Nevertheless, we suggest that true hyperpolarized ^13^C INEPT NMR signal enhancements compared to thermally polarized direct ^13^C NMR detection are ∼1–2 times larger than the signal enhancements presented in Table 1, which quotes values relative to thermally polarized ^1^H → ^13^C INEPT control measurements. There is, however, a significant uplift in the CO polarization level of the longer-lived carbonyl sites. For instance, 305- and 335-fold ^13^C NMR signal enhancements could be achieved for **1** and **2** respectively using the BnNH_2_-*d*_7_ carrier and short-range ^1^H → ^13^C INEPT sequences, which is higher than the 35-fold and 50-fold achieved for the same samples with standard ^13^C NMR detection. Interestingly, this increase in carbonyl site ^13^C polarization using the short-range INEPT sequence suggests that polarization transfer from OH to CO sites is now efficient even though the *J* coupling is mismatched. There is, however, a dramatic difference between differing carriers, which suggests that the OH/NH exchange has a major impact. For example, in samples involving NH_3_ as a carrier, ^13^C NMR signal enhancements for the carbonyl site of **2** and **3** detected using INEPT sequences optimized for long-range ^1^H–^13^C couplings (10 Hz) were higher when compared to direct ^13^C NMR detection. However, for samples containing BnNH_2_-*d*_7_, use of short-range (125 Hz) ^1^H → ^13^C INEPT sequences yielded much better ^13^C NMR carbonyl signal enhancement in **1**–**3** when compared to direct ^13^C NMR detection. These results highlight that there is no one set of standard conditions that deliver optimal SABRE-Relay NMR signal enhancements in all three substrates and suggest that further development and the use of novel sequences^49−52^ with optimized delay times may constitute a valuable route to increase these ^13^C signal gains further.

The initial SABRE effect, in which spin order is transferred from *p*H_2_-derived hydride ligands to the NH site of a bound carrier molecule, is driven by formation of a transient *J*-coupled network.^21,22,53^ Upon NH/OH exchange, polarization propagation to other ^1^H and ^13^C sites within the lactate ester could occur through *J*-coupling in a similar fashion. To gain a greater understanding of these effects, density functional theory (DFT) calculations were performed to predict the *J*-couplings within **1**-**3** that are involved in these polarization transfer processes (Supporting Information, Tables S5–7). Overall, the short-range computed coupling constants are in fairly good agreement with values obtained from experiment (Supporting Information, Tables S5–7). Furthermore, these calculations determine long-range *J*-coupling values that we could not discern from ^1^H or ^13^C NMR spectra and could play an important role in polarization propagation. The results show that the *J* coupling between the OH proton and the carbonyl carbon is just 5–6 Hz. Small *J*-couplings will also play a role if polarization transfer from OH is indirectly propagated to the carbonyl carbon according to a OH → H_b_ → C_c_ pathway, with the latter step no longer being inhibited by OH exchange. Computationally, the OH → H_b_ → C_c_ transfer pathway involves small *J* couplings of <5 Hz. A route involving a larger 56–57 Hz coupling C_b_ → C_c_ could operate, but this will be limited by the natural ^13^C abundance and a large *J*_CC_ splitting is not observed for the carbonyl signal in hyperpolarized spectra. Crucially, these *J*-couplings prove to be similar for **1**–**3**, which suggests that the differences in substrate ^1^H and ^13^C polarization levels that are observed are not due to significant differences in the spin coupling topology within these CH frameworks.

Spin dynamics simulations were performed to predict how coherent polarization transfer might occur via the *J*-coupling network from the OH spin to the other spins within the lactate ester framework. Different spin systems were simulated, taking into account the fact that natural abundance ^13^C was involved in the experiments. Initially, a 5-spin model consisting of OH, H_b_, and three H_a_ protons was used to study polarization transfer within the proton-only spin system. These simulations allow evolution of an initial 100% OH nuclear spin polarization during a 10-s shaking period at a given polarization transfer field (0 T, 6.5 mT, or 9.4 T) to the other ^1^H spins. These simulations reflect an ideal experiment free from the effects of OH/NH exchange and *T*_1_ relaxation. Simulations for this system show that spin polarization transfer occurs between all the included ^1^H spins at the lower polarization transfer fields (0 T and 6.5 mT), but essentially no spin polarization transfer occurs at 9.4 T (see Supporting Information, Figures S19–S21). Furthermore, 6-spin systems involving one of the ^13^C_a_, ^13^C_b_, or ^13^C_c_ centers were simulated to investigate polarization transfer to ^13^C, as in most ^13^C-containing molecules there is only one naturally abundant ^13^C nucleus (see Figure 3 and Supporting Information, Figures S19–S21). Sites in the ester side chain were not included as ^13^C NMR experiments do not exhibit enhanced magnetization for these sites at a 6.5 mT transfer field. For these 6-spin systems, simulations show that at polarization transfer fields of 0 and 9.4 T they behave similarly to the 5-spin ^1^H systems, with spin polarization transfer to all spins at 0 T and not to any noticeable extent to any spin at 9.4 T. At a polarization transfer field of 6.5 mT, the simulations show that the presence of a ^13^C (i.e., in about 1% of the molecules at natural abundance), reduces the spin polarization transfer between the protons by perturbing the spin–spin coupling network, without any noticeable spin polarization being transferred to the ^13^C. Under such idealized conditions, the simulations reveal that coherent polarization transfer from OH to H_a_ and H_b_ can occur at 6.5 mT, but transfer to the ^13^C subsystem does not. This suggests that other, incoherent routes, such as cross relaxation, have to dominate the OH → ^13^C polarization propagation at the 6.5 mT polarization transfer fields necessary to achieve optimal ^1^H polarization via SABRE-Relay. This finding supports previous theoretical studies.^44^

**Figure 3:**
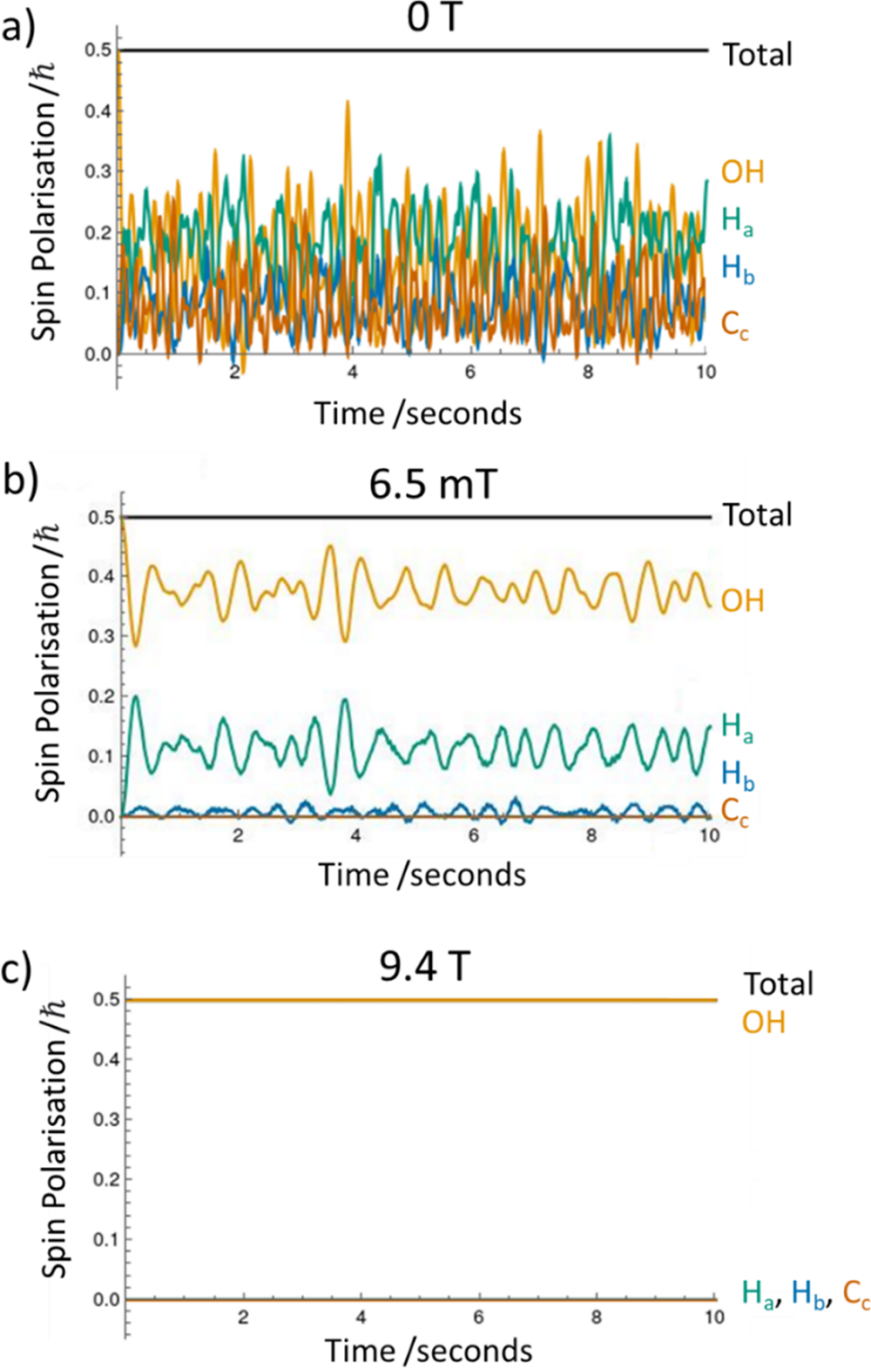
Simulating polarization propagation. Simulated spin polarization as a function of time for the different sites of a 6-spin system (3H_a_, H_b_, OH, and C_c_) at (a) 0 T, (b) 6.5 mT, and (c) 9.4 T.

The results presented so far highlight that there is no one set of standard conditions that achieve optimal SABRE-Relay NMR signal enhancements for all substrates. Optimization of magnetization on a particular site, or particular nuclei (^1^H or ^13^C in this work), is likely to require a different set of conditions, even for optimization of NMR signal enhancements on different sites, or for different nuclei, in the same molecule using the same carrier. To highlight this point, SABRE-Relay hyperpolarization of **2** was tested with different amounts of the carrier NH_3_. These results show that the ^1^H NMR signal enhancements for the lactate CH_3_, CH and OH sites could be dramatically increased from 15-, 15-, and 40-fold using 9 equiv NH_3_ to 215-, 160-, and 245-fold, when the amount of NH_3_ was lowered to 6.5 equiv (Figure 4a). Similar increases of ^13^C NMR signal enhancements from 640-, 420, and 85-fold for the CH_3_, CH, and CO sites, respectively, to 865-, 985- and 110-fold, respectively (Figure 4b), were observed. Interestingly, this same improvement in ^13^C NMR signal enhancement at lower NH_3_ concentration was not observed in the data collected by ^1^H → ^13^C INEPT transfer (Figure 4c and d). This suggests that a lower NH_3_ amount corresponds to more optimal, likely slower, NH/OH exchange and benefits the direct ^1^H and ^13^C NMR detection.

**Figure 4:**
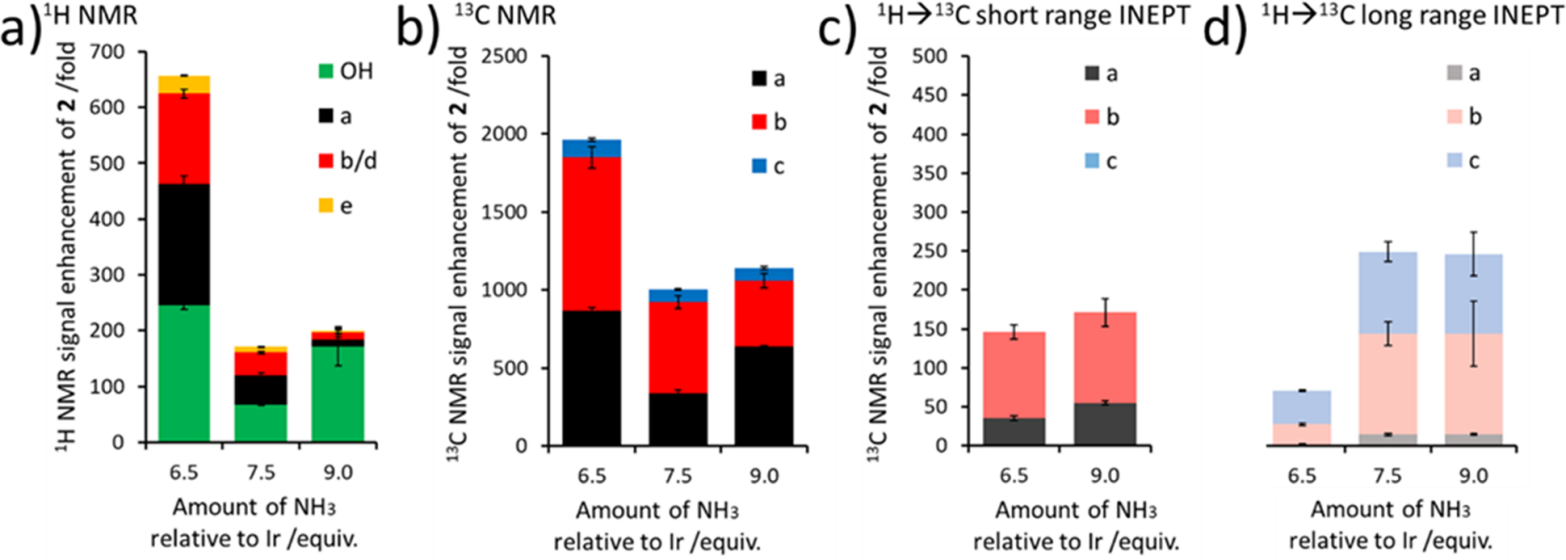
Toward optimizing SABRE-Relay. (a) ^1^H and (b) ^13^C NMR signal enhancements of **2** achieved using SABRE-Relay as a function of carrier NH_3_ concentration. ^13^C NMR signal enhancements achieved using (c) short-range and (d) long-range ^1^H→ ^13^C INEPT sequences are also shown. A full table of NMR signal enhancements is given in the Supporting Information, Table S9.

The substrates **1**–**3** used in this work are liquids at room temperature and pressure. Hyperpolarization of protic molecules that are not soluble in nonprotic SABRE-Relay compatible solvents can be challenging because nonspecific proton exchange is undesirable. For example, attempts to hyperpolarize sodium lactate by SABRE-Relay fail due to the presence of water; 10 μL is required to solubilize it in CD_2_Cl_2_ or CDCl_3_ solvents (see Supporting Information, section S10). Furthermore, doping SABRE-Relay samples of **1**–**3** with water leads to a drop in the delivered NMR signal enhancements (see Supporting Information, section S9). Similar effects have been reported for other substrates.^36,37^ Hyperpolarization of **1**-**3** could therefore provide a route to hyperpolarize sodium lactate. To this end, SABRE-Relay could be used with rapid hydrolysis of the ester side chain to form lactate in an approach that is similar to PHIP-SAH which has already achieved significant lactate ^13^C polarization.^5^ In preliminary experiments we confirmed that hydrolysis and phase separation of **2** could indeed yield aqueous solutions of lactate (see Supporting Information, section S10). However, these steps took ∼90 s in our nonoptimized setup and, therefore, have not yet yielded enhanced NMR signals for lactate. In the future, optimization of ^13^C NMR signal gains on the carbonyl site, followed by rapid hydrolysis, will likely open a route to produce hyperpolarized lactate. This approach could be extended to a wider range of molecules that contain the OH-groups necessary for SABRE-Relay but are not soluble in compatible solvents. They could be functionalized as esters with a side arm that alters solubility. This would allow them to be hyperpolarized using SABRE-Relay before the target molecule is released in a rapid hydrolysis step.

In conclusion, hyperpolarization of lactate esters using SABRE-Relay has been presented. ^1^H and ^13^C polarization is predominantly localized within the lactate CH and CH_3_ groups with lower signal enhancements for the carbonyl carbon and little polarization transfer to the ester side arm. Optimization and rationalization of SABRE-Relay efficiency can be extremely challenging with many factors such as carrier NH polarization, OH/NH exchange efficiency, and polarization propagation from OH to other sites with the substrate all being important steps that can be influenced by the carrier, substrate, and their ratios. ^1^H and ^13^C NMR enhancements as high as 245-fold (0.8% polarization) and 985-fold (0.8% polarization), respectively, for lactate esters have been achieved using the SABRE-Relay approach. We have also demonstrated that ^1^H → ^13^C INEPT sequences can in some cases improve polarization of the longer-lived carbonyl site (up to 335-fold ^13^C NMR enhancement using hyperpolarized INEPT). These sensitivity improvements have allowed the detection of 25 mM concentrations of lactate esters in just a single-scan ^13^C NMR spectrum, which are not discerned using thermally polarized NMR. Examination of a wider range of INEPT sequences,^6,49,54,55^ further optimization of sequence mixing times, or even magnetic field cycling approaches,^5,42^ are likely to increase these NMR signal enhancements. Relayed substrate/carrier exchange influences polarization propagation within the substrate, despite theoretical calculations showing similar spin topologies within the substrates used here. Spin dynamics simulations indicate that polarization transfer from the OH proton to the other ^1^H sites of the substrate readily takes place via the spin–spin coupling network at the polarization transfer field of 6.5 mT. In contrast, polarization of the ^13^C spin subsystem is deduced to occur via incoherent mechanisms, at this polarization transfer field. It is likely that both *J*-coupling and cross-relaxation effects can play a role in polarization transfer to heteronuclei depending on the spin topology of the substrate and the polarization transfer field. In the future, further studies to understand and optimize relayed polarization transfer would be highly beneficial because of the wider range of molecules whose NMR signals could be enhanced by the technique.

## Supporting Information Available

Experimental methods, computational details, additional NMR spectra (PDF)
Code for simulation of polarization transfer and NMR spectra (PDF, ZIP)

## Notes

Data can be found at https://pure.york.ac.uk/portal/en/.
