# Supplementary material for: Toward Optimizing and Understanding Reversible Hyperpolarization of Lactate Esters Relayed from para-Hydrogen
Source: J Phys Chem Lett. 2022 Jul 21;13(29):6859–66. doi: 10.1021/acs.jpclett.2c01442 (PMC9340809; doi:10.1021/acs.jpclett.2c01442)
Supplement: Supplementary file 1 — jz2c01442_si_001.pdf [file jz2c01442_si_001.pdf]

Supporting Information

Towards Optimising and Understanding Reversible Hyperpolarisation of Lactate Esters Relayed from *Para*hydrogen

Ben. J. Tickner, [a,b] *S. Karl-Mikael Svensson,* [b] *Juha Vaara,*[b] *and Simon B. Duckett*[a]*

[a]*Centre for Hyperpolarisation in Magnetic Resonance, Department of Chemistry, University of York, Heslington, York, YO10 5NY, United Kingdom*

[b]*NMR Research Unit, University of Oulu, P.O. Box 3000, FI-90014, Oulu, Finland*

**Corresponding Author Email address:* simon.duckett@york.ac.uk

Table of Contents

S1: Experimental

S1.1 Preparation of SABRE polarisation transfer catalysts

S1.2 SABRE-Relay hyperpolarisation experiments

S1.3 Calculation of NMR signal enhancements

S1.4 Computational details

S2: NMR resonances of 1-3

S3: ^{1}H NMR signal enhancements of 1-3

S4: ^{13}C NMR signal enhancements of 1-3

S3.1 Single 90° pulse ^{13}C NMR detection

S3.2 ^{1}H → ^{13}C INEPT short-range (125 Hz)

S3.3 ^{1}H → ^{13}C INEPT short-range (10 Hz)

S5: Hyperpolarised ^{13}C T_1 determination

S6: DFT calculation of optimised geometries and *J*-coupling constants of 1-3

S7: Theoretical insight into polarisation transfer

S6.1 Simulated polarisation transfer

S6.2 Simulated ^{1}H NMR spectra

S6.3 Simulated ^{1}H NMR signal enhancements

S8: Effect of NH_3 concentration on ^{1}H and ^{13}C NMR signal enhancements of 2

S9: Effect of added H_2O on ^{1}H and ^{13}C NMR signal enhancements of 2

S10: Towards hyperpolarisation of sodium lactate

S11: References

S1: Experimental

All NMR measurements were carried out on a 400 MHz Bruker Avance III spectrometer at room temperature (298 K). ^{1}H (400.1 MHz) and ^{13}C (100.6 MHz) NMR spectra were recorded with an internal deuterium lock. Chemical shifts are quoted as parts per million and referenced to residual C*H*DCl$_2$ as the solvent. ^{13}C NMR spectra were recorded without broadband proton decoupling. Coupling constants (J) are quoted as absolute values in Hertz. ^{1}H T_1 relaxation measurements were recorded using a standard inversion-recovery pulse sequence.

All starting materials were purchased from Sigma Aldrich and used as purchased without additional purification. The [IrCl(COD)(IMes)] precatalyst (where IMes = 1,3-bis(2,4,6-trimethylphenyl)imidazole-2-ylidene and COD = *cis*,*cis*-1,5-cyclooctadiene) was synthesised by Dr. Victoria Annis according to a literature procedure.[1] *Para*hydrogen (pH$_2$) was produced by passing hydrogen gas over a spin-exchange catalyst (Fe_2O_3) at 28 K and used for all the hyperpolarisation experiments. This method produces pH$_2$ with *ca.* 98% purity. The dichloromethane-d_2 solvent was dried further by addition of activated molecular sieves (3 Å) and storing in a glove box for >1 week. Samples were prepared in a glove box to exclude H_2O from the atmosphere.

S1.1 Preparation of SABRE polarisation transfer catalysts

For SABRE-Relay experiments, samples were prepared containing [IrCl(COD)(IMes)] (5 mM) and the indicated carrier (NH_3 or BnNH$_2$-d_7) in 0.6 mL of dichloromethane-d_2 in a 5 mm NMR tube that was fitted with a J. Young's tap. In the case of BnNH$_2$-d_7, the required amount was simply added to the sample as a liquid using a pipette. NH_3 was added to the samples as a gas using a Schlenk line. The NMR tube containing the catalyst in DCM-d_2 and a cannister of NH_3 gas were both attached to a high vacuum line. The NMR tube was degassed and the system is placed under vacuum before the NH_3 cannister is opened to allow the gas to transfer to the NMR tube. The amount of NH_3 dissolved in solution was then quantified using ^{1}H NMR spectroscopy by comparison of the integral intensity of the NH_3 resonance at *ca* δ 0.5 ppm to those of the COD ligand of [IrCl(COD)(IMes)] at δ 3 and 4 ppm. More ammonia could be added by repeating the addition process, excess ammonia could be removed by opening the tube to the atmosphere and bubbling N_2 gas through the solution. When the NH_3 was present in the desired amount the total volume was readjusted to 0.6 mL (if necessary) and the sample was degassed. All samples were degassed by at least two freeze-pump-thaw cycles on a high vacuum line before filling the tubes with H_2 at 3-bar pressure and leaving at overnight room temperature to allow the formation of [Ir(H)$_2$(IMes)(NH$_3$)$_3$]Cl or [Ir(H)$_2$(IMes)(BnNH$_2$-d_7)$_3$]Cl. The formation of these polarisation transfer catalysts is confirmed by the presence of a single ^{1}H NMR resonance in the hydride region between δ −22 and −25 ppm.[2,3] At this point no ^{1}H NMR signals for the COD ligand of the precatalyst at δ 3 and 4 ppm were present and a signal of cyclooctane (δ 1.5 ppm) was visible. This transformation is accompanied by a colour change from orange to colourless.

Once the formation of [Ir(H)$_2$(IMes)(NH$_3$)$_3$]Cl or [Ir(H)$_2$(IMes)(BnNH$_2$-d_7)$_3$]Cl was confirmed, the substrate (5 equiv.) was added by removing the J. Young's tap and pipetting the liquid into the NMR tube under a flow of N_2 gas to exclude oxygen. The tube lid was replaced, the N_2 atmosphere removed using a high vacuum line, and pH$_2$ (3 bar) was added. At this point, the samples were ready for hyperpolarisation experiments. We note that similar results are obtained when substrate is added to the mixture from the beginning.

S1.2 SABRE-Relay hyperpolarisation experiments

The shake & drop method was employed for recording hyperpolarised NMR spectra. Once filled with pH$_2$ (3 bar), the NMR tubes were shaken vigorously (manually) for 10 seconds in the 6.5 mT fringe field of a 9.4 T Bruker spectrometer. Immediately after shaking, the NMR tubes were rapidly placed inside the spectrometer for NMR detection using a single 90° pulse for either ^{1}H or ^{13}C NMR detection. Pulse sequences were modified to contain an 'autosuspend' function immediately before the 90° pulse allowing spectral acquisition to occur immediately after sample acquisition using a 'resume' command. Hyperpolarised ^{13}C NMR spectra were recorded without proton decoupling. ^{1}H→^{13}C INEPT sequences are detailed in Figure S1 and were recorded with ^{1}H decoupling.

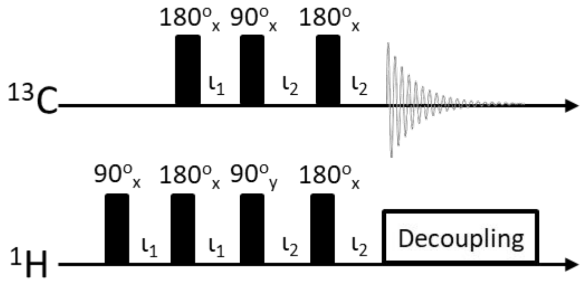

Figure S1. ^{1}Hà^{13}C INEPT sequences used in this work. The time delays are defined using equations 1 and 2.

Time delays (ι_1 and ι_2) were calculated according to equations 1 and 2 where J_{HC} is the J coupling between the ^{1}H and ^{13}C spin pair between which magnetisation is transferred.

$$\iota_1 = \frac{1}{4 \times J_{HC}} \quad (1)$$

$$\iota_2 = \frac{1}{6 \times J_{HC}} \quad (2)$$

'Short-range' and 'long-range' INEPT sequences were used based on 125 Hz and 10 Hz couplings respectively. These correspond to delay times of 2 ms and 1.33 ms for short-range INEPT sequences and 25 ms and 16.66 ms for long-range INEPT sequences.

S1.3 Calculation of NMR signal enhancements

^{1}H NMR signal enhancements were calculated by dividing the hyperpolarised ^{1}H NMR signal intensity by the corresponding ^{1}H NMR signal intensity in a thermal spectrum. The thermal and hyperpolarised spectra are recorded on the same sample using the same acquisition and spectral parameters and the thermal sample was left inside the magnet for sufficient time to allow equilibration with the spectrometer magnetic field. Three repeat measurements were taken and these values were averaged and an error was calculated. In the case of ^{13}C NMR signal enhancements, single-scan thermally polarised spectra were also recorded for comparison, but these spectra often do not show ^{13}C NMR signals for **1**-**3** due to the low signal intensity. Therefore, ^{13}C NMR signal enhancements were calculated by reference to 32-scan thermally polarised measurements for separate samples containing more concentrated **1**-**3** (0.1 mL) in dichloromethane-d_2 (0.5 mL). These thermally polarised measurements used the same acquisition parameters as the hyperpolarised measurements and long repetition delays were left between scans. ^{13}C NMR signal enhancements were then calculated using equation 3 where S_{Hyp} is the hyperpolarised integral intensity, V_{Therm} is the volume of substrate in the thermally polarised sample (0.1 mL), NS_{Therm} = 32 and is the number of scans in the thermally polarised reference, S_{Therm} is the thermally polarised NMR integral intensity and V_{Hyp} is the volume of substrate in the hyperpolarised sample.

$$\frac{S_{Hyp}V_{Therm}NS_{Therm}}{S_{Therm}V_{Hyp}} \quad (3)$$

All NMR signal enhancements (^{1}H and ^{13}C) are given per site as absolute values ('in fold') and are rounded to the nearest five, unless smaller than 10-fold. ^{13}C NMR signal enhancements for INEPT sequences are calculated in the same way and were referenced to thermally polarised INEPT spectra recorded using the same delay times.

S1.4 Computational details

The molecular structures of **1**-**3** were optimised using DFT, the PBE0 exchange-correlation functional,[4] the empirical DFT-D3 BJ dispersion correction,[5,6]and the def2-QZVPP basis sets[7] on the Turbomole software.[8] The optimised structures can be found in Tables S2-S4. The spin-spin coupling constants were calculated on the Dalton software[9,10] using DFT/PBE0 and the polarisation-consistent pcJ-2 basis sets[11] optimised for DFT calculation of spin-spin couplings.
Coherent spin dynamics were simulated using the integrated Liouville-von Neumann equation (Equation 4) where $\hat{\rho}$ is the density operator, t is time, and $\hat{L}$ is the Liouvillian superoperator.

$$\hat{\rho}(t) = e^{\hat{L}t}\hat{\rho}(0) \quad (4)$$

The simulation used discrete time steps and a Zeeman basis. The interactions included in the simulation were the Zeeman interaction, chemical shift, and J-coupling. Both the chemical shift and the J-coupling were assumed to be isotropic. The chemical shifts, for **1**-**3,** were set to their experimental values (Table S1). The J-couplings, for **1**-**3**, were set to the DFT-calculated values (Table S5-7). For the hyperpolarised simulations, the spins were initially completely unpolarised, except for the OH-proton that was 100% polarised. For the thermal simulations, the initial spin polarisation was according to the the Boltzmann distribution (at 298 K). Details of the spectral simulations and calculations of ^{1}H enhancement factors based on them can be found in Sections S7.2 and S7.3. The coherent spin dynamics were run with Mathematica 12.3.[12] (Code available as supporting information)

S2: NMR resonances of 1-3

^{1}H and ^{13}C NMR resonances for **1**-**3** were collected at 9.4 T and 298 K for concentrated samples (0.5 mL) in dichloromethane-d_2 (0.1 mL) for reference. These values are given in Table S1.

Table S1. ^{1}H and ^{13}C NMR chemical shifts for **1**-**3** (0.5 mL) in dichloromethane-d_2 (0.1 mL) recorded at 9.4 T and 298 K.

Sites	Substrate		
	**1**	**2**	**3**
H_a	1.38	1.41	1.40
H_b	4.28	4.25	4.26
OH	3.42	2.76	3.15
H_d	3.75	4.25	4.18
H_e	N/A	1.31	1.66
H_f	N/A	N/A	1.41
H_g	N/A	N/A	0.96
C_a	20.13	20.22	20.17
C_b	66.79	66.71	66.70
C_c	176.00	175.67	175.74
C_d	52.22	61.57	62.25
C_e	N/A	13.94	30.56
C_f	N/A	N/A	18.99
C_g	N/A	N/A	13.36

S3: ^{1}H NMR signal enhancements of 1-3

When SABRE-Relay experiments are performed, enhanced ^{1}H NMR signals for **1**, **2** or **3** can each be observed. Example hyperpolarised ^{1}H NMR spectra for **1**-**3** using the carriers NH_3 or BnNH$_2$-d_7 are shown in Figures S2-7.

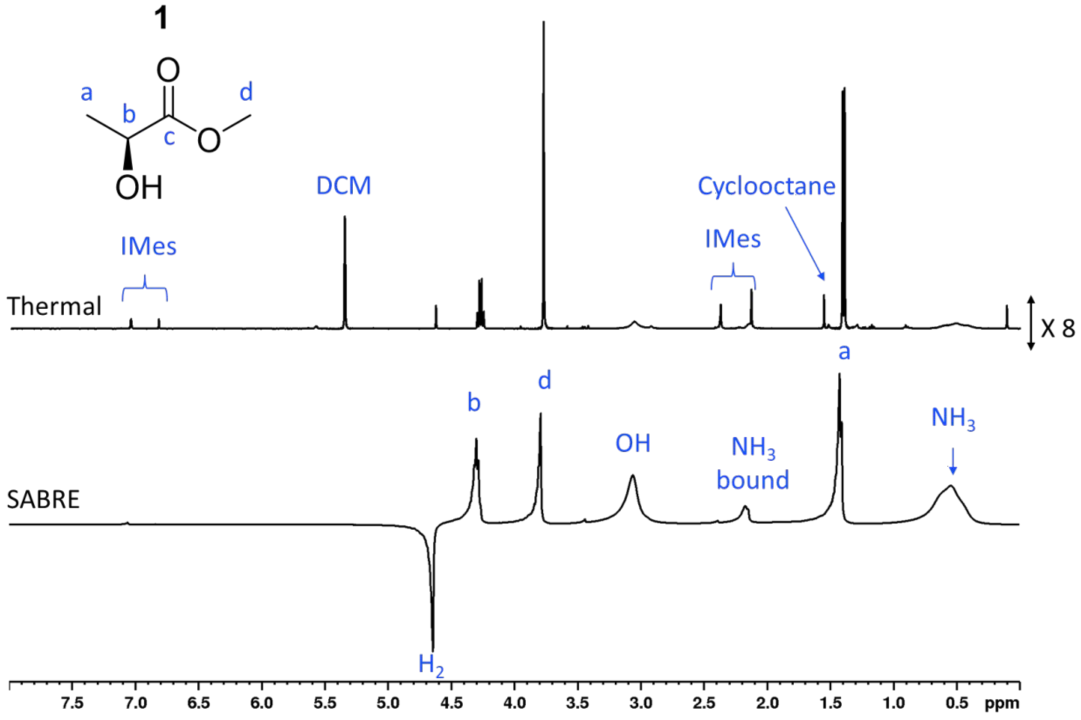

Figure S2. Partial single-scan ^{1}H NMR spectra recorded at 9.4 T and 298 K after a sample containing [IrCl(COD)(IMes)] (5 mM), NH_3 (8 equiv.) and **1** (5 equiv.) in 0.6 mL dichloromethane-d_2 is shaken with 3-bar pH$_2$ for 10 seconds at 6.5 mT. The corresponding single-scan thermal trace (expanded vertically by a factor of 8) is shown above.

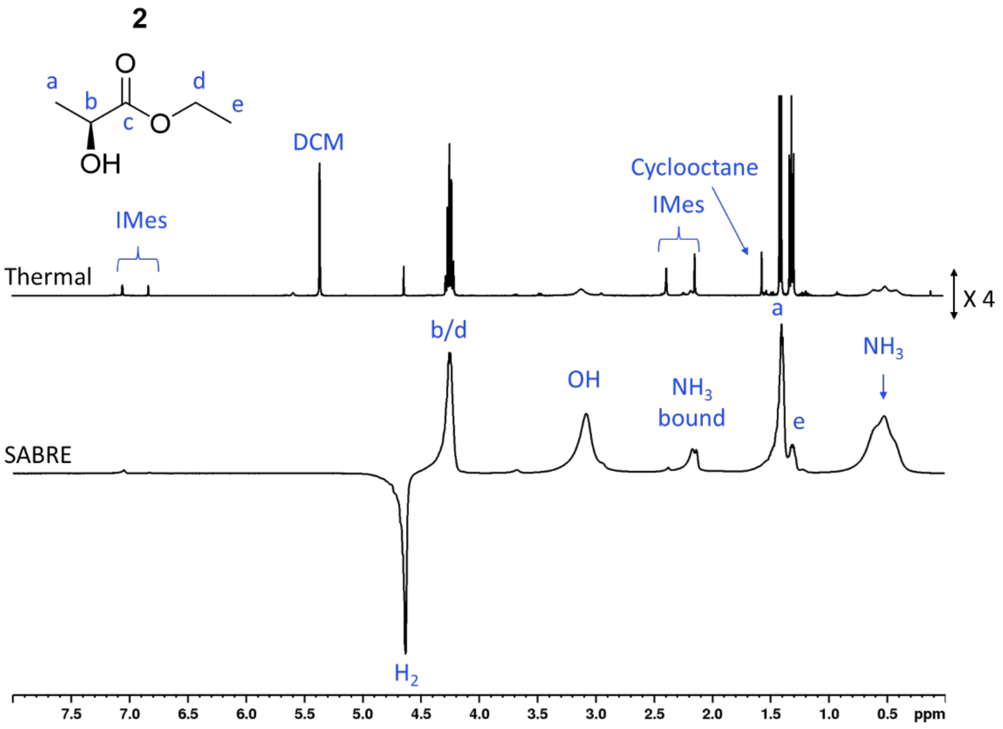

Figure S3. Partial single-scan ^{1}H NMR spectra recorded at 9.4 T and 298 K after a sample containing [IrCl(COD)(IMes)] (5 mM), NH_3 (9 equiv.) and **2** (5 equiv.) in 0.6 mL dichloromethane-d_2 is shaken with 3-bar pH$_2$ for 10 seconds at 6.5 mT. The corresponding single-scan thermal trace (expanded vertically by a factor of 4) is shown above.

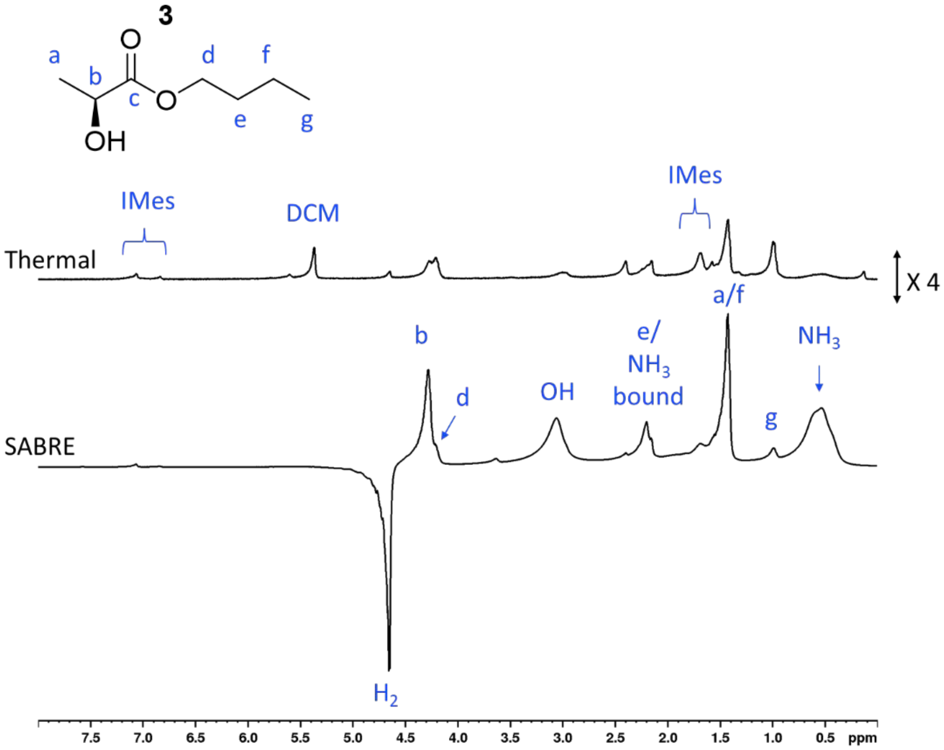

Figure S4. Partial single-scan ^{1}H NMR spectra recorded at 9.4 T and 298 K after a sample containing [IrCl(COD)(IMes)] (5 mM), NH_3 (12 equiv.) and **3** (5 equiv.) in 0.6 mL dichloromethane-d_2 is shaken with 3-bar pH$_2$ for 10 seconds at 6.5 mT. The corresponding single-scan thermal trace (expanded vertically by a factor of 4) is shown above.

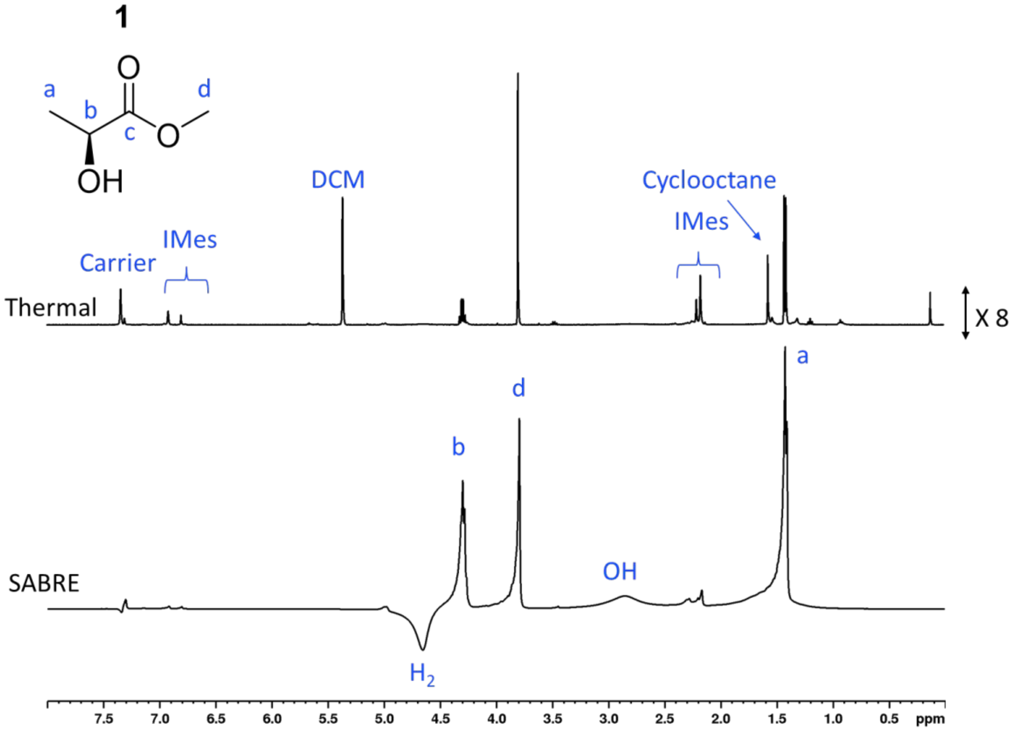

Figure S5. Partial single-scan ^{1}H NMR spectra recorded at 9.4 T and 298 K after a sample containing [IrCl(COD)(IMes)] (5 mM), $BnNH_2$-d_7 (5 equiv.) and **1** (5 equiv.) in 0.6 mL dichloromethane-d_2 is shaken with 3-bar $p$$H_2$ for 10 seconds at 6.5 mT. The corresponding single-scan thermal trace (expanded vertically by a factor of 8) is shown above.

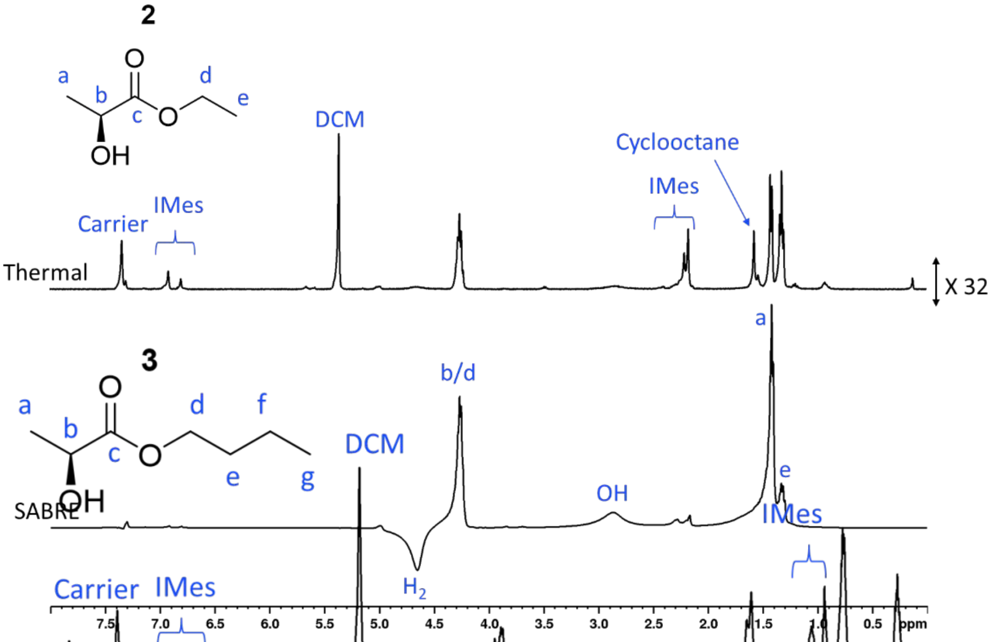

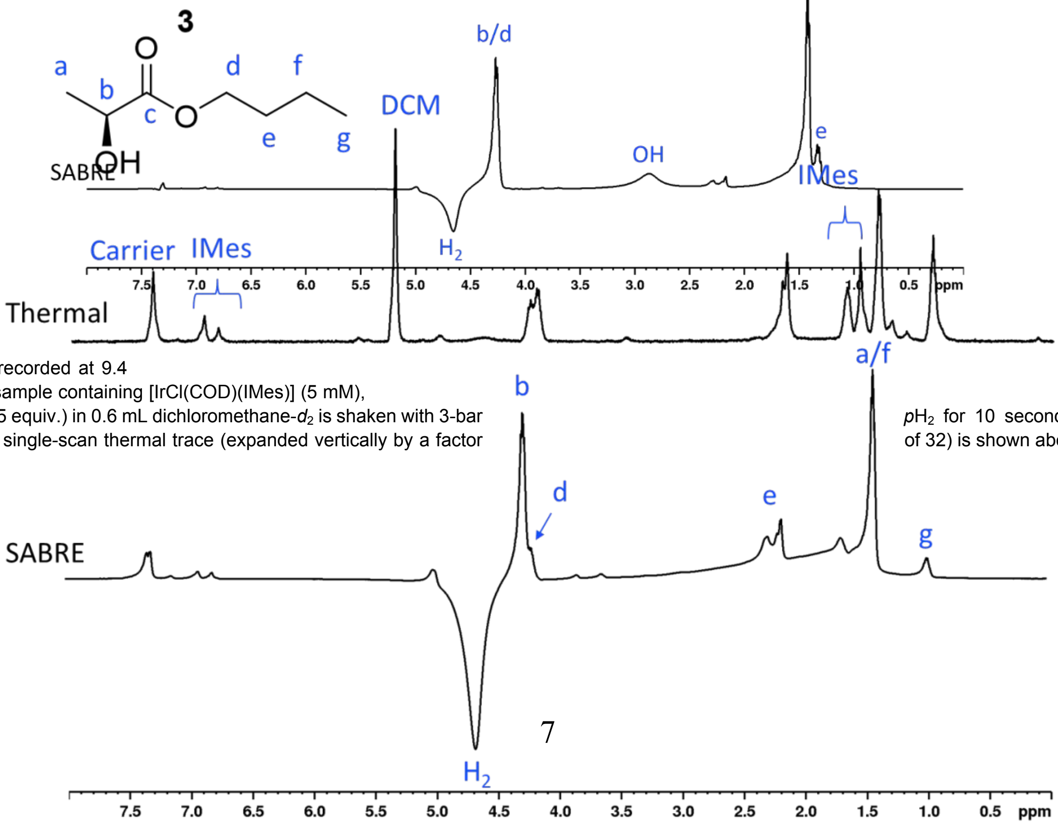

Figure S6. Partial single-scan ^{1}H NMR spectra recorded at 9.4 T and 298 K after a sample containing [IrCl(COD)(IMes)] (5 mM), $BnNH_2$-d_7 (5 equiv.) and **2** (5 equiv.) in 0.6 mL dichloromethane-d_2 is shaken with 3-bar $p$$H_2$ for 10 seconds at 6.5 mT. The corresponding single-scan thermal trace (expanded vertically by a factor of 32) is shown above.

Figure S7. Partial single-scan ^{1}H NMR spectra recorded at 9.4 T and 298 K after a sample containing [IrCl(COD)(IMes)] (5 mM), $BnNH_2$-d_7 (5 equiv.) and **3** (5 equiv.) in 0.6 mL dichloromethane-d_2 is shaken with 3-bar pH_2 for 10 seconds at 6.5 mT. The corresponding single-scan thermal trace (expanded vertically by a factor of 32) is shown above.

S4: ^{13}C NMR signal enhancements of 1-3

S4.1 Single 90° pulse ^{13}C NMR detection

When SABRE-Relay experiments are performed, enhanced ^{13}C NMR signals for **1**, **2** or **3** can each be observed. Example hyperpolarised ^{13}C NMR spectra for **1-3** using the carriers NH_3 or $BnNH_2$-d_7 are shown in Figures S8-10.

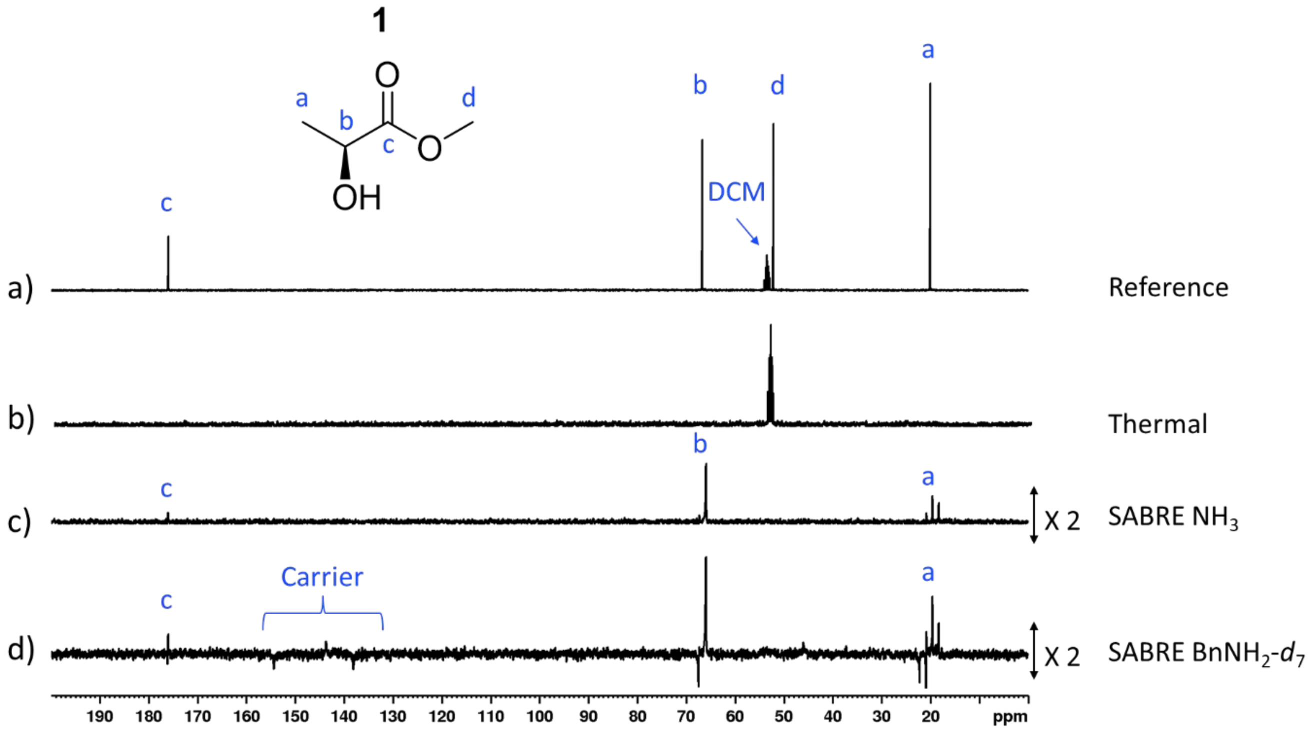

Figure S8. a) Reference ^{13}C NMR spectrum of **1** (0.5 mL) in dichloromethane-d_2 (0.1 mL) (128 scans, not to scale); b) thermally polarised single-scan ^{13}C NMR spectrum of [IrCl(COD)(IMes)] (5 mM), NH_3 (8 equiv.) and **1** (5 equiv.) with 3-bar pH$_2$ in dichloromethane-d_2 (0.6 mL); c) hyperpolarised single-scan ^{13}C NMR spectrum when the sample from b) is shaken with fresh 3-bar pH$_2$ for 10 seconds at 6.5 mT; d) SABRE-Relay hyperpolarised ^{13}C NMR spectrum of [IrCl(COD)(IMes)] (5 mM), $BnNH_2$-d_7 (5 equiv.) and **1** (5 equiv.) with 3-bar pH$_2$ in dichloromethane-d_2 (0.6 mL) shaken with fresh 3-bar pH$_2$ for 10 seconds at 6.5 mT. All spectra are recorded at 9.4 T and 298 K.

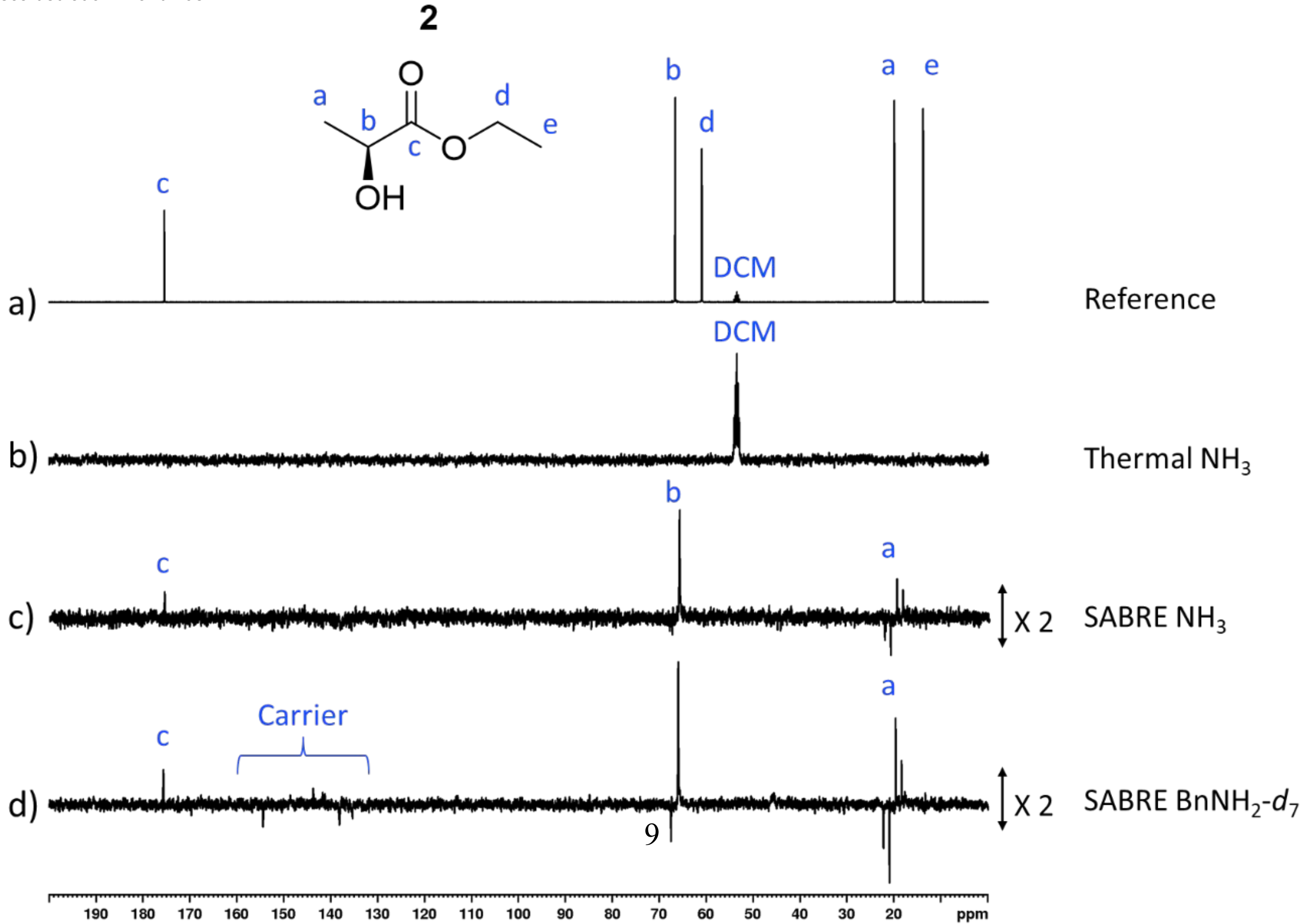

Figure S9. a) Reference ^{13}C NMR spectrum of **2** (0.5 mL) in dichloromethane-d_2 (0.1 mL) (128 scans, not to scale); b) thermally polarised single-scan ^{13}C NMR spectrum of [IrCl(COD)(IMes)] (5 mM), NH_3 (9 equiv.) and **2** (5 equiv.) with 3-bar pH_2 in dichloromethane-d_2 (0.6 mL); c) hyperpolarised single-scan ^{13}C NMR spectrum when the sample from b) is shaken with fresh 3-bar pH_2 for 10 seconds at 6.5 mT; d) SABRE-Relay hyperpolarised ^{13}C NMR spectrum of [IrCl(COD)(IMes)] (5 mM), $BnNH_2$-d_7 (5 equiv.) and **2** (5 equiv.) with 3-bar pH_2 in dichloromethane-d_2 (0.6 mL) shaken with fresh 3-bar pH_2 for 10 seconds at 6.5 mT. All spectra are recorded at 9.4 T and 298 K.

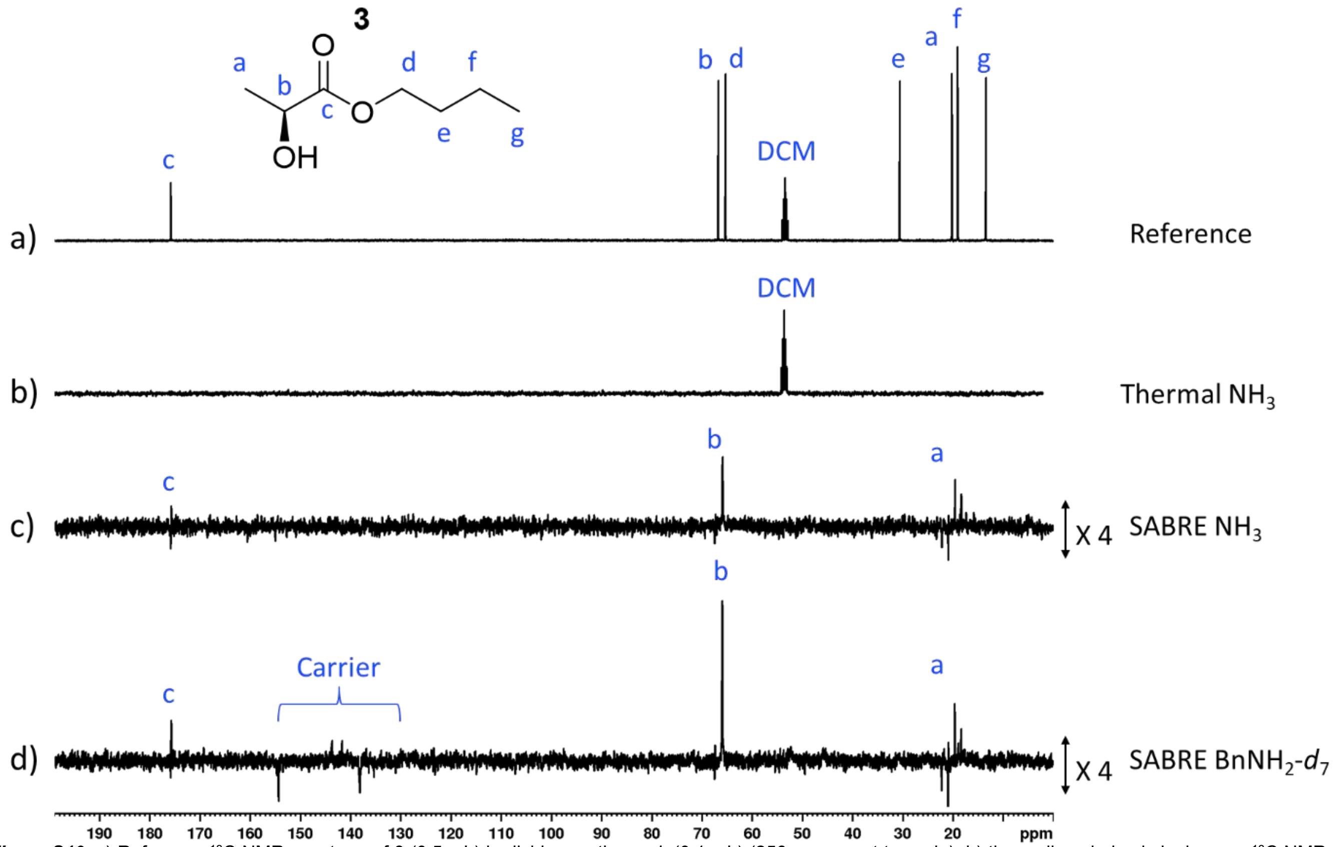

Figure S10. a) Reference ^{13}C NMR spectrum of **3** (0.5 mL) in dichloromethane-d_2 (0.1 mL) (256 scans, not to scale); b) thermally polarised single-scan ^{13}C NMR spectrum of [IrCl(COD)(IMes)] (5 mM), NH_3 (12 equiv.) and **3** (5 equiv.) with 3-bar $p$$H_2$ in dichloromethane-d_2 (0.6 mL); c) hyperpolarised single-scan ^{13}C NMR spectrum when the sample from b) is shaken with fresh 3-bar $p$$H_2$ for 10 seconds at 6.5 mT; d) SABRE-Relay hyperpolarised ^{13}C NMR spectrum of [IrCl(COD)(IMes)] (5 mM), $BnNH_2$-d_7 (5 equiv.) and **3** (5 equiv.) with 3-bar $p$$H_2$ in dichloromethane-d_2 (0.6 mL) shaken with fresh 3-bar $p$$H_2$ for 10 seconds at 6.5 mT. All spectra are recorded at 9.4 T and 298 K.

S4.2 ^{1}H → ^{13}C INEPT short-range (125 Hz)

^{1}H → ^{13}C INEPT (Insensitive Nuclei Enhanced by Polarisation Transfer) pulse sequences were used to transfer SABRE-Relay derived ^{1}H polarisation to ^{13}C sites in **1**-**3** to examine if they could give greater ^{13}C NMR signal enhancement, particularly on the carbonyl site. These sequences contain a variable time delay that is related to the size of the *J*-coupling connecting the ^{1}H-^{13}C spin pair between which magnetisation is transferred. Sequences were used with a time delay optimised for polarisation transfer between ^{1}H-^{13}C spin pairs with a short-range 125 Hz coupling. The effect of these ^{1}H→^{13}C INEPT sequences following hyperpolarisation of **1**-**3** using SABRE-Relay is shown in Figures S11-13.

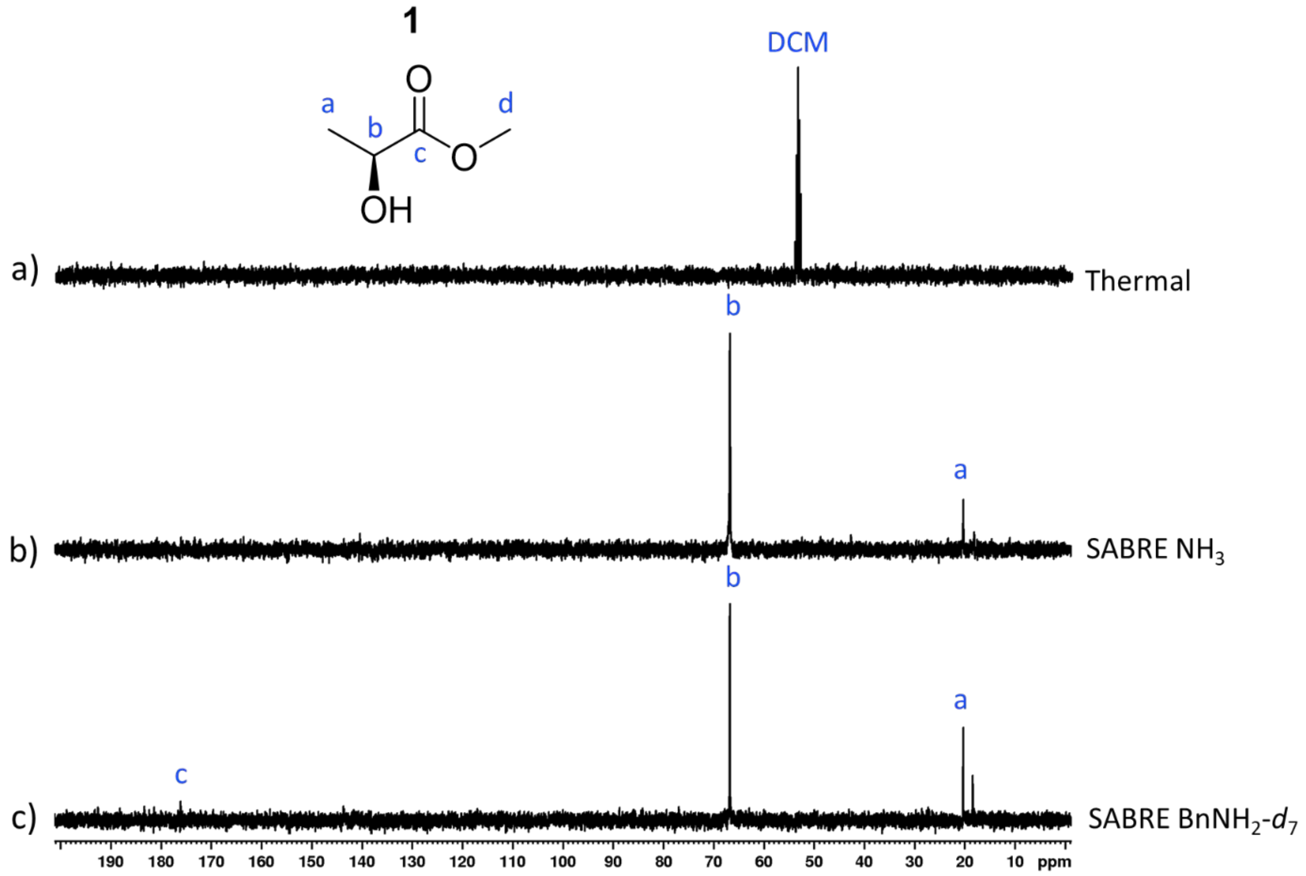

Figure S11. a) Thermally polarised single-scan ^{1}H → ^{13}C INEPT NMR spectrum of [IrCl(COD)(IMes)] (5 mM), NH_3 (8 equiv.) and **1** (5 equiv.) with 3-bar *p*H_2 in dichloromethane-d_2 (0.6 mL); b) hyperpolarised single-scan ^{13}C NMR spectrum when the sample from a) is shaken with fresh 3-bar *p*H_2 for 10 seconds at 6.5 mT; c) SABRE-Relay hyperpolarised ^{1}H → ^{13}C INEPT NMR spectrum of [IrCl(COD)(IMes)] (5 mM), BnNH$_2$-d_7 (5 equiv.) and **1** (5 equiv.) with 3-bar *p*H_2 in dichloromethane-d_2 (0.6 mL) shaken with fresh 3-bar *p*H_2 for 10 seconds at 6.5 mT. All spectra are recorded at 9.4 T and 298 K.

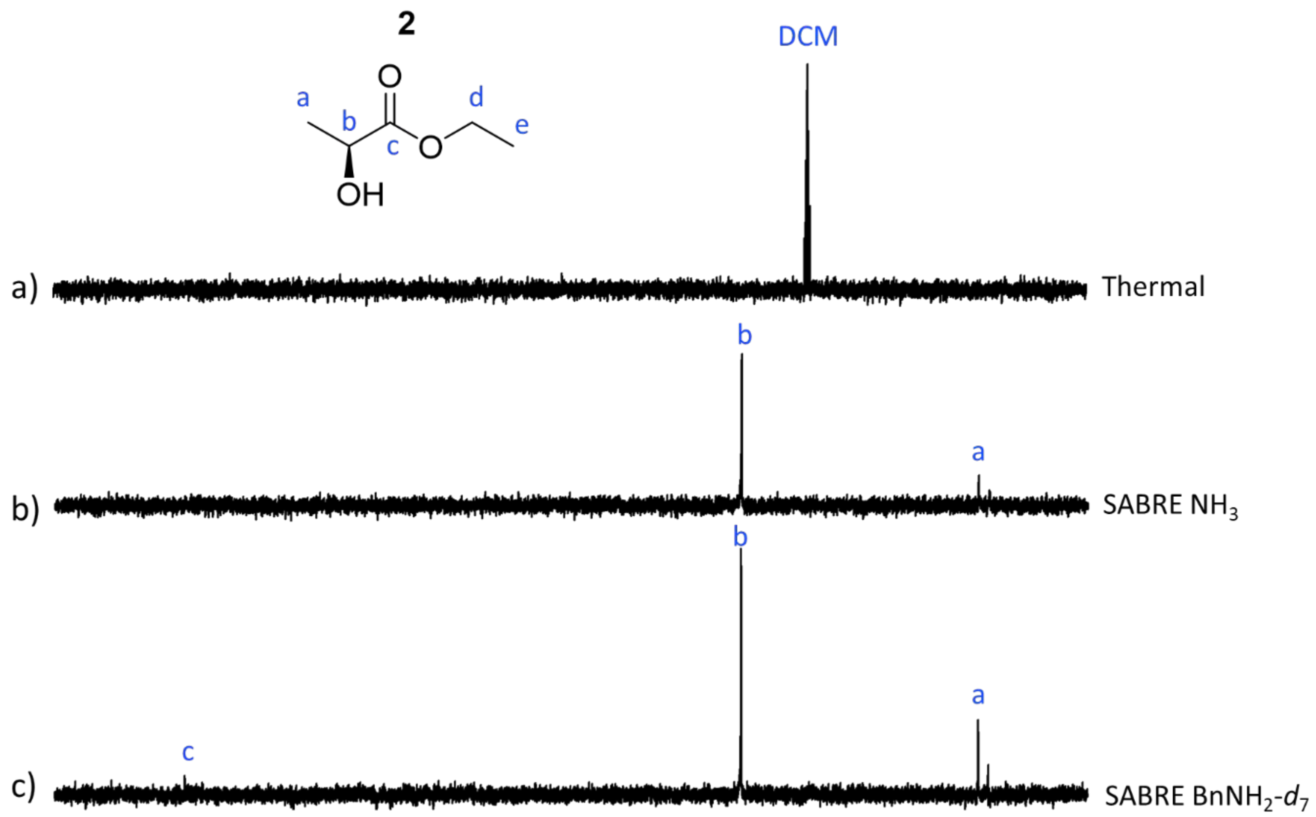

Figure S12. a) Thermally polarised single-scan ^{1}H → ^{13}C INEPT NMR spectrum of [IrCl(COD)(IMes)] (5 mM), NH_3 (9 equiv.) and **2** (5 equiv.) with 3-bar pH_2 in dichloromethane-d_2 (0.6 mL) b) hyperpolarised single-scan ^{13}C NMR spectrum when the sample from a) is shaken with fresh 3-bar pH_2 for 10 seconds at 6.5 mT c) SABRE-Relay hyperpolarised ^{1}H → ^{13}C INEPT NMR spectrum of [IrCl(COD)(IMes)] (5 mM), $BnNH_2$-d_7 (5 equiv.) and **2** (5 equiv.) with 3-bar pH_2 in dichloromethane-d_2 (0.6 mL) shaken with fresh 3-bar pH_2 for 10 seconds at 6.5 mT. All spectra are recorded at 9.4 T and 298 K.

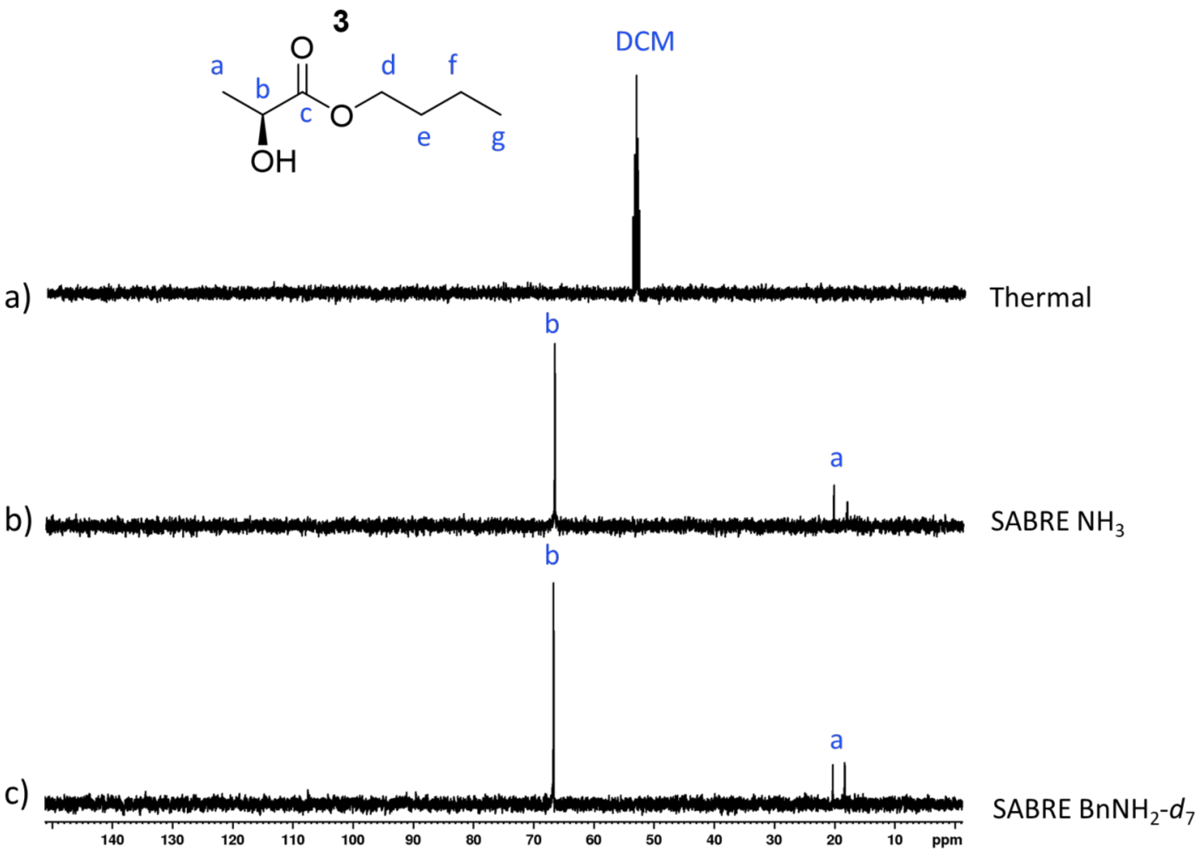

Figure S13. a) Thermally polarised single-scan $^{1}H \rightarrow {}^{13}C$ INEPT NMR spectrum of [IrCl(COD)(IMes)] (5 mM), NH_3 (12 equiv.) and **3** (5 equiv.) with 3-bar pH_2 in dichloromethane-d_2 (0.6 mL); b) hyperpolarised single-scan ^{13}C NMR spectrum when the sample from a) is shaken with fresh 3-bar pH_2 for 10 seconds at 6.5 mT; c) SABRE-Relay hyperpolarised $^{1}H \rightarrow {}^{13}C$ INEPT NMR spectrum of [IrCl(COD)(IMes)] (5 mM), $BnNH_2$-d_7 (5 equiv.) and **3** (5 equiv.) with 3-bar pH_2 in dichloromethane-d_2 (0.6 mL) shaken with fresh 3-bar pH_2 for 10 seconds at 6.5 mT. All spectra are recorded at 9.4 T and 298 K.

S4.3 ^1H → ^{13}C INEPT short-range (10 Hz)

^1H → ^{13}C INEPT (Insensitive Nuclei Enhanced by Polarisation Transfer) pulse sequences optimised for polarisation transfer between ^1H-^{13}C spin pairs with a long-range 10 Hz coupling were also used to transfer SABRE-Relay derived ^1H polarisation to ^{13}C sites in **1**-**3**. The effect of these ^1H→^{13}C INEPT sequences following hyperpolarisation of **1**-**3** using SABRE-Relay is shown in Figures S14-16.

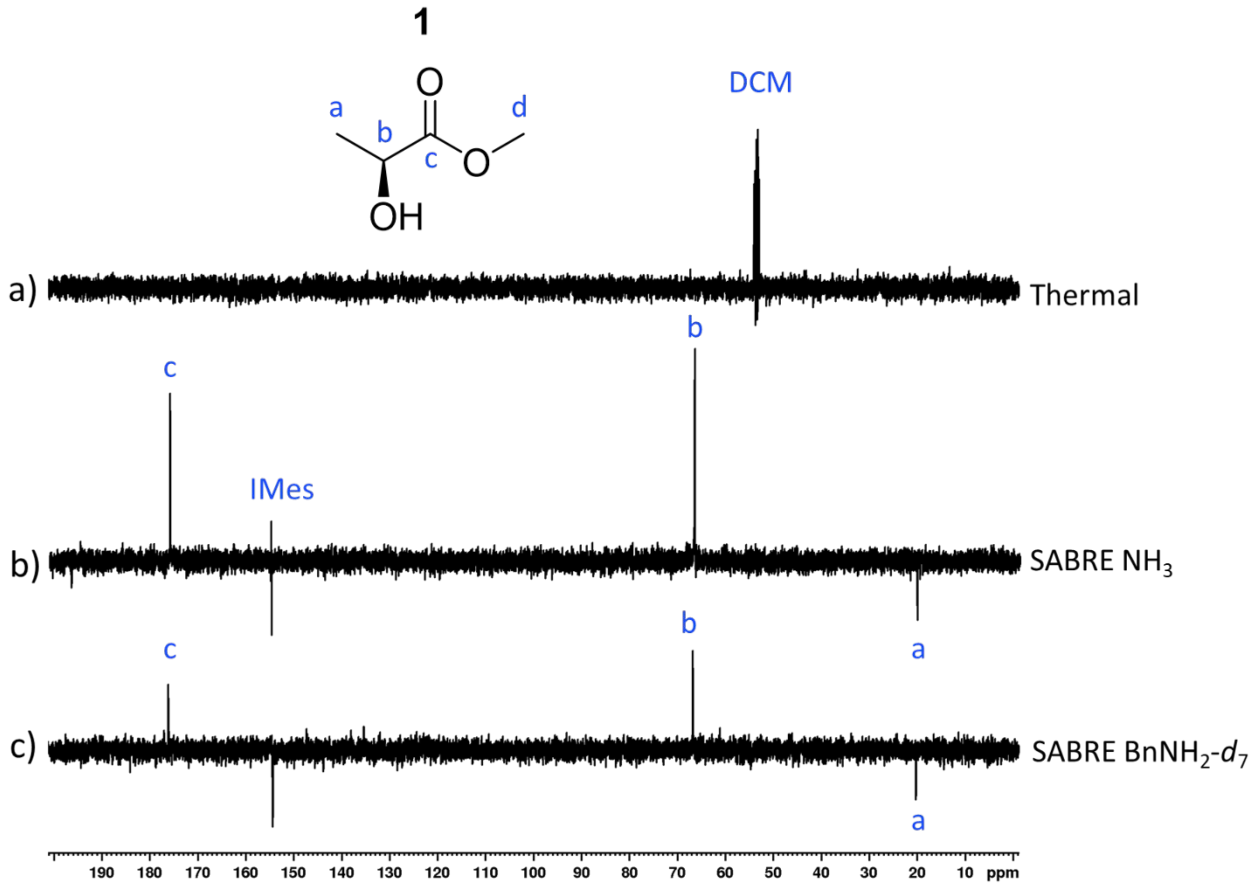

Figure S14. a) Thermally polarised single-scan ^1H → ^{13}C INEPT NMR spectrum of [IrCl(COD)(IMes)] (5 mM), NH_3 (8 equiv.) and **1** (5 equiv.) with 3-bar *p*H_2 in dichloromethane-*d*$_2$ (0.6 mL); b) hyperpolarised single-scan ^{13}C NMR spectrum when the sample from a) is shaken with fresh 3-bar *p*H_2 for 10 seconds at 6.5 mT; c) SABRE-Relay hyperpolarised ^1H → ^{13}C INEPT NMR spectrum of [IrCl(COD)(IMes)] (5 mM), $BnNH_2$-*d*$_7$ (5 equiv.) and **1** (5 equiv.) with 3-bar *p*H_2 in dichloromethane-*d*$_2$ (0.6 mL) shaken with fresh 3-bar *p*H_2 for 10 seconds at 6.5 mT. All spectra are recorded at 9.4 T and 298 K. The enhanced resonance labelled 'IMes' refers to the carbene carbon of the IMes ligand within $[Ir(H)_2(IMes)(NH_3)_3]Cl$.[2,3]

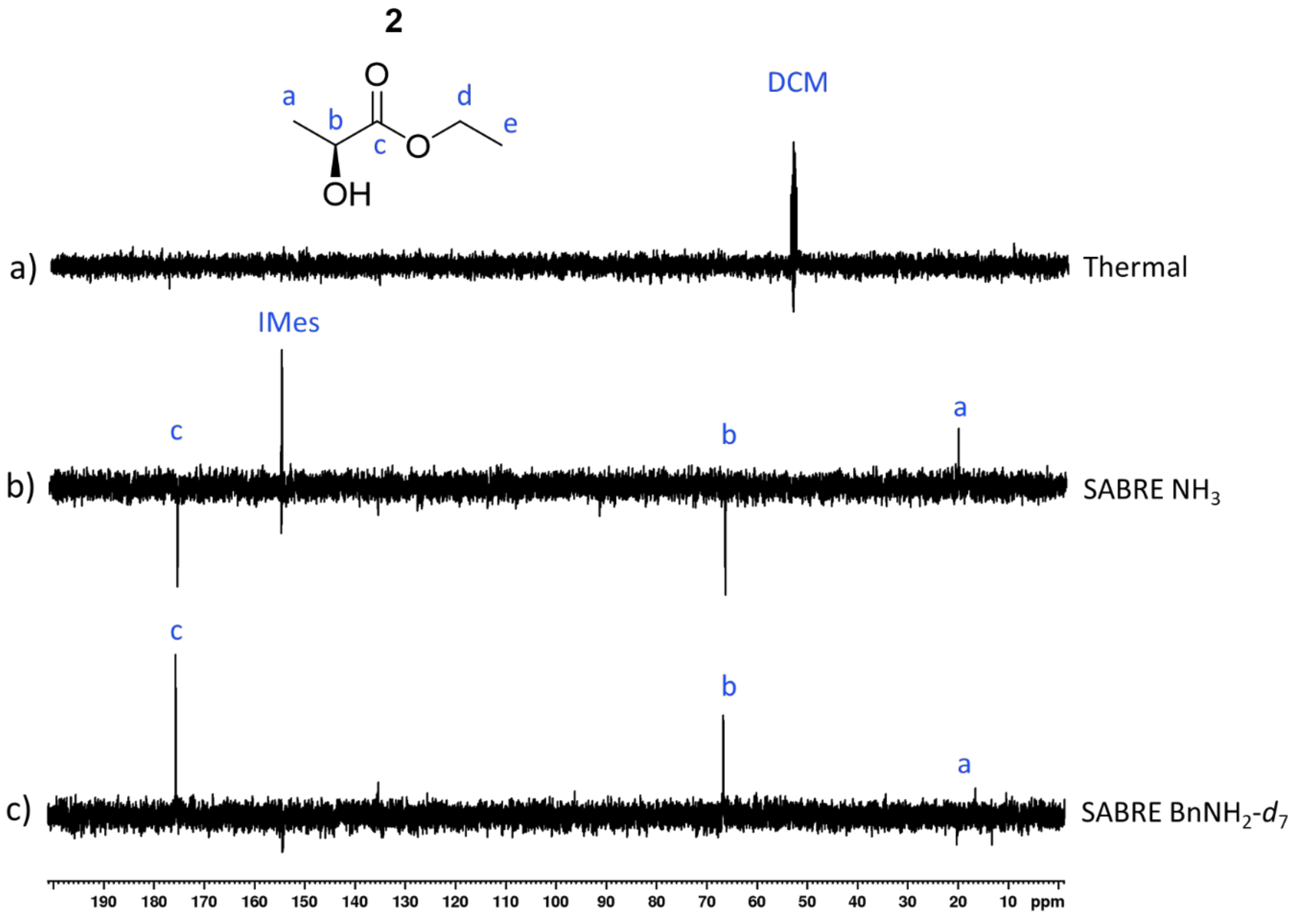

Figure S15. a) Thermally polarised single-scan ^{1}H → ^{13}C INEPT NMR spectrum of [IrCl(COD)(IMes)] (5 mM), NH_3 (9 equiv.) and **2** (5 equiv.) with 3-bar pH_2 in dichloromethane-d_2 (0.6 mL); b) hyperpolarised single-scan ^{13}C NMR spectrum when the sample from a) is shaken with fresh 3-bar pH_2 for 10 seconds at 6.5 mT; c) SABRE-Relay hyperpolarised ^{1}H → ^{13}C INEPT NMR spectrum of [IrCl(COD)(IMes)] (5 mM), $BnNH_2$-d_7 (5 equiv.) and **2** (5 equiv.) with 3-bar pH_2 in dichloromethane-d_2 (0.6 mL) shaken with fresh 3-bar pH_2 for 10 seconds at 6.5 mT. All spectra are recorded at 9.4 T and 298 K. The enhanced resonance labelled 'IMes' refers to the carbene carbon of the IMes ligand within $[Ir(H)_2(IMes)(NH_3)_3]Cl$.[2,3]

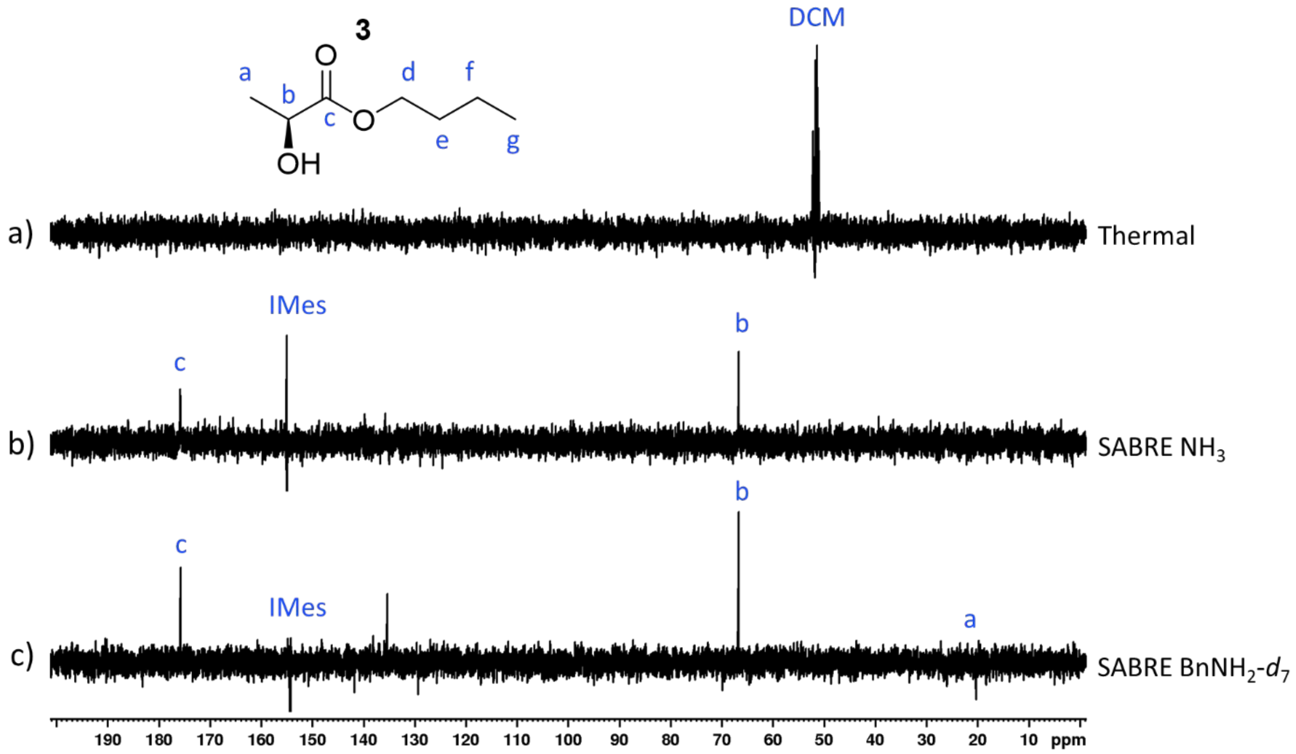

***Figure S16**.* a) Thermally polarised single-scan ^{1}H → ^{13}C INEPT NMR spectrum of [IrCl(COD)(IMes)] (5 mM), NH_3 (12 equiv.) and **3** (5 equiv.) with 3-bar pH_2 in dichloromethane-d_2 (0.6 mL); b) hyperpolarised single-scan ^{13}C NMR spectrum when the sample from a) is shaken with fresh 3-bar pH_2 for 10 seconds at 6.5 mT; c) SABRE-Relay hyperpolarised ^{1}H → ^{13}C INEPT NMR spectrum of [IrCl(COD)(IMes)] (5 mM), $BnNH_2$-d_7 (5 equiv.) and **3** (5 equiv.) with 3-bar pH_2 in dichloromethane-d_2 (0.6 mL) shaken with fresh 3-bar pH_2 for 10 seconds at 6.5 mT. All spectra are recorded at 9.4 T and 298 K. The enhanced resonance labelled 'IMes' refers to the carbene carbon of the IMes ligand within $[Ir(H)_2(IMes)(NH_3)_3]Cl$.[2,3]

S5: Hyperpolarised ^{13}C T_1 determination

Estimates of hyperpolarised ^{13}C magnetisation lifetimes were measured at 9.4 T by leaving the hyperpolarised sample (with carrier and catalyst) in the 9.4 T magnet for a varying delay time before recording a single-scan ^{13}C NMR spectrum with 90° pulse. An example decay of hyperpolarised ^{13}C NMR signals as a function of this delay time is shown in Figure S17. These signal decay curves were used to estimate T_1 times by fitting the data to an exponential signal decay function. This yielded T_1 values of *ca.* <5 s, ~20 s and ~50 s for the CH_3, CH and CO sites, respectively.

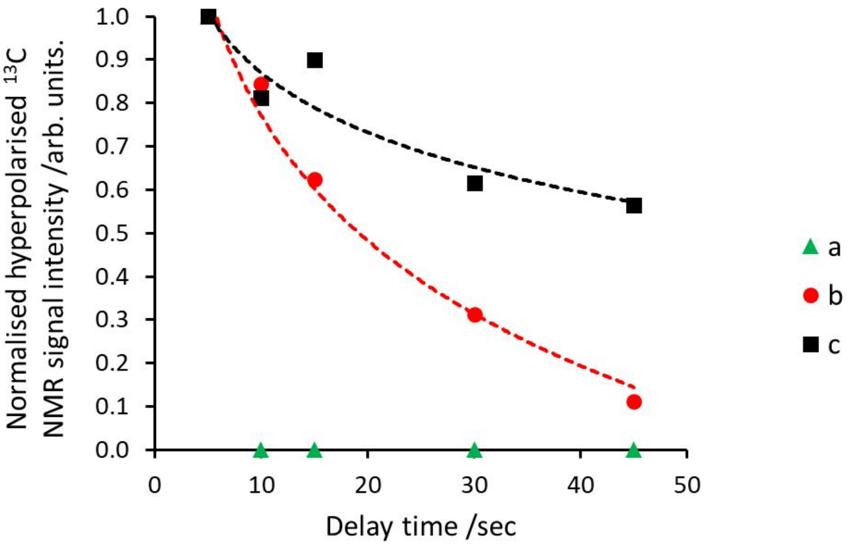

Figure S17. a) Example hyperpolarised ^{13}C NMR signal decay as a function of delay time when a sample of [IrCl(COD)(IMes)] (5 mM), NH_3 (8.5 equiv.) and **3** (5 equiv.) with 3-bar pH_2 in dichloromethane-d_2 (0.6 mL) is hyperpolarised using SABRE-Relay by shaking with fresh 3-bar pH_2 for 10 seconds at 6.5 mT before being placed rapidly into a 9.4 T spectrometer at 298 K for a variable delay time before single-scan ^{13}C NMR spectra were acquired. Fresh pH_2 shaking is performed for each data point.

S6: DFT calculation of optimised geometries and *J*-coupling constants of 1-3

Density functional theory (DFT) calculations were performed to computationally optimise the geometries of **1**-**3** (Tables S2-S4). Furthermore, *J*-couplings within **1**-**3** that might be involved in polarisation propagation from OH to other sites within **1**-**3** were also calculated (Tables S5-S7). These calculations can provide the long-range *J*-coupling values that are not discerned in ^{13}C or ^{1}H NMR spectra.

Table S2. Optimised geometry of **1** in the .xyz format (in Å).

15

H	-2.0659787	1.4325956	-0.6306670
H	-3.0552699	0.5437767	0.5467239
H	-1.7367699	1.5818950	1.1053146
H	-1.4437494	-0.9469997	-0.5697152
H	-0.2905090	-1.4250440	1.5631188
H	2.7717825	0.8683814	0.2615985
H	2.7313954	-0.4185908	-0.9558230
H	3.2121125	-0.8013907	0.7239109
C	-2.0520279	0.8985381	0.3169095
C	-1.1092046	-0.2801628	0.2410455
C	0.2794833	0.1597776	-0.1877311
C	2.5718071	-0.1890103	0.0966208
O	1.2352328	-0.5036349	0.4756635
O	0.5054916	0.9637866	-1.0475670
O	-1.1248631	-0.9617188	1.4677774

Table S3. Optimised geometry of **2** in the .xyz format (in Å).

18

H	-2.1610260	1.4778651	-0.5972863
H	-3.1155865	0.5618226	0.5879262
H	-1.7791919	1.5842107	1.1315998
H	-1.5390206	-0.9002367	-0.6174546
H	-0.3039801	-1.3943545	1.4902554
H	2.6525145	0.9430177	0.1136120
H	2.6254413	-0.3749297	-1.0517324
H	4.4757502	-0.7005211	0.5994827
H	3.2827150	-1.9929216	0.7586196
H	3.3119197	-0.6643975	1.9276869
C	-2.1191924	0.9208963	0.3362377
C	-1.1811123	-0.2571139	0.2020639
C	0.1953338	0.1946312	-0.2525450
C	2.5086411	-0.1324494	0.0054872
C	3.4448935	-0.9213507	0.8767212
O	1.1676817	-0.4519803	0.3992824
O	0.3938337	0.9981273	-1.1202083
O	-1.1632309	-0.9758781	1.4074474

Table S4. Optimised geometry of **3** in the .xyz format (in Å).

24

H	-2.3947739	1.5605330	-0.5722505
H	-3.3480361	0.6190051	0.5939092
H	-1.9981560	1.6130864	1.1558886
H	-1.7956145	-0.8219481	-0.6659985
H	-0.5383415	-1.3738773	1.4207789
H	2.4156971	0.9759841	0.0643473
H	2.3809871	-0.3367172	-1.1075885
H	2.9908589	-1.9580747	0.7031779
H	3.0368009	-0.6413520	1.8627055
H	4.8215821	-0.8735562	-0.6033536
H	4.8652550	0.4458925	0.5433373
H	6.6618555	-1.2108792	1.0408074
H	5.4556886	-2.4837040	1.2195495
H	5.5057791	-1.1510209	2.3700627
C	-2.3504908	0.9762719	0.3443168
C	-1.4242085	-0.2056202	0.1678692
C	-0.0484597	0.2466589	-0.2879822
C	2.2636822	-0.0987129	-0.0484414
C	3.2089297	-0.8919504	0.8122540

C	4.6595368	-0.6244996	0.4495769
C	5.6259381	-1.4094963	1.3161386
O	0.9241974	-0.4154184	0.3469144
O	0.1480975	1.0638045	-1.1432568
O	-1.4005868	-0.9589752	1.3516999

Table S5. *J*-coupling values (in Hz) for **1** determined using DFT, experimental values (where available) are shown in blue.

Sites	H_a	H_b	OH	H_d	C_a	C_b	C_c	C_d
H_a	-14.1	7.4 (7.0)	1.0	0.0	126.3 (128.5)	-4.8 (4.5)	5.3	0.2
H_b			-0.5	0.3	-4.4	136.3 (146.0)	-4.2	-0.1
OH				0.0	7.1	-2.7	5.2	-0.1
H_d				-12.0	0.1	-0.3	3.9	142.9 (147.5)
C_a						39.2	-1.2	0.1
C_b							57.2	1.1
C_c								-2.5

Table S6. *J*-coupling values (in Hz) for **2** determined using DFT, experimental values (where available) are shown in blue.

Sites	H_a	H_b	OH	H_d	H_e	C_a	C_b	C_c	C_d	C_e
H_a	-14.0	7.4 (7.0)	0.9	0.0	0.0	126.2	-4.8	5.3	0.2	0.0
H_b			-0.4	0.5	0.0	-4.4	136.8 (147.0)	-4.4	-0.1	0.0
OH				0.0	0.0	6.6	-2.7	5.8	-0.1	0.0
H_d				-13.2	7.9 (7.0)	0.1	-0.4	1.2	143.8 (148.0)	-2.3
H_e					-14.5	0.0	0.3	0.1	-4.1	123.8 (129.0)
C_a							39.0	-1.2	0.1	0.0
C_b								56.7	1.0	-0.1
C_c									-2.3	3.8
C_d										37.8

Table S7. *J*-coupling values (in Hz) for **3** determined using DFT, experimental values (where available) are shown in blue.

Sites	H_a	H_b	OH	H_d	H_e	H_f	H_g	C_a	C_b	C_c	C_d	C_e	C_f	C_g
H_a	-14.0	7.4 (7.0)	0.9	0.0	0.0	0.0	0.0	126.2 (128.5)	-4.8	5.3	0.2	0.0	0.0	0.0
H_b			-0.4	0.5	0.0	0.0	0.0	-4.4	136.9 (144.5)	-4.5	0.0	0.0	0.0	0.0
OH				0.0	0.0	0.0	0.0	6.4	-2.7	5.9	-0.1	0.0	0.0	0.0
H_d				-12.9	9.8 (6.5)	-0.3	0.0	0.1	-0.4	1.3	142.7 (147.5)	-2.5	1.4	-0.3
H_e					-13.5	9.3 (7.5)	-0.3	0.0	0.1	-0.3	-6.8	123.8 (125.0)	-4.7	2.3
H_f						-14.4	7.9 (7.5)	0.0	0.0	-0.1	2.7	-4.1	122.0 (125.0)	-4.2
H_g							-14.3	0.0	0.0	0.1	0.3	5.9	-4.1	122.6 (125.0)
C_a									38.9	-1.3	0.1	0.0	0.0	0.0
C_b										56.7	1.0	-0.1	0.3	0.0
C_c											-2.3	3.5	0.1	0.1
C_d												38.0	3.8	7.8
C_e													32.6	-0.1
C_f														33.5

The relative coupling network calculated for 1-3 is displayed in Figure S18. The relative coupling of nuclei A and B are defined here as $|J_{AB} / (\nu_A - \nu_B)|$, where J_{AB} is the calculated coupling constant and ν_A, ν_B are the NMR frequencies at 6.5 mT field strength.

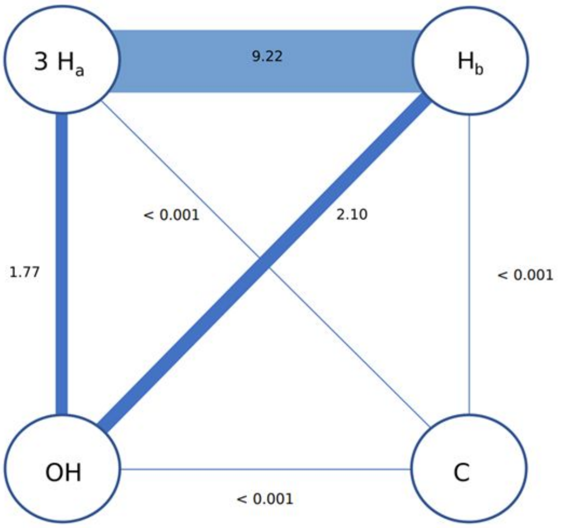

Figure S18. Relative spin-spin coupling constants $|J_{AB} / (\nu_A - \nu_B)|$ in **1**-**3** at 6.5 mT obtained using the DFT-calculated spin-spin coupling constants J_{AB} and the NMR frequencies ν_A, ν_B for nuclei A and B. C corresponds to the naturally-abundant ^{13}C site of C_a, C_b or C_c. The thickness of the lines are proportional to the relative coupling. Note that the protons in the ester side chains of **1**-**3** are omitted from this depiction.

S7: Theoretical insight into polarisation transfer

S7.1 Simulated polarisation transfer

The evolution of spin polarisation over a 10 second time window for a 5-proton system (3H$_a$, H$_b$ and OH), with and without a ^{13}C centre, at polarisation transfer fields of 0 T, 6.5 mT, and 9.4 T are shown in Figures S19, S20 and S21, respectively. These simulations use the *J* coupling values for **1** detailed in section S6. Note that similar results can be obtained for the 5 or 6-spin frameworks of **2** and **3** using the slightly different *J*-coupling values predicted for those molecules (data not shown).

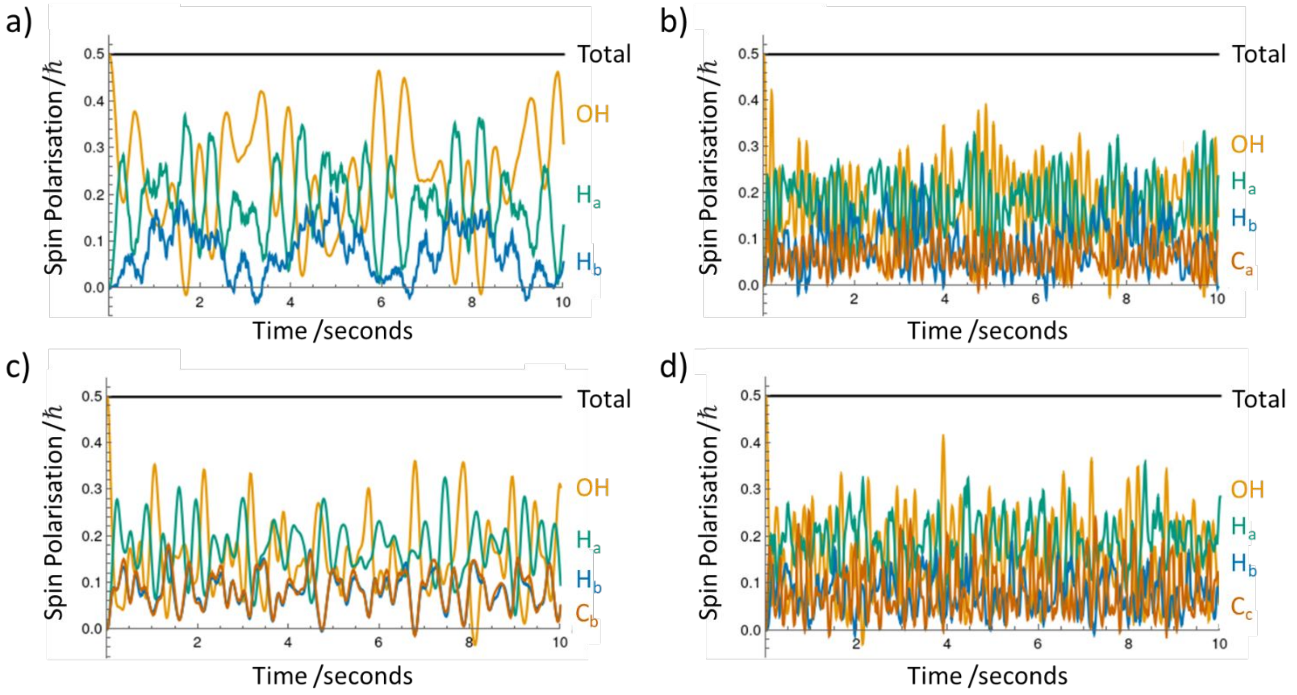

Figure S19. Spin polarisation as a function of time for the different sites a) 3H_a, H_b and OH; b) 3H_a, H_b, OH, and C_a; c) 3H_a, H_b, OH, and C_b; and d) 3H_a, H_b, OH, and C_c of **1** at a polarisation transfer field of 0 T.

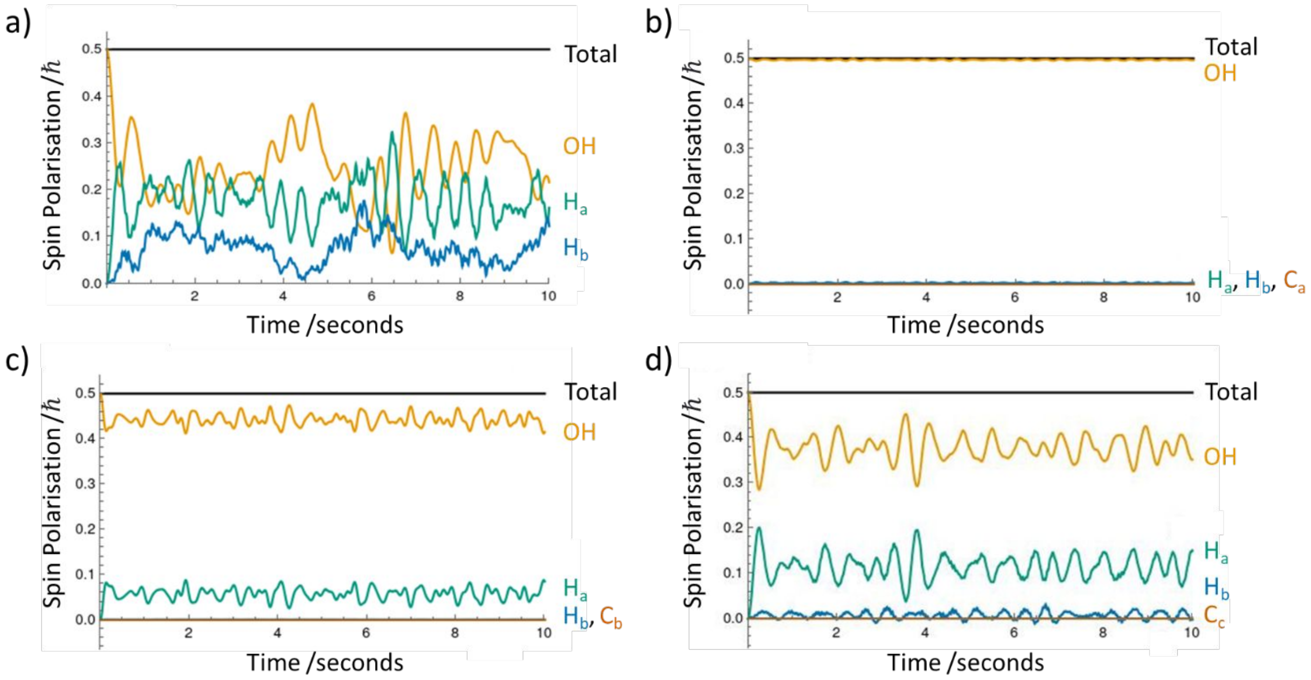

Figure S20. Spin polarisation as a function of time for the different sites a) 3H_a, H_b and OH; b) 3H_a, H_b, OH, and C_a; c) 3H_a, H_b, OH, and C_b; and d) 3H_a, H_b, OH, and C_c of **1** at a polarisation transfer field of 6.5 mT.

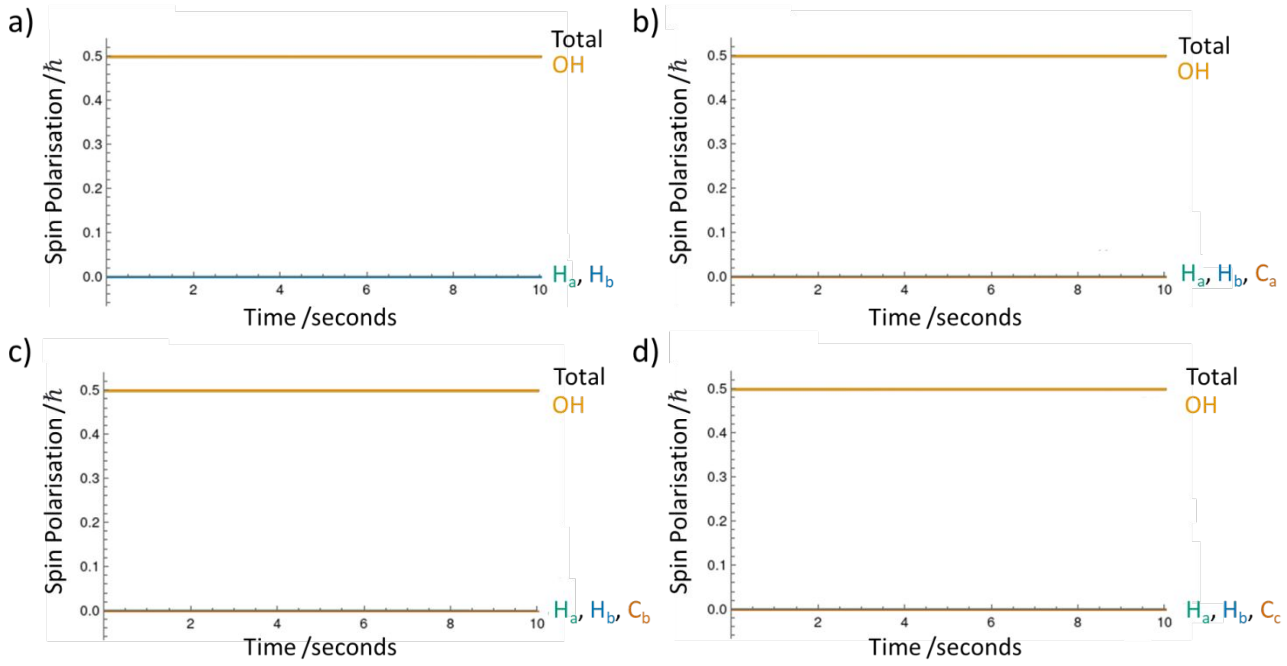

Figure S21. Spin polarisation as a function of time for the different sites a) $3H_a$, H_b and OH; b) $3H_a$, H_b, OH, and C_a; c) $3H_a$, H_b, OH, and C_b; and d) $3H_a$, H_b, OH, and C_c of **1** at a polarisation transfer field of 9.4 T.

S7.2 Simulated ^{1}H NMR spectra

Simulated ^{1}H-NMR spectra for the 5-proton system ($3H_a$, H_b and OH) of **1** for thermal and hyperpolarised initial conditions can be seen in Figures S22a and b, respectively. The same process of analysis can be performed for the 5-proton systems of **2** and **3** which involve similar J coupling propagators (data not shown) and yield similar results.

In the calculation of these spectra, coherences were removed from the initial density, in accordance with the model for coherent polarisation transfer described by Barskiy *et al.*[13] instead of running spin dynamics for the polarisation time. The removal of coherences is intended to simulate the effect of the individual lactate ester molecules exchanging protons with carrier molecules at different points in time. Details of such dynamics have been studied by Ivanov *et al*.[14] The RF pulse was approximated as a 90-degree rotation around the *x*-axis of the spin polarisation. Relaxation was implemented as an exponential function of time that was multiplied with the signal from each spin. The relaxation times were set to render the linewidths to equal 0.1 Hz. The detection time used in the simulation was 30 s, which corresponds to roughly 9-10 times the transverse relaxation time in the system. A Nyquist frequency of 3425.95 Hz (time step 1.14441×10^{-4} s) was used. In the calculation of the signal, the time derivative was performed analytically by multiplying the vectorised density matrix with the matrix form of the Liouvillian operator.

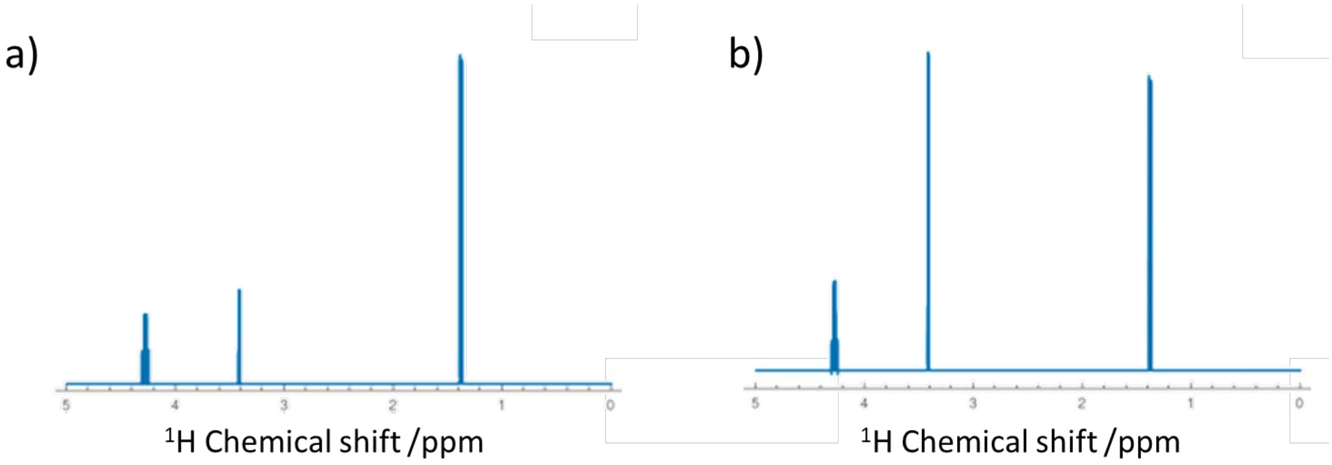

Figure S22. Simulated a) thermally polarised and b) hyperpolarised ^{1}H NMR spectra for ^{1}H-only 5 spin system ($3H_a$, H_b and H_c) of **1,** recorded at 9.4 T.

S7.3 Simulated ^1H NMR signal enhancements

Simulated ^1H spectra (one such example for **1** is shown in Figure S22) were used to calculate estimated NMR signal enhancements due only to the coherent transfer process (omitting any OH/NH exchange effects). These calculated signal enhancements are shown in Table S8 and are normalized relative to the signal intensity for the H_a proton. They are of the same order of magnitude as the experimental values and OH in all cases has the largest simulated enhancement factor which is consistent with the experimental data. This match is satisfactory with such an approximate model involving only the coherent transfer mechanism.

The NMR signal enhancements were calculated by integrating the lines in the spectra and dividing the integral of the simulated hyperpolarised signals by their thermally polarised counterpart. The lines in the spectra were integrated by fitting Lorentzian functions to them and by the trapezoidal method, out to a distance of 40 × 0.1 Hz from the outermost peaks belonging to the resonances of a certain ^1H multiplet. The simulated NMR signal enhancements were normalized relative to that of H_a to render the results comparable to the experimental values.

Table S8. Simulated ^1H NMR signal enhancement factors relative to H_a for **1**-**3**, and the corresponding experimental values.

Molecule	Spin	Simulation	Experiment	
			Carrier NH_3	Carrier $BnNH_2$-d_7
1	OH	4	2.7 ± 0.5	2.8 ± 0.3
	H_a	1	1	1
	H_b	1.3	1.8 ± 0.4	1.05 ± 0.13
2	OH	4	2.7 ± 1.1	1.22 ± 0.06
	H_a	1	1	1
	H_b	1.2	1.0 ± 0.5	0.67 ± 0.04
3	OH	4	2.5 ± 0.7	-
	H_a	1	1	1
	H_b	1.3	2.0 ± 0.6	3.5 ± 0.9

S8: Effect of NH_3 concentration on ^{1}H and ^{13}C NMR signal enhancements of 2

SABRE-Relay hyperpolarisation of **2** was tested using different amounts of the carrier NH_3. A sample containing 9 equiv. relative to the metal centre was prepared as described previously. Two other samples were also tested which contained 6.5 and 7.5 equiv NH_3. The ^{1}H and ^{13}C NMR signal enhancements for **2** in these mixtures are given in Table S9.

Table S9. ^{1}H and ^{13}C NMR signal enhancements (per site) for **2** (5 equiv.) after shaking with 3-bar *p*H_2 for 10 seconds at 6.5 mT in a solution of [IrCl(COD)(IMes)] (5 mM) pre-activated with the indicated amount of NH_3 in dichloromethane-d_2 (0.6 mL). Note that average refers to the average total substrate polarisation per site.

Amount of NH_3 /equiv. relative to Ir	Site	^{1}H NMR signal enhancement /fold	^{13}C NMR signal enhancement /fold		
			^{13}C NMR	$^{1}H\rightarrow{}^{13}C$ INEPT short-range	$^{1}H\rightarrow{}^{13}C$ INEPT long-range
6.5	a	215 ± 15	865 ± 20	35 ± 5	2 ± 1
	b	160 ± 10*	985 ± 65	110 ± 15	25 ± 5
	c	N/A	110 ± 10	0	40 ± 5
	d	160 ± 10*	0	0	0
	e	30 ± 5	0	0	0
	OH	245 ± 10	N/A	N/A	N/A
	Average	145 ± 10	685 ± 30	80 ± 10	15 ± 5
7.5	a	50 ± 5	335 ± 25		15 ± 5
	b	40 ± 5*	585 ± 40		130 ± 15
	c	N/A	80 ± 5		105 ± 10
	d	40 ± 5*	0		0
	e	10 ± 5	0		0
	OH	65 ± 5	N/A		N/A
	Average	35 ± 5	210 ± 15		45 ± 5
9.0	a	15 ± 5	640 ± 5	55 ± 5	15 ± 5
	b	15 ± 5*	420 ± 45	115 ± 15	130 ± 40
	c	N/A	85 ± 10	0	100 ± 30
	d	15 ± 5*	0	0	0
	e	2 ± 1	0	0	0
	OH	40 ± 10	N/A	N/A	N/A
	Average	15 ± 5	390 ± 20	125 ± 20	85 ± 20

*Values are averaged across the two sites due to signal overlap

S9: Effect of added H_2O on ^1H and ^{13}C NMR signal enhancements of 2

The effect of contaminant water on SABRE-Relay of **2** was investigated by doping a solution of [IrCl(COD)(IMes)] (5 mM) preactivated with NH_3 (6.5 equiv.) and **2** (5 equiv.) in anhydrous dichloromethane-d_2 (0.6 mL) with 0.5 µL H_2O. The sample was hyperpolarised using SABRE-Relay by shaking with 3-bar pH$_2$ for 10 seconds at 6.5 mT prior to and after the addition of H_2O. ^1H and ^{13}C NMR signal enhancement before and after H_2O doping are shown in Table S10 and are presented graphically in Figure S23. Example NMR spectra are shown in Figure S24.

Table S10. ^1H and ^{13}C NMR signal enhancements (per site) for **2** (5 equiv.) after shaking with 3-bar pH$_2$ for 10 seconds at 6.5 mT in a solution of [IrCl(COD)(IMes)] (5 mM) pre-activated with NH_3 (6.5 equiv.) in anhydrous dichloromethane-d_2 (0.6 mL) and after the addition of 0.5 µL H_2O. The average polarisation refers to the total hyperpolarised integral intensity of the substrate divided by its thermally polarised counterpart and average refers to the total substrate polarisation averaged per site.

Amount of added H_2O /µL	Site	^1H NMR signal enhancement /fold	^{13}C NMR signal enhancement /fold
0.0	a	215 ± 15	865 ± 20
	b	160 ± 10*	985 ± 65
	c	N/A	110 ± 10
	d	160 ± 10*	0
	e	30 ± 5	0
	OH	245 ± 10	N/A
	Average	145 ± 10	685 ± 30
0.5	a	185 ± 5	470 ± 15
	b	140 ± 5*	810 ± 30
	c	N/A	100 ± 10
	d	140 ± 5*	0
	e	30 ± 5	0
	OH	120 ± 5	N/A
	Average	115 ± 5	475 ± 15

*Values are averaged across the two sites due to signal overlap

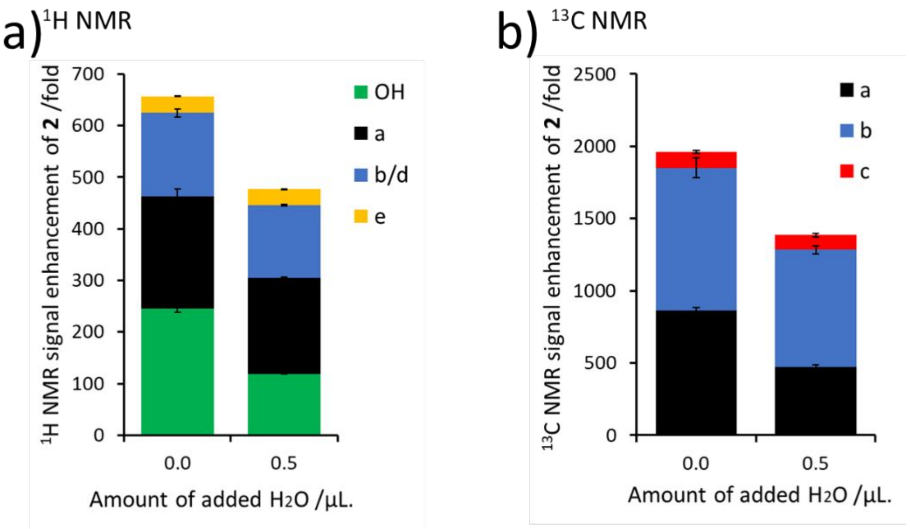

Figure S23. a) ^1H and b) ^{13}C NMR signal enhancements of **2** achieved using SABRE-Relay before and after doping a sample containing [IrCl(COD)(IMes)] (5 mM) and with NH_3 (6.5 equiv.) with 3-bar pH$_2$ in anhydrous dichloromethane-d_2 (0.6 mL) with 0.5 µL H_2O.

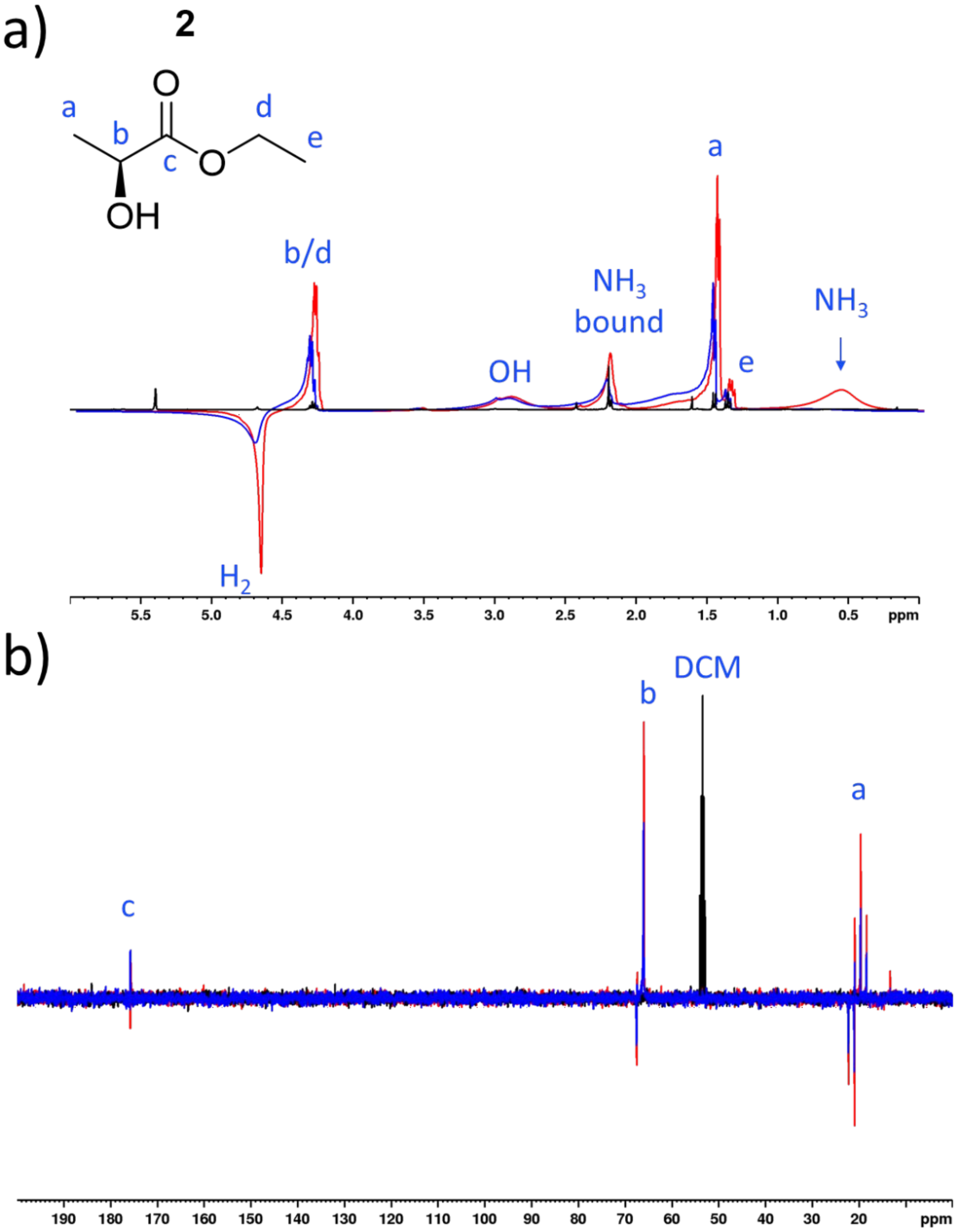

Figure S24. a) ^{1}H and b) ^{13}C NMR spectra for **2** achieved using SABRE-Relay before (red) and after (blue) doping a sample containing [IrCl(COD)(IMes)] (5 mM) and with NH_3 (6.5 equiv.) with 3-bar *p*H_2 in anhydrous dichloromethane-*d*$_2$ (0.6 mL) with 0.5 μL H_2O. A thermally polarised spectrum prior to H_2O doping is shown for comparison (black). In a) this has been expanded vertically by a factor of 8.

S10: Towards hyperpolarisation of sodium lactate

Sodium lactate (5 eq.) in H_2O (10 μL) was added to a solution of [IrCl(COD)(IMes)] (5 mM) and NH_3 (8 equiv.) preactivated overnight with 3-bar H_2. Shaking for 10 seconds with 3-bar pH_2 at 6.5 mT followed by insertion into the 9.4 T spectrometer yielded no discernible ^1H or ^{13}C signal enhancements for lactate, as shown in Figure S25.

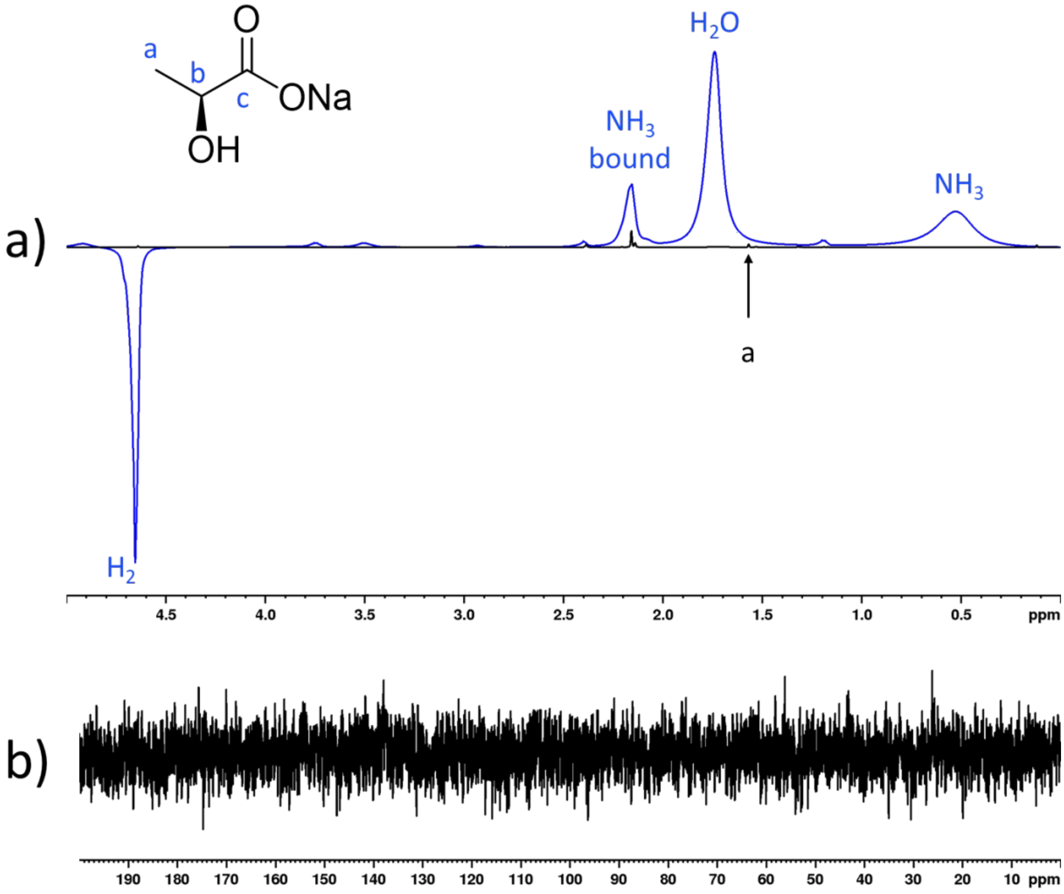

Figure S25. Single-scan a) ^1H and b) ^{13}C NMR spectra for a solution of [IrCl(COD)(IMes)] (5 mM), NH_3 (8 equiv.) and sodium lactate (5 equiv.) in dichloromethane-*d*$_2$ (0.6 mL) and 10 μL H_2O shaken with 3-bar pH_2 for 10 seconds at 6.5 mT. In a) the corresponding thermally polarised spectrum is shown in black.

The production of lactate from lactate esters could be achieved by rapid hydrolysis with NaOH (1 M) followed by HCl (1 M) in D_2O at 363 K.[15,16] We confirmed that hydrolysis of **2** could rapidly form sodium lactate by addition of NaOH followed by HCl in D_2O (total added volume of 0.6 mL, final concentrations of 1 M) at 363 K to a solution of **2** (1.8 μL) in dichloromethane-*d*$_2$ (0.6 mL). The sample was then shaken for ~2 seconds to ensure mixing before being placed back into a water bath at 363 K to allow phase separation which took a few seconds. The aqueous layer was removed *via* plastic syringe and injected into a separate NMR tube for interrogation by NMR. A comparison of ^1H NMR spectra for the aqueous layer confirmed the presence of the hydrolysis products sodium lactate and ethanol. A starting ^1H NMR spectrum of **2** (1.8 μL) in dichloromethane-*d*$_2$ (0.6 mL) was taken for comparison, as was a ^1H NMR spectrum of the dichloromethane-*d*$_2$ layer after hydrolysis which revealed the presence of residual water, some remaining **2** and hydrolysis products These spectra are shown in Figure S26.

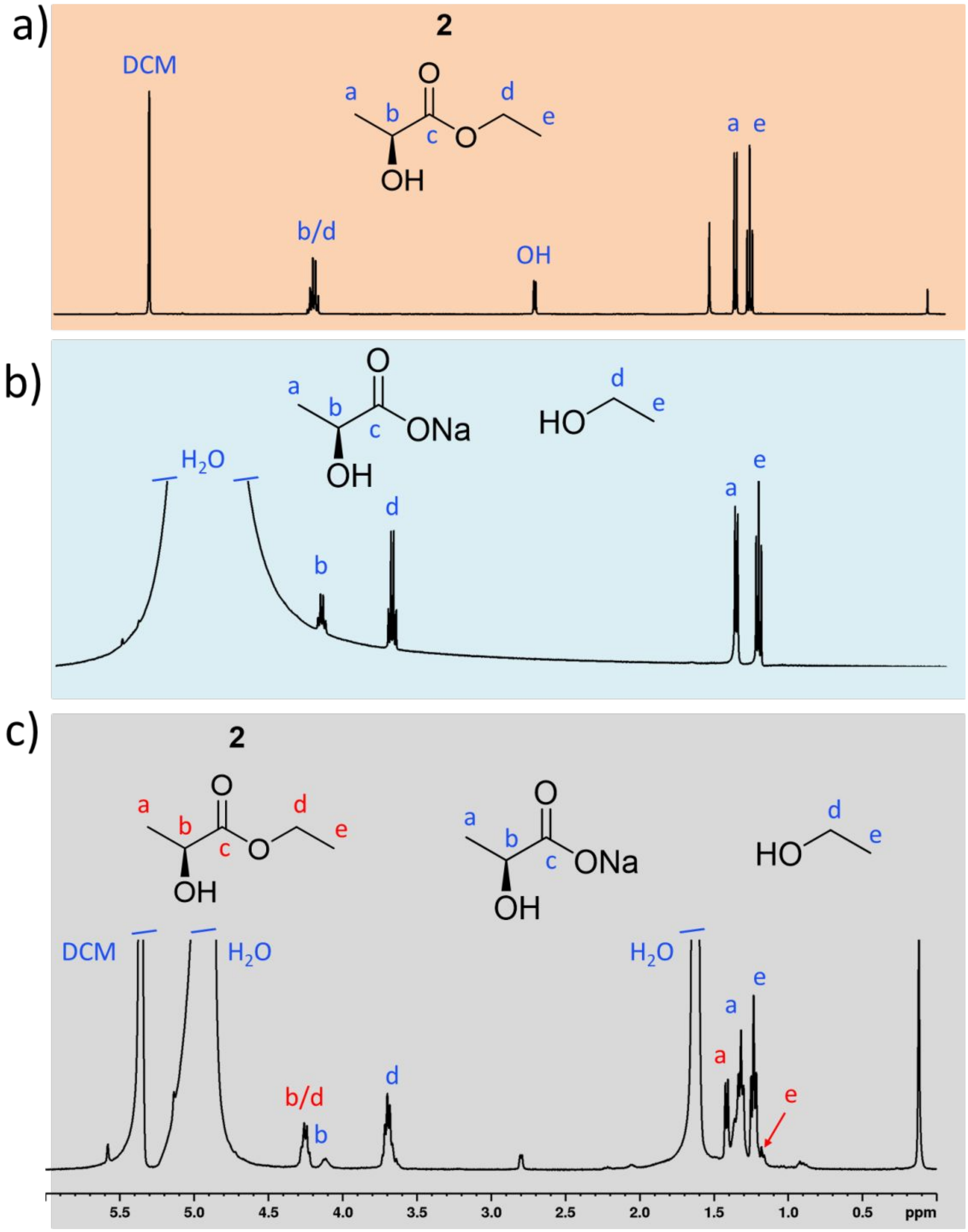

Figure S26. ^{1}H NMR spectra of a) **2** (1.8 µL) in dichloromethane-d_2 (0.6 mL); b) D_2O layer after addition of NaOH followed by HCl in D_2O (total added volume of 0.6 mL, final concentrations of 1 M) at 363 K to the solution in a); c) DCM layer after addition of NaOH followed by HCl in D_2O (total added volume of 0.6 mL, final concentrations of 1 M) at 363 K to the solution in a).

As sodium lactate could not be hyperpolarised using our SABRE-Relay experiments (Figure S25), hydrolysis of lactate esters hyperpolarised using SABRE-Relay could be a route to producing hyperpolarised lactate. Preliminary experiments were performed that involved the addition of NaOH (1 M) followed by HCl (1 M) in D_2O (0.6 mL) at 363 K to solutions of **2** hyperpolarised using SABRE-Relay. This was achieved by shaking a solution of [IrCl(COD)(IMes)] (5 mM), NH_3 (9 equiv.) and **2** (5 equiv.) with *p*H_2 for 10 seconds at 6.5 mT at room temperature before placing the NMR tube into a thermostatically controlled water bath at 363 K at Earth's field, removing the lid and injecting NaOH followed by HCl in D_2O (0.6 mL, final concentrations of 1 M). The sample was then shaken at Earth's field for ~2 seconds to ensure mixing before being placed back into the water bath to allow phase separation. The aqueous layer was removed *via* plastic syringe and injected into a separate NMR tube for interrogation by single-scan ^{13}C NMR which revealed no hyperpolarised ^{13}C signals for any species. This is attributed to the long times (~90 s) required to perform these non-optimised hydrolysis and separation steps manually. This is compounded by the fact that the starting polarisation levels here are much lower than those achieved using techniques such as PHIP-SAH that use a similar procedure (~1% initial SABRE-Relay polarisation compared to 5% PHIP-SAH polarisation after hydrolysis and separation).[17] Additionally, the majority of ^{13}C polarisation in **2** resides on aliphatic ^{13}C sites that have T_1 values significantly shorter than the time required for the hydrolysis step. This suggests that, to produce SABRE-Relay hyperpolarised lactate *via* hydrolysis of **1-3** will firstly require further optimisation of their ^{13}C NMR signal gains for the longer-lived carbonyl site.

S11: References

(1) Savka, R.; Plenio, H. Facile Synthesis of [(NHC) MX (Cod)] and [(NHC) MCl (CO) 2](M= Rh, Ir; X= Cl, I) Complexes. *Dalton Trans.* **2015**, *44* (3), 891–893.

(2) Iali, W.; Rayner, P. J.; Alshehri, A.; Holmes, A. J.; Ruddlesden, A. J.; Duckett, S. B. Direct and Indirect Hyperpolarisation of Amines Using Para Hydrogen. *Chem. Sci.* **2018**, *9*, 3677–3684.

(3) Iali, W.; Rayner, P. J.; Duckett, S. B. Using Parahydrogen to Hyperpolarize Amines, Amides, Carboxylic Acids, Alcohols, Phosphates, and Carbonates. *Sci. Adv.* **2018**, *4* (1), eaao6250.

(4) Adamo, C.; Barone, V. Toward Reliable Density Functional Methods without Adjustable Parameters: The PBE0 Model. *J. Chem. Phys.* **1999**, *110* (13), 6158–6170.

(5) Grimme, S.; Antony, J.; Ehrlich, S.; Krieg, H. A Consistent and Accurate Ab Initio Parametrization of Density Functional Dispersion Correction (DFT-D) for the 94 Elements H-Pu. *J. Chem. Phys.* **2010**, *132* (15), 154104.

(6) Grimme, S.; Ehrlich, S.; Goerigk, L. Effect of the Damping Function in Dispersion Corrected Density Functional Theory. *J. Compu. Chem.* **2011**, *32* (7), 1456–1465.

(7) Weigend, F.; Furche, F.; Ahlrichs, R. Gaussian Basis Sets of Quadruple Zeta Valence Quality for Atoms H–Kr. *J. Chem. Phys.* **2003**, *119* (24), 12753–12762.

(8) TURBOMOLE V7.5 2020, a development of University of Karlsruhe and Forschungszentrum Karlsruhe GmbH, 1989-2007, TURBOMOLE GmbH, since 2007; available from https://www.turbomole.org.

(9) Dalton, a molecular electronic structure program, Release Dalton2019.alpha (2018), see http://daltonprogram.org.

(10) Aidas, K.; Angeli, C.; Bak, K. L.; Bakken, V.; Bast, R.; Boman, L.; Christiansen, O.; Cimiraglia, R.; Coriani, S.; Dahle, P. The Dalton Quantum Chemistry Program System. *Wiley Interdiscip. Rev. Comput. Mol. Sci.* **2014**, *4* (3), 269–284.

(11) Jensen, F. The Optimum Contraction of Basis Sets for Calculating Spin–Spin Coupling Constants. *Theoret. Chem. Acc.* **2010**, *126* (5), 371–382.

(12) Mathematica Version 12.3, Wolfram Research, Inc.: Champaign, Illinois 2021.

(13) Barskiy, D. A.; Knecht, S.; Yurkovskaya, A. v; Ivanov, K. L. SABRE: Chemical Kinetics and Spin Dynamics of the Formation of Hyperpolarization. *Prog. Nucl. Magn. Reson. Spec.* **2019**, *114–115*, 33–70.

(14) Ivanov, K. L.; Yurkovskaya, A. V.; Vieth, H.-M. Coherent Transfer of Hyperpolarization in Coupled Spin Systems at Variable Magnetic Field. *J. Chem. Phys.* **2008**, *128* (15), 154701.

(15) Reineri, F.; Boi, T.; Aime, S. Parahydrogen Induced Polarization of 13 C Carboxylate Resonance in Acetate and Pyruvate. *Nat. Commun.* **2015**, *6*, 5858.

(16) Cavallari, E.; Carrera, C.; Aime, S.; Reineri, F. 13C MR Hyperpolarization of Lactate by Using Parahydrogen and Metabolic Transformation in Vitro. *Chem. Eur. J.* **2017**, *23* (5), 1200–1204.

(17) Cavallari, E.; Carrera, C.; Aime, S.; Reineri, F. Studies to Enhance the Hyperpolarization Level in PHIP-SAH-Produced C13-Pyruvate. *J. Magn. Reson.* **2018**, *289*, 12–17.
