# Supplementary material for: Toward Optimizing and Understanding Reversible Hyperpolarization of Lactate Esters Relayed from para-Hydrogen
Source: J Phys Chem Lett. 2022 Jul 21;13(29):6859–66. doi: 10.1021/acs.jpclett.2c01442 (PMC9340809; doi:10.1021/acs.jpclett.2c01442)
Supplement: Supplementary file 2 — jz2c01442_si_002.pdf [file jz2c01442_si_002.pdf]

Spin polarization transfer and spectra of lactate esters

This program is part of the supplementary information for the article "Towards Optimising and Understanding Reversible Hyperpolarisation of Lactate Esters Relayed from Parahydrogen".

This notebook can be used to calculate spin magnetization as a function of time and ^{1}H-NMR-spectra for lactate esters, as described in the main article and associated supplementary information.

Has been run with Mathematica 12.3.

MIT License

Copyright © 2022 S. Karl-Mikael Svensson and Juha Vaara

Permission is hereby granted, free of charge, to any person obtaining a copy of this software and associated documentation files (the "Software"), to deal in the Software without restriction, including without limitation the rights to use, copy, modify, merge, publish, distribute, sublicense, and/or sell copies of the Software, and to permit persons to whom the Software is furnished to do so, subject to the following conditions:

The above copyright notice and this permission notice shall be included in all copies or substantial portions of the Software.

THE SOFTWARE IS PROVIDED "AS IS", WITHOUT WARRANTY OF ANY KIND, EXPRESS OR IMPLIED, INCLUDING BUT NOT LIMITED TO THE WARRANTIES OF MERCHANTABILITY, FITNESS FOR A PARTICULAR PURPOSE AND NONINFRINGEMENT. IN NO EVENT SHALL THE AUTHORS OR COPYRIGHT HOLDERS BE LIABLE FOR ANY CLAIM, DAMAGES OR OTHER LIABILITY, WHETHER IN AN ACTION OF CONTRACT, TORT OR OTHERWISE, ARISING FROM, OUT OF OR IN CONNECTION WITH THE SOFTWARE OR THE USE OR OTHER DEALINGS IN THE SOFTWARE.

Preliminary commands

```
In[ ]:= $HistoryLength = 0;
```

```
In[ ]:= Remove["Global`*"];
In[ ]:= $MachinePrecision
In[ ]:= MemoryAvailable []
```

Constants

```
In[ ]:= Element[hbar, Reals];
        Element[kB, Reals];
        Element[mu0, Reals];
        Element[muN, Reals];

In[ ]:= numConst = {
          (*CODATA 2018*)
          hbar → (6.62607015 * 10^(-34) / (2 * Pi))(*Js*),
          kB → 1.380649 * 10^(-23)(*JK^-1*),
          mu0 → 1.25663706212 * 10^(-6)(*NA^-2=Jm^-1s^2C^-2*),
          muN → 5.0507837461 * 10^(-27)(*JT^-1*)
        };
        numVal = numConst;
```

Colors for graphs taken from https://jfly.uni-koeln.de/color/ (accessed March 2022):

```
In[ ]:= black = RGBColor[0, 0, 0];
        orange = RGBColor[0.9, 0.6, 0];
        bluishGreen = RGBColor[0, 0.6, 0.5];
        blue = RGBColor[0, 0.45, 0.7];
        vermillion = RGBColor[0.8, 0.4, 0];
        reddishPurple = RGBColor[0.8, 0.6, 0.7];
        colors = {black, orange, bluishGreen, blue, vermillion, reddishPurple};
```

Cartesian basis for hydrogen-1 and carbon-13

```
In[ ]:= paulix = SparseArray[FullSimplify[{{0, 1}, {1, 0}}]];
        pauliy = SparseArray[FullSimplify[(1 / I) * {{0, 1}, {-1, 0}}]];
        pauliz = SparseArray[FullSimplify[{{1, 0}, {0, -1}}]];
        pauli1 = SparseArray[FullSimplify[{{1, 0}, {0, 1}}]];

In[ ]:= pauliBasis = {pauli1, paulix, pauliy, pauliz};
```

```
In[ ]:= kOH = 1;
        kHa1 = 2;
        kHa2 = 3;
        kHa3 = 4;
        kHb = 5;
        nspins = 5;
        kx = 2;
        ky = 3;
        kz = 4;
```

```
In[ ]:= hydrogens = {kOH, kHa1, kHa2, kHa3, kHb};
        carbons = {};
```

Basis set of operators

```
In[ ]:= basCidentity = IdentityMatrix[2^nspins, SparseArray];
```

```
In[ ]:= basC1spin[a_, b_] := basC1spin[a, b] =
          KroneckerProduct[KroneckerProduct[IdentityMatrix[2^(a - 1), SparseArray],
            pauliBasis[[b]]], IdentityMatrix[2^(nspins - a), SparseArray]];
```

```
In[ ]:= basC2spin[a_, b_, c_, d_] := basC2spin[a, b, c, d] = basC1spin[a, b].basC1spin[c, d];
```

Functions

Turn a matrix into a vector:

```
In[ ]:= vectorize[matrix_] := Flatten[Transpose[matrix]];
```

Turn a vector into a matrix:

```
In[ ]:= unvectorize[vector_] :=
          Transpose[ArrayReshape[vector, {Sqrt[Length[vector]], Sqrt[Length[vector]]}]];
```

Normalization factor for operator/matrix:

```
In[ ]:= norm[a_] := Sqrt[Tr[ConjugateTranspose[a].a]];
```

Combinations of functions:

```
In[ ]:= conjTransVecBasC1spin[a_, b_] := conjTransVecBasC1spin[a, b] =
          ConjugateTranspose[vectorize[ConjugateTranspose[basC1spin[a, b]]]];
```

```
In[ ]:= normBasC1spin[a_, b_] := normBasC1spin[a, b] = norm[basC1spin[a, b]];
```

Lorentzian function:

```
In[ ]:= lorentzian[scale_, width_, center_, x_] :=
          scale * (1 / Pi) * (width / 2) / ((x - center)^2 + (width / 2)^2);
```

```
In[ ]:= FullSimplify[lorentzian[a, b, c, x]]
```

Integration using the trapezoidal rule:

```
In[ ]:= integrateTrapezoidalRule [functionArray_ , dx_] := dx * (
    (1 / 2) * (functionArray [[1]] + functionArray [[-1]])
     + Sum[functionArray [[i]], {i , 2, Length[functionArray ] - 1}]
   )
```

The Hamiltonian and its parameters

Zeeman-coupling

```
In[ ]:= Element[b0, Reals];
Element[gammaC , Reals];
Element[gammaH , Reals];
```

```
In[ ]:= gamma = ConstantArray [0, {nspins , nspins}];
Do[gamma[[j , j]] = gammaH , {j , hydrogens }];
Do[gamma[[j , j]] = gammaC , {j , carbons }];
gamma = SparseArray [gamma];
(*MatrixForm [gamma]*)
```

```
In[ ]:= numGamma = {
    gammaH → 2.6752218744 * 10 ^ 8 (*Hz/T*)(*CODATA 2018*),
    gammaC → Replace [0.702412 * muN * 2 / hbar , numVal , Infinity]
    (*Hz/T*)(*CRC Handbook of Chemistry and Physics ,
  102nd Edition , 2021*)
   };
numVal = Join[numVal , numGamma ];
```

Chemical shift - isotropic

```
In[ ]:= deltaCS = Table[Subscript [dCS , i], {i , nspins}];
Do[Element [deltaCS [[i]], Reals], {i , nspins}];
```

```
In[ ]:= deltaCS = Replace [deltaCS , {
      deltaCS [[kHa2]] → deltaCS [[kHa1]],
      deltaCS [[kHa3]] → deltaCS [[kHa1]]
    }, Infinity];
```

The chemical shift reference is irrelevant for the homonuclear parts of the system, and for the heteronu - clear parts it is negligible.

```
In[ ]:= numDelta = {
    (*Methyl ester*)
    deltaCS⟦kOH⟧ → 3.42 * 10^(-6)(*ppm*10^6*),
    deltaCS⟦kHa1⟧ → 1.38 * 10^(-6)(*ppm*10^6*),
    deltaCS⟦kHb⟧ → 4.28 * 10^(-6)(*ppm*10^6*)(*,*)
    (*deltaCS⟦kCa⟧→20.13*10^(-6) (*ppm*10^6*)*)
    (*deltaCS⟦kCb⟧→66.79*10^(-6) (*ppm*10^6*)*)
    (*deltaCS⟦kCc⟧→176.00*10^(-6) (*ppm*10^6*)*)
    (*Ethyl ester*)
    (*deltaCS⟦kOH⟧→2.76*10^(-6)(*ppm*10^6*),
    deltaCS⟦kHa1⟧→1.41 *10^(-6)(*ppm*10^6*),
    deltaCS⟦kHb⟧→4.25 *10^(-6)(*ppm*10^6*)*)
    (*Butyl ester*)
    (*deltaCS⟦kOH⟧→3.15*10^(-6)(*ppm*10^6*),
    deltaCS⟦kHa1⟧→1.40 *10^(-6)(*ppm*10^6*),
    deltaCS⟦kHb⟧→4.26 *10^(-6)(*ppm*10^6*)*)
   };
  numVal = Join[numVal, numDelta];
```

```
In[ ]:= hZeeman = ConstantArray[0, {2^nspins, 2^nspins}];
  Do[hZeeman += FullSimplify[-gamma⟦j, j⟧ * (1 + deltaCS⟦j⟧) * b0 * (hbar / 2) * basC1spin[j, kz]],
    {j, 1, nspins}];
  hZeeman = FullSimplify[SparseArray[hZeeman]];
  (*MatrixForm[hZeeman]*)
```

Rotating frame

```
In[ ]:= AbsoluteTiming[hFrameH = ConstantArray[0, {2^nspins, 2^nspins}, SparseArray];
   Do[hFrameH += FullSimplify[-gammaH * b0 * (hbar / 2) * basC1spin[j, kz]], {j, 1, nspins}];
   hFrameH = FullSimplify[hFrameH];]
```

J-coupling

J-coupling is assumed to be isotropic and heteronuclear. The J-coupling between magnetically equivalent spins affect the thermal equilibrium, but it does not affect the dynamics.

```
In[ ]:= jCoupling = Table[Subscript[jC, i, j], {i, nspins}, {j, nspins}];
  Do[jCoupling⟦i, i⟧ = 0, {i, nspins}];
  Do[Do[Element[jCoupling⟦j, i⟧, Reals], {j, i - 1}], {i, nspins}];
  Do[Do[jCoupling⟦i, j⟧ = jCoupling⟦j, i⟧, {j, i - 1}], {i, nspins}];
  jCoupling = SparseArray[jCoupling];
```

```
In[ ]:= jCoupling = Normal[jCoupling];
Do[
  jCoupling = Replace[jCoupling, Subscript[jC, i, j] → Subscript[jC, kHa1, j], Infinity],
  {i, {kHa2, kHa3}}, {j, Join[carbons, hydrogens]}];
Do[jCoupling = Replace[jCoupling, Subscript[jC, j, i] → Subscript[jC, j, kHa1], Infinity],
  {i, {kHa2, kHa3}}, {j, Join[carbons, hydrogens]}];
jCouplingDynamics = Replace[jCoupling, Subscript[jC, kHa1, kHa1] → 0, Infinity];
jCoupling = SparseArray[jCoupling];
jCouplingDynamics = SparseArray[jCouplingDynamics];
(*MatrixForm[jCoupling]*)
(*MatrixForm[jCouplingDynamics]*)

In[ ]:= numJcoupling = {
    jCoupling[[kOH, kHa1]] → 1.0(*Hz*),
    jCoupling[[kOH, kHb]] → -0.5(*Hz*),
    jCoupling[[kHa1, kHb]] → 7.4(*Hz*),
    jCoupling[[kHa1, kHa2]] → -14.1(*Hz*)(*,*)
    (*jCoupling[[kOH,kCa]]→7.1 (*Hz*),
    jCoupling[[kHa1,kCa]]→126.3(*Hz*),
    jCoupling[[kHb,kCa]]→-4.4(*Hz*)*)
    (*jCoupling[[kOH,kCb]]→-2.7 (*Hz*),
    jCoupling[[kHa1,kCb]]→-4.8(*Hz*),
    jCoupling[[kHb,kCb]]→136.3 (*Hz*)*)
    (*jCoupling[[kOH,kCc]]→5.2 (*Hz*),
    jCoupling[[kHa1,kCc]]→5.3(*Hz*),
    jCoupling[[kHb,kCc]]→-4.2(*Hz*)*)
    (*Ethyl ester*)
    (*jCoupling[[kOH,kHa1]]→0.9(*Hz*),
    jCoupling[[kOH,kHb]]→-0.4(*Hz*),
    jCoupling[[kHa1,kHb]]→7.4(*Hz*),
    jCoupling[[kHa1,kHa2]]→-14.04(*Hz*)*)
    (*Butyl ester*)
    (*jCoupling[[kOH,kHa1]]→0.9(*Hz*),
    jCoupling[[kOH,kHb]]→-0.4(*Hz*),
    jCoupling[[kHa1,kHb]]→7.4(*Hz*),
    jCoupling[[kHa1,kHa2]]→-14.03(*Hz*)*)
  };
numVal = Join[numVal, numJcoupling];
```

```
In[ ]:= AbsoluteTiming[hJcoupling = ConstantArray[0, {2^nspins, 2^nspins}];
  Do[Do[
    hJcoupling += FullSimplify[2 * Pi * jCoupling[[i, j]] * (hbar / 2^2) * basC2spin[i, k, j, k]],
    {j, i + 1, nspins}, {k, {kx, ky, kz}}], {i, 1, nspins - 1}];
  hJcoupling = FullSimplify[SparseArray[hJcoupling]];]
(*MatrixForm[hJcoupling]*)
```

```
In[ ]:= AbsoluteTiming[hJcouplingDynamics = ConstantArray[0, {2^nspins, 2^nspins}];
  Do[Do[hJcouplingDynamics +=
     FullSimplify[2 * Pi * jCouplingDynamics[[i, j]] * (hbar / 2^2) * basC2spin[i, k, j, k]],
    {j, i + 1, nspins}, {k, {kx, ky, kz}}], {i, 1, nspins - 1}];
  hJcouplingDynamics = FullSimplify[SparseArray[hJcouplingDynamics]];]
```

Hamiltonian

```
In[ ]:= AbsoluteTiming[
  hGeneral = SparseArray[FullSimplify[SymmetrizedArray[hZeeman + hJcoupling]]]]
```

```
In[ ]:= AbsoluteTiming[hGeneralDynamics =
   SparseArray[FullSimplify[SymmetrizedArray[hZeeman + hJcouplingDynamics]]]]
```

```
In[ ]:= MemoryInUse[]
```

```
In[ ]:= Share[]
```

Liouvillian

```
In[ ]:= AbsoluteTiming[
  liouvillianGeneral =
    SparseArray[FullSimplify[-(I / hbar) * SymmetrizedArray[FullSimplify[
          KroneckerProduct[IdentityMatrix[2^nspins, SparseArray], hGeneralDynamics]] -
         FullSimplify[KroneckerProduct[Transpose[hGeneralDynamics],
           IdentityMatrix[2^nspins, SparseArray]]]]]];
]
```

```
In[ ]:= AbsoluteTiming[
  liouvillianGeneral = FullSimplify[liouvillianGeneral];
]
```

```
In[ ]:= MemoryInUse[]
```

```
In[ ]:= Share[]
```

Polarization

Infinite temperature approximation.

```
Element[tempPol, Reals];
Element[bPolz, Reals];
```

```
numPol = {
    tempPol → 298 (*K*),
    bPolz → 6.5 * 10^(-3) (*T*)
  };
numVal = Join[numVal, numPol];
```

Initial density

Degree of initial polarization on OH:

```
poldeg = 1;
```

Thermal density:

```
(*hPol=
  SparseArray[Replace[Replace[Normal[hGeneral],b0→bPolz,Infinity],numVal,Infinity]];*)
```

```
(*AbsoluteTiming[boltzOpPol=MatrixExp[-hPol/Replace[kB*tempPol,numVal,Infinity]];]*)
```

```
(*zPol=Tr[boltzOpPol];*)
```

```
(*rhoInitPol=boltzOpPol/zPol;*)
```

Polarized OH and all other spins completely unpolarized. (Corresponds to infinite temperature.)

```
rhoInitPol = poldeg * basC1spin[kOH, kz] / norm[basC1spin[kOH, kz]]^2 +
    basCidentity / norm[basCidentity]^2;
```

```
Tr[rhoInitPol]
```

```
Tr[((hbar / 2) * basC1spin[kOH, kx]).rhoInitPol]
```

```
Tr[((hbar / 2) * basC1spin[kOH, ky]).rhoInitPol]
```

```
Tr[((hbar / 2) * basC1spin[kOH, kz]).rhoInitPol]
```

```
liouvillianPol = SparseArray[
  Replace[Replace[Normal[liouvillianGeneral], b0 → bPolz, Infinity], numVal, Infinity]]
```

```
MemoryInUse[]
```

```
Share[]
```

Time steps

```
timePolTotal = 10(*s*);
```

```
In[ ]:= numDataPointsPol = 2^10

In[ ]:= dtimeDataPol = timePolTotal / (numDataPointsPol - 1.)(*s*)

In[ ]:= timePointsPol = Range[0, (numDataPointsPol - 1) * dtimeDataPol , dtimeDataPol ];
```

Time propagation and calculation of relevant quantities

Create short-time-propagator

```
In[ ]:= AbsoluteTiming [propagator = SparseArray [MatrixExp [dtimeDataPol * liouvillianPol ]];]
```

Spin transfer during polarization

```
In[ ]:= spinTransferPolx = ConstantArray [0, {nspins , numDataPointsPol }];
spinTransferPoly = ConstantArray [0, {nspins , numDataPointsPol }];
spinTransferPolz = ConstantArray [0, {nspins , numDataPointsPol }];

In[ ]:= rhoPoltVec = vectorize [rhoInitPol ];

In[ ]:= AbsoluteTiming [
  Do[
   spinTransferPolx [[s]][[1]] = 0.5 * conjTransVecBasC1spin [s, kx].rhoPoltVec , {s, nspins}];
  Do[spinTransferPoly [[s]][[1]] = 0.5 * conjTransVecBasC1spin [s, ky].rhoPoltVec ,
   {s, nspins}];
  Do[spinTransferPolz [[s]][[1]] = 0.5 * conjTransVecBasC1spin [s, kz].rhoPoltVec , {s, nspins}];
 ]

In[ ]:= AbsoluteTiming [Do[
    rhoPoltVec = propagator .rhoPoltVec ;
    Do[spinTransferPolx [[s]][[i]] =
      0.5 * conjTransVecBasC1spin [s, kx].rhoPoltVec , {s, nspins}];
    Do[spinTransferPoly [[s]][[i]] = 0.5 * conjTransVecBasC1spin [s, ky].rhoPoltVec ,
     {s, nspins}];
    Do[spinTransferPolz [[s]][[i]] = 0.5 * conjTransVecBasC1spin [s, kz].rhoPoltVec ,
     {s, nspins}];
    , {i, 2, numDataPointsPol }];]

In[ ]:= rhoPolt = unvectorize [rhoPoltVec ];

In[ ]:= Remove[propagator , liouvillianPol , rhoPoltVec ];

In[ ]:= MemoryInUse []

In[ ]:= Share[]
```

Spin polarization graphs

x-direction

```
In[ ]:= ListLinePlot[{Transpose[{timePointsPol, Re[Total[spinTransferPolx, {1}]]}],
    Transpose[{timePointsPol, Re[spinTransferPolx[[kOH]]]}], Transpose[{timePointsPol,
      Re[spinTransferPolx[[kHa1]] + spinTransferPolx[[kHa2]] + spinTransferPolx[[kHa3]]]}],
    Transpose[{timePointsPol, Re[spinTransferPolx[[kHb]]]}]},
  PlotRange → {{0, 10}, Full}, PlotStyle → colors,
  PlotLabels → Placed[{Style["Total", colors[[1]]], Style["OH", colors[[2]]],
      Style["Ha", colors[[3]]], Style["Hb", colors[[4]]]}, {{Scaled[1], Before}}],
  AxesLabel → {"t/s", "spin polarization/ℏ"}]
```

y-direction

```
In[ ]:= ListLinePlot[{Transpose[{timePointsPol, Re[Total[spinTransferPoly, {1}]]}],
    Transpose[{timePointsPol, Re[spinTransferPoly[[kOH]]]}], Transpose[{timePointsPol,
      Re[spinTransferPoly[[kHa1]] + spinTransferPoly[[kHa2]] + spinTransferPoly[[kHa3]]]}],
    Transpose[{timePointsPol, Re[spinTransferPoly[[kHb]]]}]},
  PlotRange → {{0, 10}, Full}, PlotStyle → colors,
  PlotLabels → Placed[{Style["Total", colors[[1]]], Style["OH", colors[[2]]],
      Style["Ha", colors[[3]]], Style["Hb", colors[[4]]]}, {{Scaled[1], Before}}],
  AxesLabel → {"t/s", "spin polarization/ℏ"}]
```

z-direction

```
In[ ]:= ListLinePlot[{Transpose[{timePointsPol, Re[Total[spinTransferPolz, {1}]]}],
    Transpose[{timePointsPol, Re[spinTransferPolz[[kOH]]]}], Transpose[{timePointsPol,
      Re[spinTransferPolz[[kHa1]] + spinTransferPolz[[kHa2]] + spinTransferPolz[[kHa3]]]}],
    Transpose[{timePointsPol, Re[spinTransferPolz[[kHb]]]}]},
  PlotRange → {{0, 10}, Full}, PlotStyle → colors,
  PlotLabels → Placed[{Style["Total", colors[[1]]], Style["OH", colors[[2]]],
      Style["Ha", colors[[3]]], Style["Hb", colors[[4]]]}, {{Scaled[1], Before}}],
  AxesLabel → {"t/s", "spin polarization/ℏ"}]
```

Exporting results to file

In[ ]:=
```
(*outData=ConstantArray[0,{numDataPointsPol+1,7}];
outData⟦1⟧={"#time","Rex","Imx","Rey","Imy","Rez","Imz"};
Do[
 outData⟦i+1,1⟧=timePointsPol⟦i⟧;
 outData⟦i+1,2⟧=Re[spinTransferPolx⟦kOH,i⟧];
 outData⟦i+1,3⟧=Im[spinTransferPolx⟦kOH,i⟧];
 outData⟦i+1,4⟧=Re[spinTransferPoly⟦kOH,i⟧];
 outData⟦i+1,5⟧=Im[spinTransferPoly⟦kOH,i⟧];
 outData⟦i+1,6⟧=Re[spinTransferPolz⟦kOH,i⟧];
 outData⟦i+1,7⟧=Im[spinTransferPolz⟦kOH,i⟧];
 ,{i,1,numDataPointsPol}];
Export["spinTransferPolOH_no_C.tsv",outData,"TSV",OverwriteTarget→"KeepBoth"]*)
```

In[ ]:=
```
(*outData=ConstantArray[0,{numDataPointsPol+1,7}];
outData⟦1⟧={"#time","Rex","Imx","Rey","Imy","Rez","Imz"};
Do[
 outData⟦i+1,1⟧=timePointsPol⟦i⟧;
 outData⟦i+1,2⟧=
  Re[spinTransferPolx⟦kHa1,i⟧+spinTransferPolx⟦kHa2,i⟧+spinTransferPolx⟦kHa3,i⟧];
 outData⟦i+1,3⟧=Im[spinTransferPolx⟦kHa1,i⟧+
    spinTransferPolx⟦kHa2,i⟧+spinTransferPolx⟦kHa3,i⟧];
 outData⟦i+1,4⟧=Re[spinTransferPoly⟦kHa1,i⟧+spinTransferPoly⟦kHa2,i⟧+
    spinTransferPoly⟦kHa3,i⟧];
 outData⟦i+1,5⟧=Im[spinTransferPoly⟦kHa1,i⟧+spinTransferPoly⟦kHa2,i⟧+
    spinTransferPoly⟦kHa3,i⟧];
 outData⟦i+1,6⟧=Re[spinTransferPolz⟦kHa1,i⟧+spinTransferPolz⟦kHa2,i⟧+
    spinTransferPolz⟦kHa3,i⟧];
 outData⟦i+1,7⟧=Im[spinTransferPolz⟦kHa1,i⟧+spinTransferPolz⟦kHa2,i⟧+
    spinTransferPolz⟦kHa3,i⟧];
 ,{i,1,numDataPointsPol}];
Export["spinTransferPolHa_no_C.tsv",outData,"TSV",OverwriteTarget→"KeepBoth"]*)
```

```
In[ ]:= (*outData=ConstantArray[0,{numDataPointsPol+1,7}];
outData⟦1⟧={"#time","Rex","Imx","Rey","Imy","Rez","Imz"};
Do[
 outData⟦i+1,1⟧=timePointsPol⟦i⟧;
 outData⟦i+1,2⟧=Re[spinTransferPolx⟦kHb,i⟧];
 outData⟦i+1,3⟧=Im[spinTransferPolx⟦kHb,i⟧];
 outData⟦i+1,4⟧=Re[spinTransferPoly⟦kHb,i⟧];
 outData⟦i+1,5⟧=Im[spinTransferPoly⟦kHb,i⟧];
 outData⟦i+1,6⟧=Re[spinTransferPolz⟦kHb,i⟧];
 outData⟦i+1,7⟧=Im[spinTransferPolz⟦kHb,i⟧];
 ,{i,1,numDataPointsPol}];
Export["spinTransferPolHb_no_C.tsv",outData,"TSV",OverwriteTarget→"KeepBoth"]*)
```

```
In[ ]:= (*Export["spinTransferPolz_no_C.eps",
 ListLinePlot[{Transpose[{timePointsPol,Re[Total[spinTransferPolz,{1}]]}],
   Transpose[{timePointsPol,Re[spinTransferPolz⟦kOH⟧]}],Transpose[{timePointsPol,
     Re[spinTransferPolz⟦kHa1⟧+spinTransferPolz⟦kHa2⟧+spinTransferPolz⟦kHa3⟧]}],
   Transpose[{timePointsPol,Re[spinTransferPolz⟦kHb⟧]}]},PlotRange→{{0,10},Full},
  PlotStyle→colors,PlotLabels→Placed[{"Total","OH","Ha","Hb"},{{Scaled[1],Before}}],
  AxesLabel→{"t/s","spin polarization/ħ"}]
 ,"EPS",OverwriteTarget→"KeepBoth"]*)
```

```
In[ ]:= (*Export["spinTransferPolz_no_C.png",
 ListLinePlot[{Transpose[{timePointsPol,Re[Total[spinTransferPolz,{1}]]}],
   Transpose[{timePointsPol,Re[spinTransferPolz⟦kOH⟧]}],Transpose[{timePointsPol,
     Re[spinTransferPolz⟦kHa1⟧+spinTransferPolz⟦kHa2⟧+spinTransferPolz⟦kHa3⟧]}],
   Transpose[{timePointsPol,Re[spinTransferPolz⟦kHb⟧]}]},PlotRange→{{0,10},Full},
  PlotStyle→colors,PlotLabels→Placed[{"Total","OH","Ha","Hb"},{{Scaled[1],Before}}],
  AxesLabel→{"t/s","spin polarization/ħ"}]
 ,"PNG",OverwriteTarget→"KeepBoth"]*)
```

```
In[ ]:= Share[]
```

Removing coherences to simulate chemical exchange

```
In[ ]:= hPol = SparseArray[Replace[Normal[hGeneral], b0 → bPolz, Infinity]];
```

```
In[ ]:= hPol = SparseArray[Replace[Normal[hPol], numVal, Infinity]]
```

```
In[ ]:= AbsoluteTiming[{eigVal, eigVec} = Eigensystem[SymmetrizedArray[hPol]];]
```

```
In[ ]:= eigVec = SparseArray[eigVec];
```

Since the populations in the eigenbasis are constant during propagation the initial density can be used.

```
In[ ]:= rhoPoltEigenbasis = eigVec.rhoInitPol.ConjugateTranspose[eigVec];
```

```
In[ ]:= rhoPoltEigenbasisReduced = DiagonalMatrix[Diagonal[rhoPoltEigenbasis]];

In[ ]:= rhoPoltReduced = ConjugateTranspose[eigVec].rhoPoltEigenbasisReduced.eigVec;

In[ ]:= rhoPolt = rhoPoltReduced;

In[ ]:= Remove[hPol, eigVal, eigVec, rhoPoltEigenbasis,
          rhoPoltEigenbasisReduced, rhoPoltReduced];

In[ ]:= MemoryInUse[]

In[ ]:= Share[]
```

Pulse: 90 degrees around x for H

```
In[ ]:= AbsoluteTiming[
          rotations = (1/2) * Sum[basC1spin[i, kx], {i, hydrogens}];
          rotSupOp = FullSimplify[
            -I * (Pi/2) * (FullSimplify[KroneckerProduct[IdentityMatrix[2^nspins, SparseArray],
                 rotations]] - FullSimplify[KroneckerProduct[
                 Transpose[rotations], IdentityMatrix[2^nspins, SparseArray]]])];
        ]

In[ ]:= AbsoluteTiming[rhoAP = unvectorize[MatrixExp[rotSupOp, vectorize[rhoPolt]]];]

In[ ]:= Remove[rhoPolt, rotations, rotSupOp];

In[ ]:= MemoryInUse[]

In[ ]:= Share[]
```

Detection

```
In[ ]:= Element[tempDet, Reals];
        Element[bDetz, Reals];

In[ ]:= numDet = {
            tempDet → 298 (*K*),
            bDetz → 9.4 (*T*)
          };
        numVal = Join[numVal, numDet];

In[ ]:= hDetH = FullSimplify[
          SparseArray[Replace[Normal[hGeneralDynamics - hFrameH], b0 → bDetz, Infinity]]]

In[ ]:= MemoryInUse[]

In[ ]:= Share[]
```

Liouvillian

```
In[ ]:= liouvillianDet = SparseArray[
          Replace[Replace[Normal[liouvillianGeneral], b0 → bDetz, Infinity], numVal, Infinity]]

In[ ]:= AbsoluteTiming[
         liouvillianDetH = SparseArray[
            FullSimplify[-(I / hbar) * (FullSimplify[SymmetrizedArray[KroneckerProduct[
                   IdentityMatrix[2 ^ nspins, SparseArray], hDetH]]] -
                FullSimplify[SymmetrizedArray[KroneckerProduct[Transpose[hDetH],
                   IdentityMatrix[2 ^ nspins, SparseArray]]]])]];
        ]

In[ ]:= AbsoluteTiming[
         liouvillianDetH = FullSimplify[liouvillianDetH];
        ]

In[ ]:= liouvillianDetH = SparseArray[Replace[Normal[liouvillianDetH], numVal, Infinity]]

In[ ]:= Remove[hDetH];

In[ ]:= MemoryInUse[]

In[ ]:= Share[]
```

Time steps

```
In[ ]:= timeDetTotal = 30; (*s*)
```

For spin polarization calculations

```
In[ ]:= numDataPointsDet = 2 ^ 10

In[ ]:= dtimeDataDet = timeDetTotal / (numDataPointsDet - 1.)(*s*)

In[ ]:= timePointsDet = Range[0, (numDataPointsDet - 1) * dtimeDataDet, dtimeDataDet];
```

For ^{1}H-spectrum calculations

Intended Nyquist - frequency in the H-frame:

```
In[ ]:= iNF = 2 * Replace[gammaH * bDetz / (2 * Pi), numVal, Infinity] *
           Max[Abs[Replace[deltaCS, numVal, Infinity]]](*Hz*);
        dtTemp = 1 / (2 * iNF) (*s*);

In[ ]:= numDataPointsDetH = 2 ^ Ceiling[Log2[timeDetTotal / dtTemp + 1]]

In[ ]:= dtimeDataDetH = timeDetTotal / (numDataPointsDetH - 1.)(*s*)

In[ ]:= timePointsDetH = Range[0, (numDataPointsDetH - 1) * dtimeDataDetH, dtimeDataDetH];
```

```
In[ ]:= MemoryInUse[]

In[ ]:= Share[]
```

Time propagation and calculation of spin polarization

Create short-time-propagator

```
In[ ]:= AbsoluteTiming[propagator = SparseArray[MatrixExp[dtimeDataDet * liouvillianDet]];]
```

Spin transfer during detection

```
In[ ]:= spinTransferDetx = ConstantArray[0, {nspins, numDataPointsDet}];
spinTransferDety = ConstantArray[0, {nspins, numDataPointsDet}];
spinTransferDetz = ConstantArray[0, {nspins, numDataPointsDet}];

In[ ]:= rhoDettVec = vectorize[rhoAP];

In[ ]:= AbsoluteTiming[
  Do[
   spinTransferDetx[[s]][[1]] = 0.5 * conjTransVecBasC1spin[s, kx].rhoDettVec, {s, nspins}];
  Do[spinTransferDety[[s]][[1]] = 0.5 * conjTransVecBasC1spin[s, ky].rhoDettVec,
   {s, nspins}];
  Do[spinTransferDetz[[s]][[1]] = 0.5 * conjTransVecBasC1spin[s, kz].rhoDettVec, {s, nspins}];
 ]

In[ ]:= AbsoluteTiming[Do[
    rhoDettVec = propagator.rhoDettVec;
    Do[spinTransferDetx[[s]][[i]] =
      0.5 * conjTransVecBasC1spin[s, kx].rhoDettVec, {s, nspins}];
    Do[spinTransferDety[[s]][[i]] = 0.5 * conjTransVecBasC1spin[s, ky].rhoDettVec,
     {s, nspins}];
    Do[spinTransferDetz[[s]][[i]] = 0.5 * conjTransVecBasC1spin[s, kz].rhoDettVec,
     {s, nspins}];
    , {i, 2, numDataPointsDet}];]

In[ ]:= Remove[propagator, rhoDettVec];

In[ ]:= MemoryInUse[]

In[ ]:= Share[]
```

Spin polarization graphs

```
In[ ]:= ListLinePlot[{Transpose[{timePointsDet, Re[Total[spinTransferDetx, {1}]]}],
      Transpose[{timePointsDet, Re[spinTransferDetx⟦kOH⟧]}], Transpose[{timePointsDet,
        Re[spinTransferDetx⟦kHa1⟧ + spinTransferDetx⟦kHa2⟧ + spinTransferDetx⟦kHa3⟧]}],
      Transpose[{timePointsDet, Re[spinTransferDetx⟦kHb⟧]}]},
     PlotRange → {Full, Full}, PlotStyle → colors,
     PlotLabels → Placed[{Style["Total", colors⟦1⟧], Style["OH", colors⟦2⟧],
        Style["Ha", colors⟦3⟧], Style["Hb", colors⟦4⟧]}, {{Scaled[1], Before}}],
     AxesLabel → {"t/s", "spin polarization/ℏ"}]
```

```
In[ ]:= ListLinePlot[{Transpose[{timePointsDet, Re[Total[spinTransferDety, {1}]]}],
      Transpose[{timePointsDet, Re[spinTransferDety⟦kOH⟧]}], Transpose[{timePointsDet,
        Re[spinTransferDety⟦kHa1⟧ + spinTransferDety⟦kHa2⟧ + spinTransferDety⟦kHa3⟧]}],
      Transpose[{timePointsDet, Re[spinTransferDety⟦kHb⟧]}]},
     PlotRange → {Full, Full}, PlotStyle → colors,
     PlotLabels → Placed[{Style["Total", colors⟦1⟧], Style["OH", colors⟦2⟧],
        Style["Ha", colors⟦3⟧], Style["Hb", colors⟦4⟧]}, {{Scaled[1], Before}}],
     AxesLabel → {"t/s", "spin polarization/ℏ"}]
```

```
In[ ]:= ListLinePlot[{Transpose[{timePointsDet, Re[Total[spinTransferDetz, {1}]]}],
      Transpose[{timePointsDet, Re[spinTransferDetz⟦kOH⟧]}], Transpose[{timePointsDet,
        Re[spinTransferDetz⟦kHa1⟧ + spinTransferDetz⟦kHa2⟧ + spinTransferDetz⟦kHa3⟧]}],
      Transpose[{timePointsDet, Re[spinTransferDetz⟦kHb⟧]}]},
     PlotRange → {Full, Full}, PlotStyle → colors,
     PlotLabels → Placed[{Style["Total", colors⟦1⟧], Style["OH", colors⟦2⟧],
        Style["Ha", colors⟦3⟧], Style["Hb", colors⟦4⟧]}, {{Scaled[1], Before}}],
     AxesLabel → {"t/s", "spin polarization/ℏ"}]
```

Time propagation and calculation of spectrum

Create short-time-propagator for H-frame

```
In[ ]:= AbsoluteTiming[propagator = SparseArray[MatrixExp[dtimeDataDetH * liouvillianDetH]];]
```

Relaxation

```
In[ ]:= t2 = Table[Subscript[t2e, i], {i, nspins}];
     Do[Element[t2⟦i⟧, Reals], {i, nspins}];
```

```
In[ ]:= t2 = Replace[t2, {
        t2⟦kHa2⟧ → t2⟦kHa1⟧,
        t2⟦kHa3⟧ → t2⟦kHa1⟧
       }, Infinity];
```

Setting the T_2 such that the peak widths will be ~0.1 Hz.

```
In[ ]:= numT2 = {
    t2⟦kOH⟧ → 10 / Pi(*s*),
    t2⟦kHa1⟧ → 10 / Pi(*s*),
    t2⟦kHb⟧ → 10 / Pi(*s*)
   };
  numVal = Join[numVal , numT2];
```

Discernable differences in frequency /Hz

```
In[ ]:= Replace[1 / (Pi * t2), numVal , Infinity]

In[ ]:= t2Num = Replace[t2, numVal , Infinity];
```

Signal from H during detection

```
In[ ]:= numbDens = 25 * 10 ^ (- 3)(*M*) * 6.02214076 * 10 ^ 23(*mol-1(2018 CODATA)*) * 10 ^ 6
   (*l/m^3*) (*Total unit: m^-3 (25 mM)*);

In[ ]:= signalHx = ConstantArray [0, numDataPointsDetH ];
  signalHy = ConstantArray [0, numDataPointsDetH ];
  signalH = ConstantArray [0, numDataPointsDetH ];

In[ ]:= rhoDettVec = vectorize [rhoAP];

In[ ]:= signalTemp = ConstantArray [0, Length[hydrogens ]];

In[ ]:= AbsoluteTiming [
   Do[signalTemp ⟦i⟧ = conjTransVecBasC1spin [hydrogens ⟦i⟧, kx].liouvillianDet .rhoDettVec /
      normBasC1spin [hydrogens ⟦i⟧, kx], {i, 1, Length[hydrogens ]}];
   signalHx ⟦1⟧ = Total[signalTemp ];
  ]

In[ ]:= AbsoluteTiming [
   Do[signalTemp ⟦i⟧ = conjTransVecBasC1spin [hydrogens ⟦i⟧, ky].liouvillianDet .rhoDettVec /
      normBasC1spin [hydrogens ⟦i⟧, ky], {i, 1, Length[hydrogens ]}];
   signalHy ⟦1⟧ = Total[signalTemp ];
  ]
```

```
In[ ]:= AbsoluteTiming[Do[
    rhoDettVec = propagator.rhoDettVec;
    tempmat = liouvillianDet.rhoDettVec;
    Do[signalTemp[[j]] =
      (conjTransVecBasC1spin[hydrogens[[j]], kx].tempmat / normBasC1spin[hydrogens[[j]], kx]) *
       Exp[-(i - 1) * dtimeDataDetH / t2Num[[j]]], {j, 1, Length[hydrogens]}];
    signalHx[[i]] = Total[signalTemp];
    Do[signalTemp[[j]] =
      (conjTransVecBasC1spin[hydrogens[[j]], ky].tempmat / normBasC1spin[hydrogens[[j]], ky]) *
       Exp[-(i - 1) * dtimeDataDetH / t2Num[[j]]], {j, 1, Length[hydrogens]}];
    signalHy[[i]] = Total[signalTemp];
    , {i, 2, numDataPointsDetH}];
   signalHx = -Replace[numbDens * gammaH * hbar, numVal, Infinity] * signalHx;
   signalHy = -Replace[numbDens * gammaH * hbar, numVal, Infinity] * signalHy;]

In[ ]:= Remove[propagator, liouvillianDet, liouvillianDetH, rhoDettVec];

In[ ]:= AbsoluteTiming[signalH = signalHx + I * signalHy;]

In[ ]:= Remove[signalHx, signalHy]

In[ ]:= ListLinePlot[Transpose[{timePointsDetH, Re[signalH]}],
   PlotRange → {Full, Full}, PlotStyle → colors[[4]],
   PlotLabels → Placed["FID", {{Scaled[1], Before}}], AxesLabel → {"t/s", "H-signal/arb."}]

In[ ]:= MemoryInUse[]

In[ ]:= Share[]
```

Spectrum

```
In[ ]:= nyquistFH = 1 / (2 * dtimeDataDetH) (*Hz*)

In[ ]:= dFH = 2 * nyquistFH / numDataPointsDetH (*Hz*)

In[ ]:= freqPointsH = Range[-(numDataPointsDetH / 2) * dFH, (numDataPointsDetH / 2) * dFH, dFH];

In[ ]:= freqPointsH = (freqPointsH / Replace[-gammaH * bDetz / (2 * Pi), numVal, Infinity]) * 10 ^ 6
    (*ppm*);

In[ ]:= dFH = (dFH / Replace[gammaH * bDetz / (2 * Pi), numVal, Infinity]) * 10 ^ 6(*ppm*);

In[ ]:= ftSignalH = dtimeDataDetH * Fourier[signalH, FourierParameters → {1, 1}];

In[ ]:= ftSignalH = Join[ftSignalH[[(numDataPointsDetH / 2 + 1) ;; numDataPointsDetH]],
     ftSignalH[[1 ;; (numDataPointsDetH / 2)]], {ftSignalH[[numDataPointsDetH / 2 + 1]]}];

In[ ]:= cutPosition = Position[Abs[freqPointsH - 5], Min[Abs[freqPointsH - 5]]][[1, 1]];
  spectrum = ftSignalH[[cutPosition ;; (Length[ftSignalH] - 1) / 2 + 1]];
  freqPointsSpectrum = freqPointsH[[cutPosition ;; (Length[freqPointsH] - 1) / 2 + 1]];
```

```
In[ ]:= MemoryInUse[]

In[ ]:= Share[]
```

The whole ^{1}H-spectrum

```
In[ ]:= ListLinePlot[Transpose[{freqPointsSpectrum , Re[spectrum]}], PlotRange → {Full, Full},
         PlotStyle → colors⟦4⟧, Axes → False, Frame → {True, False, False, False},
         FrameLabel → {"Chemical shift / ppm"}, ScalingFunctions → {"Reverse", Automatic}]

In[ ]:= integrateTrapezoidalRule[Re[spectrum], dFH]
```

H_b peaks

```
In[ ]:= ListLinePlot[Transpose[{freqPointsSpectrum , Re[spectrum]}],
         PlotRange → {{4.225, 4.325}, {0, 2.5 * 10 ^ 10}}, PlotStyle → colors⟦4⟧,
         AxesLabel → {"Chemical shift / ppm", "arb."},
         ScalingFunctions → {"Reverse", Automatic}]

In[ ]:= cutPositionUpper =
          Position[Abs[freqPointsSpectrum - 4.32], Min[Abs[freqPointsSpectrum - 4.32]]]⟦1, 1⟧;
        cutPositionLower = Position[Abs[freqPointsSpectrum - 4.24],
            Min[Abs[freqPointsSpectrum - 4.24]]]⟦1, 1⟧;

In[ ]:= peaks =
          FindPeaks[Abs[Re[spectrum⟦cutPositionUpper ;; cutPositionLower⟧]], 0, 0, -Infinity];

In[ ]:= Do[peaks⟦i, 2⟧ *= Sign[Re[spectrum⟦cutPositionUpper ;; cutPositionLower⟧]]⟦peaks⟦i, 1⟧⟧,
          {i, 1, Length[peaks⟦ ;; , 1⟧]}];
        peaks

In[ ]:= peakWidth =
          ((1 / (Pi * t2Num⟦kHb⟧)) / Replace[gammaH * bDetz / (2 * Pi), numVal, Infinity]) * 10 ^ 6;
        cutPositionUpper2 = Position[Abs[freqPointsSpectrum -
              freqPointsSpectrum⟦cutPositionUpper ;; cutPositionLower⟧⟦peaks⟦1, 1⟧⟧ -
              40 * peakWidth], Min[Abs[freqPointsSpectrum - freqPointsSpectrum⟦
                 cutPositionUpper ;; cutPositionLower⟧⟦peaks⟦1, 1⟧⟧ - 40 * peakWidth]]]⟦1, 1⟧;
        cutPositionLower2 = Position[Abs[freqPointsSpectrum - freqPointsSpectrum⟦
                cutPositionUpper ;; cutPositionLower⟧⟦peaks⟦-1, 1⟧⟧ + 40 * peakWidth],
            Min[Abs[freqPointsSpectrum - freqPointsSpectrum⟦cutPositionUpper ;;
                  cutPositionLower⟧⟦peaks⟦-1, 1⟧⟧ + 40 * peakWidth]]]⟦1, 1⟧;
```

```
In[ ]:= fittingFunction = 0;
Do[
  fittingFunction +=
   lorentzian[Subscript[a, i], Subscript[b, i], Subscript[c, i], chemShift];
  , {i, 1, Length[peaks]}];
(*fittingFunction *)
```

```
In[ ]:= fittingParameters = {};
Do[fittingParameters =
  Join[fittingParameters , {{Subscript[a, i], Pi * peaks⟦i, 2⟧ * peakWidth / 2}}];
 fittingParameters = Join[fittingParameters , {{Subscript[b, i], peakWidth}}];
 fittingParameters = Join[fittingParameters , {{Subscript[c, i],
     freqPointsSpectrum ⟦cutPositionUpper ;; cutPositionLower ⟧⟦peaks⟦i, 1⟧⟧}}];
 , {i, 1, Length[peaks]}]
(*fittingParameters *)
```

```
In[ ]:= fittedFunction = Normal[NonlinearModelFit [
    Transpose [{freqPointsSpectrum ⟦cutPositionUpper2 ;; cutPositionLower2 ⟧,
      Re[spectrum⟦cutPositionUpper2 ;; cutPositionLower2 ⟧]}],
    fittingFunction , fittingParameters , chemShift]]
```

```
In[ ]:= Show[ListPlot [Transpose [{freqPointsSpectrum ⟦cutPositionUpper2 ;; cutPositionLower2 ⟧,
      Re[spectrum⟦cutPositionUpper2 ;; cutPositionLower2 ⟧]}], PlotRange →
    {{freqPointsSpectrum ⟦cutPositionLower2 ⟧, freqPointsSpectrum ⟦cutPositionUpper2 ⟧},
      Full}, AxesLabel → {"Chemical shift / ppm", "arb."},
   ScalingFunctions → {"Reverse", Automatic},
   PlotStyle → Directive[colors⟦4⟧, PointSize[Medium]]],
  Plot[fittedFunction , {chemShift , freqPointsSpectrum ⟦cutPositionLower2 ⟧,
    freqPointsSpectrum ⟦cutPositionUpper2 ⟧}, PlotRange →
    {{freqPointsSpectrum ⟦cutPositionLower2 ⟧, freqPointsSpectrum ⟦cutPositionUpper2 ⟧},
      Full}, AxesLabel → {"Chemical shift / ppm", "arb."},
   ScalingFunctions → {"Reverse", Automatic}, PlotStyle → colors⟦2⟧]]
```

```
In[ ]:= Integrate [fittedFunction , {chemShift , -Infinity , Infinity }]
```

```
In[ ]:= integrateTrapezoidalRule [Re[spectrum]⟦cutPositionUpper2 ;; cutPositionLower2 ⟧, dFH]
```

OH peaks

```
In[ ]:= ListLinePlot [Transpose [{freqPointsSpectrum , Re[spectrum]}],
  PlotRange → {{3.41, 3.43}, Full}, PlotStyle → colors⟦4⟧,
  AxesLabel → {"Chemical shift / ppm", "arb."},
  ScalingFunctions → {"Reverse", Automatic}]
```

```
In[ ]:= cutPositionUpper =
          Position[Abs[freqPointsSpectrum - 3.43], Min[Abs[freqPointsSpectrum - 3.43]]]⟦1, 1⟧;
        cutPositionLower = Position[Abs[freqPointsSpectrum - 3.41],
            Min[Abs[freqPointsSpectrum - 3.41]]]⟦1, 1⟧;

In[ ]:= peaks = FindPeaks[Abs[Re[spectrum⟦cutPositionUpper ;; cutPositionLower⟧]]];

In[ ]:= Do[peaks⟦i, 2⟧ *= Sign[Re[spectrum⟦cutPositionUpper ;; cutPositionLower⟧]]⟦peaks⟦i, 1⟧⟧,
          {i, 1, Length[peaks⟦ ;; , 1⟧]}];
        peaks

In[ ]:= peakWidth =
          ((1 / (Pi * t2Num⟦kHb⟧)) / Replace[gammaH * bDetz / (2 * Pi), numVal, Infinity]) * 10 ^ 6;
        cutPositionUpper2 = Position[Abs[freqPointsSpectrum -
              freqPointsSpectrum⟦cutPositionUpper ;; cutPositionLower⟧⟦peaks⟦1, 1⟧⟧ -
              40 * peakWidth], Min[Abs[freqPointsSpectrum - freqPointsSpectrum⟦
                 cutPositionUpper ;; cutPositionLower⟧⟦peaks⟦1, 1⟧⟧ - 40 * peakWidth]]]⟦1, 1⟧;
        cutPositionLower2 = Position[Abs[freqPointsSpectrum - freqPointsSpectrum⟦
                cutPositionUpper ;; cutPositionLower⟧⟦peaks⟦-1, 1⟧⟧ + 40 * peakWidth],
            Min[Abs[freqPointsSpectrum - freqPointsSpectrum⟦cutPositionUpper ;;
                  cutPositionLower⟧⟦peaks⟦-1, 1⟧⟧ + 40 * peakWidth]]]⟦1, 1⟧;

In[ ]:= fittingFunction = 0;
        Do[
          fittingFunction +=
            lorentzian[Subscript[a, i], Subscript[b, i], Subscript[c, i], chemShift];
          , {i, 1, Length[peaks]}];
        (*fittingFunction*)

In[ ]:= fittingParameters = {};
        Do[fittingParameters =
          Join[fittingParameters, {{Subscript[a, i], Pi * peaks⟦i, 2⟧ * peakWidth / 2}}];
          fittingParameters = Join[fittingParameters, {{Subscript[b, i], peakWidth}}];
          fittingParameters = Join[fittingParameters, {{Subscript[c, i],
               freqPointsSpectrum⟦cutPositionUpper ;; cutPositionLower⟧⟦peaks⟦i, 1⟧⟧}}];
          , {i, 1, Length[peaks]}]
        (*fittingParameters*)

In[ ]:= fittedFunction = Normal[NonlinearModelFit[
           Transpose[{freqPointsSpectrum⟦cutPositionUpper2 ;; cutPositionLower2⟧,
              Re[spectrum⟦cutPositionUpper2 ;; cutPositionLower2⟧]}],
           fittingFunction, fittingParameters, chemShift]]
```

```
In[ ]:= Show[ListPlot[Transpose[{freqPointsSpectrum[[cutPositionUpper2 ;; cutPositionLower2]],
        Re[spectrum[[cutPositionUpper2 ;; cutPositionLower2]]]}], PlotRange →
      {{freqPointsSpectrum[[cutPositionLower2]], freqPointsSpectrum[[cutPositionUpper2]]},
        Full}, AxesLabel → {"Chemical shift / ppm", "arb."},
     ScalingFunctions → {"Reverse", Automatic},
     PlotStyle → Directive[colors[[4]], PointSize[Medium]]],
   Plot[fittedFunction, {chemShift, freqPointsSpectrum[[cutPositionLower2]],
      freqPointsSpectrum[[cutPositionUpper2]]}, PlotRange →
      {{freqPointsSpectrum[[cutPositionLower2]], freqPointsSpectrum[[cutPositionUpper2]]},
        Full}, AxesLabel → {"Chemical shift / ppm", "arb."},
     ScalingFunctions → {"Reverse", Automatic}, PlotStyle → colors[[2]]]]

In[ ]:= Integrate[fittedFunction, {chemShift, -Infinity, Infinity}]

In[ ]:= integrateTrapezoidalRule[Re[spectrum][[cutPositionUpper2 ;; cutPositionLower2]], dFH]
```

H_a peaks

```
In[ ]:= ListLinePlot[Transpose[{freqPointsSpectrum, Re[spectrum]}],
   PlotRange → {{1.36, 1.4}, Full}, PlotStyle → colors[[4]],
   AxesLabel → {"Chemical shift / ppm", "arb."},
   ScalingFunctions → {"Reverse", Automatic}]

In[ ]:= cutPositionUpper =
     Position[Abs[freqPointsSpectrum - 1.40], Min[Abs[freqPointsSpectrum - 1.40]]][[1, 1]];
  cutPositionLower = Position[Abs[freqPointsSpectrum - 1.36],
       Min[Abs[freqPointsSpectrum - 1.36]]][[1, 1]];

In[ ]:= peaks = FindPeaks[Abs[Re[spectrum[[cutPositionUpper ;; cutPositionLower]]]]];

In[ ]:= Do[peaks[[i, 2]] *= Sign[Re[spectrum[[cutPositionUpper ;; cutPositionLower]]][[peaks[[i, 1]]]]],
     {i, 1, Length[peaks[[ ;; , 1]]]}];
  peaks

In[ ]:= peakWidth =
     ((1 / (Pi * t2Num[[kHb]])) / Replace[gammaH * bDetz / (2 * Pi), numVal, Infinity]) * 10^6;
  cutPositionUpper2 = Position[Abs[freqPointsSpectrum -
          freqPointsSpectrum[[cutPositionUpper ;; cutPositionLower]][[peaks[[1, 1]]]] -
          40 * peakWidth], Min[Abs[freqPointsSpectrum - freqPointsSpectrum[[
              cutPositionUpper ;; cutPositionLower]][[peaks[[1, 1]]]] - 40 * peakWidth]]][[1, 1]];
  cutPositionLower2 = Position[Abs[freqPointsSpectrum - freqPointsSpectrum[[
             cutPositionUpper ;; cutPositionLower]][[peaks[[-1, 1]]]] + 40 * peakWidth],
       Min[Abs[freqPointsSpectrum - freqPointsSpectrum[[cutPositionUpper ;;
              cutPositionLower]][[peaks[[-1, 1]]]] + 40 * peakWidth]]][[1, 1]];
```

```
In[ ]:= fittingFunction = 0;
Do[
  fittingFunction +=
   lorentzian[Subscript[a, i], Subscript[b, i], Subscript[c, i], chemShift];
  , {i, 1, Length[peaks]}];
(*fittingFunction *)
```

```
In[ ]:= fittingParameters = {};
Do[fittingParameters =
  Join[fittingParameters , {{Subscript[a, i], Pi * peaks⟦i, 2⟧ * peakWidth / 2}}];
 fittingParameters = Join[fittingParameters , {{Subscript[b, i], peakWidth}}];
 fittingParameters = Join[fittingParameters , {{Subscript[c, i],
     freqPointsSpectrum ⟦cutPositionUpper ;; cutPositionLower ⟧⟦peaks⟦i, 1⟧⟧}}];
 , {i, 1, Length[peaks]}]
(*fittingParameters *)
```

```
In[ ]:= fittedFunction = Normal[NonlinearModelFit [
    Transpose [{freqPointsSpectrum ⟦cutPositionUpper2 ;; cutPositionLower2 ⟧,
      Re[spectrum⟦cutPositionUpper2 ;; cutPositionLower2 ⟧]}],
    fittingFunction , fittingParameters , chemShift]]
```

```
In[ ]:= Show[ListPlot [Transpose [{freqPointsSpectrum ⟦cutPositionUpper2 ;; cutPositionLower2 ⟧,
     Re[spectrum⟦cutPositionUpper2 ;; cutPositionLower2 ⟧]}], PlotRange →
    {{freqPointsSpectrum ⟦cutPositionLower2 ⟧, freqPointsSpectrum ⟦cutPositionUpper2 ⟧},
     Full}, AxesLabel → {"Chemical shift / ppm", "arb."},
   ScalingFunctions → {"Reverse", Automatic},
   PlotStyle → Directive [colors⟦4⟧, PointSize [Medium]]],
  Plot[fittedFunction , {chemShift , freqPointsSpectrum ⟦cutPositionLower2 ⟧,
    freqPointsSpectrum ⟦cutPositionUpper2 ⟧}, PlotRange →
    {{freqPointsSpectrum ⟦cutPositionLower2 ⟧, freqPointsSpectrum ⟦cutPositionUpper2 ⟧},
     Full}, AxesLabel → {"Chemical shift / ppm", "arb."},
   ScalingFunctions → {"Reverse", Automatic}, PlotStyle → colors⟦2⟧]]
```

```
In[ ]:= Integrate [fittedFunction , {chemShift , -Infinity , Infinity }]
```

```
In[ ]:= integrateTrapezoidalRule [Re[spectrum]⟦cutPositionUpper2 ;; cutPositionLower2 ⟧, dFH]
```

End of program

```
In[ ]:= Share[]
```

```
In[ ]:= MemoryInUse []
```

```
In[ ]:= MaxMemoryUsed []
```

```
In[ ]:= TimeUsed []
```
